# Mechanism of DOPA radical generation and transfer in metal-free class Ie ribonucleotide reductase based on density functional theory

**DOI:** 10.1016/j.csbj.2022.02.027

**Published:** 2022-03-02

**Authors:** Jinxin Zou, Yao Chen, Wei Feng

**Affiliations:** Department of Biological Engineering, Beijing University of Chemical Technology, Beijing 100029, China

**Keywords:** Density functional theory, Class Ie RNR, DOPA radical, Radical transfer, Superoxide

## Abstract

Quantum mechanical/molecular mechanical (QM/MM) calculations were carried out to investigate the mechanisms of the generation, transfer, and regeneration of the DOPA radical for metal-free class Ie ribonucleotide reductase. The crystal structure of *MfR2* (Nature, 2018, 563, 416–420) was adopted for the calculations. The QM/MM calculations have revealed several key points that are vital for understanding the mechanisms. The superoxide O_2_^•−^ provided by the flavoprotein NrdI cannot directly oxidize the residue Tyr126 to the DOPA radical. It should be protonated to HO_2_^•^. The calculation results suggest that the covalent modification of Tyr126 and the DOPA radical generation can be carried out with no involvement of metal cofactors. This addresses the concerns of the articles (Nature, 2018, 563, 416–420; PNAS, 2018, 115, 10022–10027). Another concern from the articles is that how the DOPA radical is transferred from the radical trap. The DFT calculations have demonstrated that Lys213 is a key residue for the radical transfer from the DOPA radical. The ε-amino group of Lys213 is used not only as a bridge for the electron transfer but also as a proton donor. It can provide a proton to DOPA126 via a water molecule, and thus the radical transfer from DOPA126 to Trp52 is facilitated. It has also been revealed that the protonation of Asp88 is the prerequisite for the DOPA radical generation and the radical transfer in class Ie. Once the radical is quenched, it can be regenerated via the oxidations by superoxide O_2_^•−^ and hydroperoxyl radical HO_2_^•^.

## Highlights

• The mechanism of DOPA radical generation, transfer and regeneration is revealed.
• The superoxide O_2_^•−^ should be protonated to HO_2_^•^ prior to oxidizing Tyr126 to DOPA radical.
• The protonation of Asp88 is the prerequisite for the DOPA radical generation and radical transfer.
• Lys213 is a key residue for the transfer of the DOPA radical.

## Graphical abstract

**Figure:**
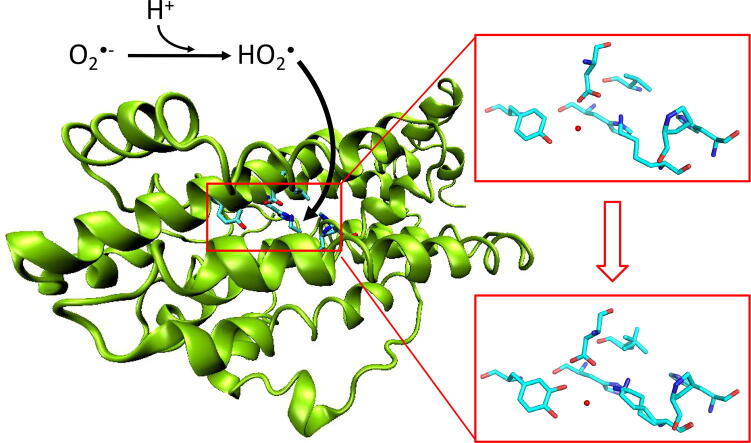

## 1 Introduction

DNA is the carrier of genetic information of life, and the replication and repair of DNA is one of the core links in life. In all known organisms, the four 2′-deoxyribonucleoside triphosphate (dNTPs) substrates used for DNA synthesis are provided by the catalysis of ribonucleotide reductase (RNR). RNR catalyzes the reduction of C2′-OH of ribonucleotides (NDPs/NTPs) to form corresponding deoxyribonucleotides (dNDPs/dNTPs), providing the required precursor for DNA synthesis and repair [1], [2]. RNR is a kind of enzyme that catalyzes reactions through biological organic radicals, and can be grouped into three classes, Ⅰ–III [3], [4], [5], [6], [7], [8], [9], [10], [11], [12], [13]. Class I RNRs are usually composed of two nonidentical dimeric subunits, α subunit (also known as R1 subunit) and β subunit (also known as R2 subunit), and the two subunits interact to form α_2_β_2_ heterotetramer [5]. R1 is the catalytic subunit and R2 is the radical generation subunit. The reaction of ribonucleotide reduction can be started by the cysteinyl radical (Cys•) located in R1 subunit, and this radical is transferred from the tyrosyl radical (Tyr•) in R2 subunit over a long distance (more than 35 Å). Depending on the identities of metal cofactors combined by R2 and the mechanism of radical generation, class I RNRs can be divided into four subclasses: Ia (PMID:4337857), Ib (PMID:20698687), Ic (PMID:15247479), and Id (PMID:29609464) [14], [15], [16], [17]. All those subclasses contain dinuclear metal cofactors [18], [19], [20], [21], [22], [23], [24].

**Fig. 1:**
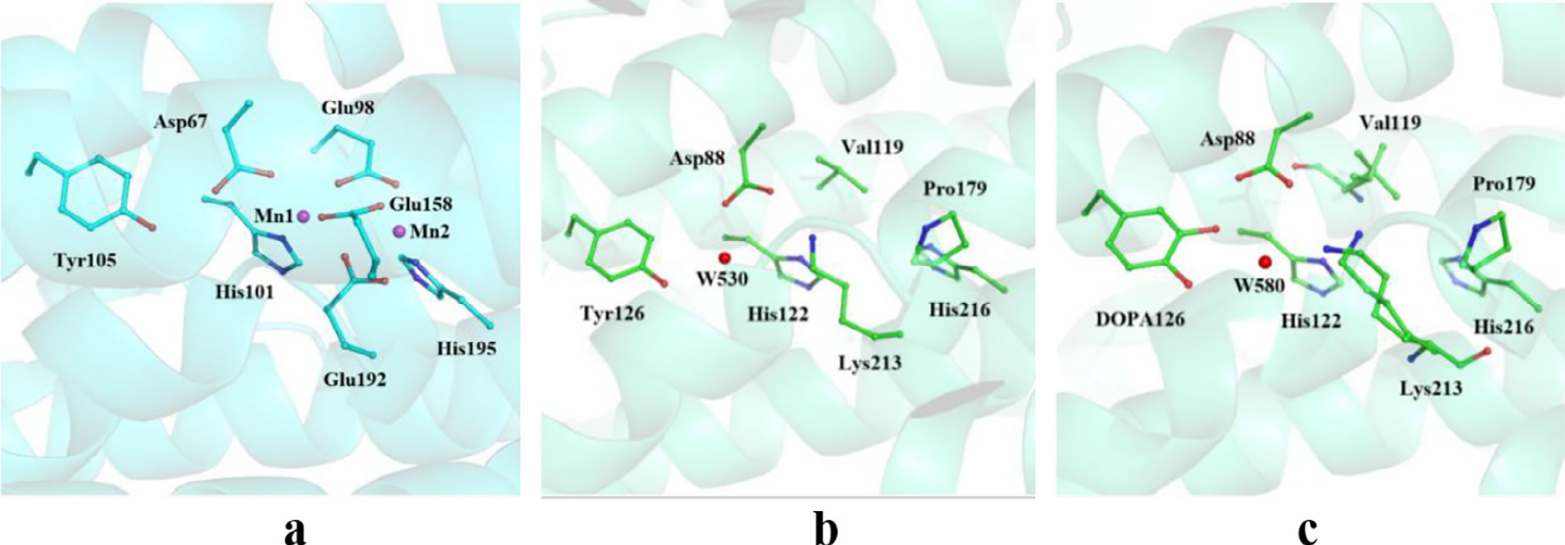
Structures of R2 subunits. (a) Structure of the dinuclear metal site and the conserved metal-coordinating residues in standard class Ib R2 from *E. coli* (PDB ID:3N37). Cyan, red, and blue represent carbon, oxygen, and nitrogen, respectively, and Mn ions are represented by purple spheres. (b) The active site structure of the inactive *MfR2* (PDB: 6GP3). (c) The active site structure of the active *MfR2* (PDB: 6GP2). Green, red, and blue represent carbon, oxygen, and nitrogen, respectively, and water is represented by red sphere.

Since the discovery of RNR, metal cofactors have been considered to be necessary for the generation and stabilization of the catalytic radical in RNR. However, Högbom et al. [25] and Boal et al. [26] have found a group of RNR (class Ie) with a trace amount of metal cofactors in human pathogens. Compared to the central region of the dimanganese cluster in the R2 subunit of Ib enzymes (Fig. 1a), the active site of class Ie consists of an aspartic acid, a valine, a proline, a lysine, and two histidines (Fig. 1b). For class I RNRs, the six residues being coordinated to the transition metal cofactors in R2 subunits are completely conserved. The six conserved residues and the transition metal cofactors are necessary for enzyme activation. While class Ie is the only subclass that is independent of metal cofactor [25], [26]. It is considered to be evolved in response to the extreme metal-limited environments. These changes imply that the mechanism of radical generation and stabilization in class Ie is completely different from that of other subclasses.

In the R2 protein of class Ie, the tyrosine residue can be oxidized to a stable 3,4-dihydroxyphenylalanine (DOPA) radical during enzyme activation, and this radical can initiate the ribonucleotide reduction in vitro and in vivo. The active site structure of the active R2 is shown in Fig. 1c. Covalent modification of the residue tyrosine to form the DOPA radical is a three-electron oxidation process. It is suggested that the superoxide provided by flavoprotein NrdI takes part in the oxidations [25], [26]. How the superoxide participates in the DOPA radical generation is unclear [25], [26]. A trace amount of metal ions are detected in R2 protein. It is not clear whether the metal ions are transiently involved in the generation of the DOPA radical [25], [26].

In the active state of class Ie-R2 subunit, the tyrosine-derived DOPA radical, which serves as the catalytic initiator, is transferred to a cysteine residue in the R1 subunit through a transfer chain when the ribonucleotide reduction reaction is initiated. After the reaction, the radical is then reversibly transferred back to its initial position at the DOPA residue [25], [26]. Stubbe et al. [27] used DOPA as a probe to study the radical transfer chain. They have found that the reduction potential of DOPA was 260 mV lower than that of tyrosine at pH 7.0. It is indicated that DOPA forms a radical trap in the class Ie-R2 subunit. Compared to the tyrosine radical, the DOPA radical is more stable and difficult to be transferred to other residues. Therefore, prior to transferring the DOPA radical, there may be a triggering mechanism that the redox potential difference between DOPA and other residues in the transferring chain is adjusted. This adjustment may be induced by the changes of the protein microenvironment and the conformation of the R2 subunit after binding to R1 subunit. It is no clear that how the redox potential of amino acid residues is adjusted upon the changes of the protein microenvironment and conformation in class Ie. Another concern for Ie RNR is that how to regenerate the DOPA radical directly from the DOPA residue after the radical quenching. Högbom et al. confirmed that once the radical is lost, it can be regenerated by NrdI in an oxygen-dependent process [25]. Hence, the activation step most likely also takes place via superoxide.

As a new discovered RNR enzyme, class Ie is completely different from the previous subclasses in structure and function [25], [26]. Understanding the mechanisms of the generation, transfer, and regeneration of the DOPA radical in class Ie is helpful for the combat of pathogens and the development of anti-pathogen drugs. For this purpose, in this work, density functional theory (DFT) has been used to study the mechanisms of the generation, transfer, and regeneration of the DOPA radical.

## 2 Computational methods

### 2.1 System setup

Two models (models 1 and 2) of the class Ie-R2 subunit were constructed. The initial geometries for building the two models were constructed based on the experimental X-ray crystal structures of chain A of the inactive *MfR2* (PDB: 6GP3, model 1) and the active *MfR2* (PDB: 6GP2, model 2) [25]. The difference of the crystal structures between the inactive and active states is that, 3,4-dihydroxyphenylalanine (DOPA) at the active site of the active state corresponds to the tyrosine residue Tyr126 at the active site of the inactive state. (Fig. 1b, c). The protonation states of the titratable residues were determined at pH 7.0 based on the protein pKa predictor PROPKA 3.0 [28], [29], [30] or based on the neighboring hydrogen bond networks. It is reasonable that all arginine (Arg) and lysine (Lys) residues are in their protonated states, whereas all aspartic acid (Asp) and glutamic acid (Glu) residues are in their deprotonated states with the exception of Asp88 (for asp88, we investigated different protonation states). All histidines (His) are in protonated states. His107 and His122 are protonated at their Nε2 positions, and His143 and His216 are protonated at their Nδ1 positions. For the amino acid chain, the N-terminal is protonated, whereas the C-terminal is deprotonated.

### 2.2 Molecular dynamics (MD) simulations

All classical molecular dynamics simulations were performed using AMBER99SB-ILDN force field [31] with the GROMACS 2019.5 software package [32], [33]. The missing hydrogen atoms were added automatically. The TIP3P model was used for the solvent water molecules [34]. The system was placed in a periodic truncated cubic box, and the closest distance between the surface of the box and the protein atom was set to 8 Å. The protein was hydrated using SPC216 water molecule, and then neutralized by adding ions (Na^+^ and Cl^−^) to generate 0.15 mol/L NaCl solution subsequently. The energy minimization was performed to relax the system using steepest descent and conjugate gradient algorithms. Then, the position restriction equilibrium simulations were conducted in following. (i) The minimized system was heated from 0 to 300 K at constant volume using the velocity-rescale thermostat in a NVT canonical ensemble for 100 ps. (ii) The system was equilibrated in a NPT ensemble for 100 ps, employing the Berendsen barostat at the constant temperature of 300 K and 1 bar. Isotropic pressure coupling was applied with a compressibility of 4.5 × 10^−5^ bar^−1^. All of the solute molecules were restrained to the origin using harmonic potential of 1000 kJ mol^−1^ nm^−2^ during the equilibrium simulation process. Finally, a production MD run was performed for continuous 30 ns in a NPT ensemble with a target pressure of 1 bar and a pressure coupling constant of 2 ps. The periodic boundary conditions were employed in all the simulations, with time integration step size of 2.0 fs. LINCS algorithm was used to constrain bonds involving hydrogen [35]. Long-range electrostatic interactions were calculated using the particle-mesh Ewald (PME) summation algorithm [36] with fast Fourier transform (FFT) grid spacing of 1.6 Å and an cubic interpolation order of 4. Lennard-Jones and short-range Coulomb interaction cut off values were both 10 Å. Frames were collected at 10 ps intervals, and the production trajectories were analyzed using Visual Molecular Dynamics (VMD) [37]. Visualization was performed using PyMOL (the PyMOL Molecular Graphics System, Schrödinger, LLC) and CYLview [38]. RMSD changes in skeleton atoms of protein along the MD trajectories for the inactive and active *MfR2* are shown in Fig. S1.

### 2.3 QM/MM calculations

The initial structure of the QM/MM calculation was taken from the trajectory of the MD simulations. In order to simplify the research model, the solvent molecules beyond 5 Å of the protein were removed. All QM/MM calculations were performed using ONIOM [38], [39], [40], [41] implemented in the Gaussian 09 program [42]. Model 1 was used to study the mechanism of the generation and regeneration of DOPA radical at the active site of the R2 subunit. The active site residues include Asp88, His122, Tyr126, and Lys213, and the neighboring residues are Leu84, Val119, Leu183, Phe187, and Ile209. O_2_^•−^ or HO_2_^•^ are the oxidants. All these constitute the QM region of model 1. Model 2 is used to study the transfer mechanism of DOPA radical between DOPA126 and Trp52. Trp52 is closed to Arg211, Asp212, and W558, they have hydrogen-bonding interactions with His122 of the active site. The residues of the QM region of model 1, Trp52, Arg211, Asp212, W558, and W580, constitute the QM region of model 2. In addition, DOPA126 replaces Tyr126 in model 1. The remaining part of the system was regarded as the MM region. To maintain the contribution of outer residues and water molecules to the system, all amino acid residues and water molecules beyond 6 Å of the QM region were freezed. An electronic embedding scheme [43] was applied to include the polarizing effect of the enzymatic environment on the QM region. Hydrogen link atoms [44] with the charge shift model were employed to treat the QM/MM boundary. The AMBER force field was used for the MM region. The pure QM part was treated by density functional theory (DFT) with the B3LYP exchange–correlation functional method [45], [46], [47], [48]. The B3LYP functional, which is a density-functional theory (DFT) type of calculation based on hybrid functional, has been proved to provide a good balance between speed and accuracy for modeling enzyme reactions [49], [50]. Geometry optimizations were performed using a double-ζ 6-31G (d,p) basis set (labeled B1). All minima were fully optimized without any symmetry restraints. The transition states (TSs) were determined by an initial potential energy surface (PES) scans followed by fully TS optimizations. Frequency calculations were performed using the theory and methodology same to that of geometry optimizations to identify the correct minimum (no imaginary frequency) and transition state (one imaginary frequency) structure, and to obtain zero-point energies (ZPE). To obtain more accurate energies, single-point energies were calculated using a larger all-electron basis set, which is 6-311G (2df,p) for all the atoms (labeled B2). The free energy was obtained based single-point energy, which is corrected by zero-point energy. Based on the ground-state electronic wave functions, the total electrostatic potential (ESP) analysis, charge analysis, spin density analysis, and spin natural orbitals distribution analysis were performed using Multiwfn 3.6 program [51], [52].

Rate constants for the radical transfer between DOPA126 and Trp52 were calculated according to the Marcus-Hush-Levich formula (Eq. (1)) [53], [54], [55], [56], [57], [58], [59].

(1) $$k_{\text{PCET}}=\frac{2\pi }{\hbar }\frac{1}{\sqrt{4\pi \lambda k_{\text{B}}T}}{\left|H_{\text{DA}}\right|}^{2}\exp\left(-\frac{{\left(\lambda +\Delta G\right)}^{2}}{4\lambda k_{\text{B}}T}\right)$$

In this expression, *λ* is the nuclear reorganization energy accompanying electron transfer, *H*_DA_ is electronic coupling matrix element between the donor and acceptor, Δ*G* is the reaction free energy, ℏ is Planck’s constant, *k*_B_ is Boltzmann’s constant, and *T* is temperature. *H*_DA_ is estimated by the method in the articles [53], [60], [61].

## 3 Results and discussion

### 3.1 Generation mechanism of the DOPA radical

#### 3.1.1 Superoxide (O_2_^•−^) as the oxidant when Asp88 in deprotonated state

**Fig. 2:**
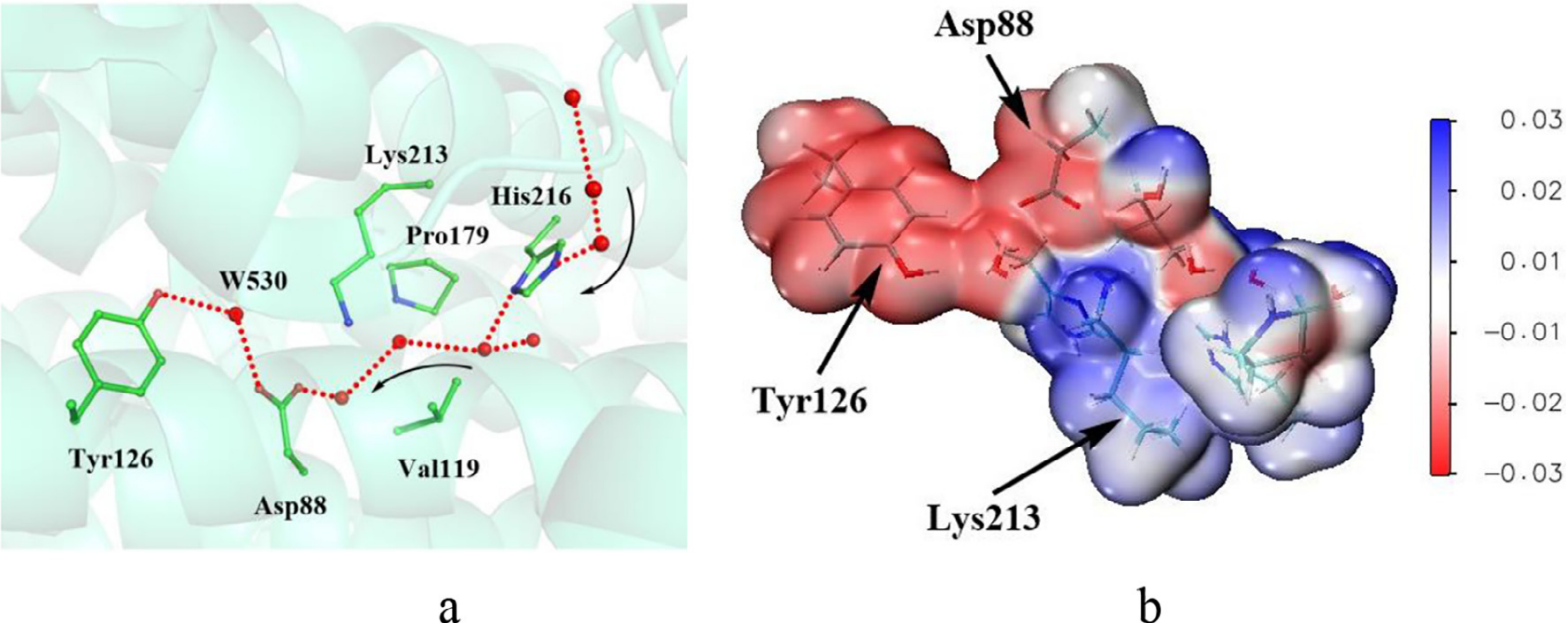
Solvent access channel and total electrostatic potential (ESP). (a) Solvent access channel in the inactive *MfR2* (PDB: 6GP3). Green, red, and blue represent carbon, oxygen, and nitrogen, respectively, and water is represented by red sphere. The dotted line in red represents the solvent access channel. (b) Total electrostatic potential (ESP) for the side chains around the solvent access channel.

Through analysis of the crystal structure of the inactive *MfR2* (PDB: 6GP3), it is found that there is a solvent access channel connecting the active site with NrdI (Fig. 2a). This hydrophilic channel exists in both Ib and Ie subclasses and is used to transport superoxide produced by NrdI [26]. At the end point of the channel, there is a water molecule (W530), which has hydrogen bonding interactions with both Tyr126 and Asp88. Superoxide (O_2_^•−^) is generated by the single-electron reduction of O_2_ under the catalysis of NrdI [62], [63]. The experiments showed that NrdI is involved in the formation of the DOPA radical, therefore O_2_^•−^ is speculated to be the oxidant for the formation of the DOPA radical [25], [26]. Thus, direct oxidation by O_2_^•−^ was first investigated. Once O_2_^•−^ is generated, it enters into the active site via the solvent access channel. Based on analysis of the crystal structure, it is suggested that after O_2_^•−^ enters into the active site, it arrives at the position of W530 and expels the water.

**Fig. 3:**
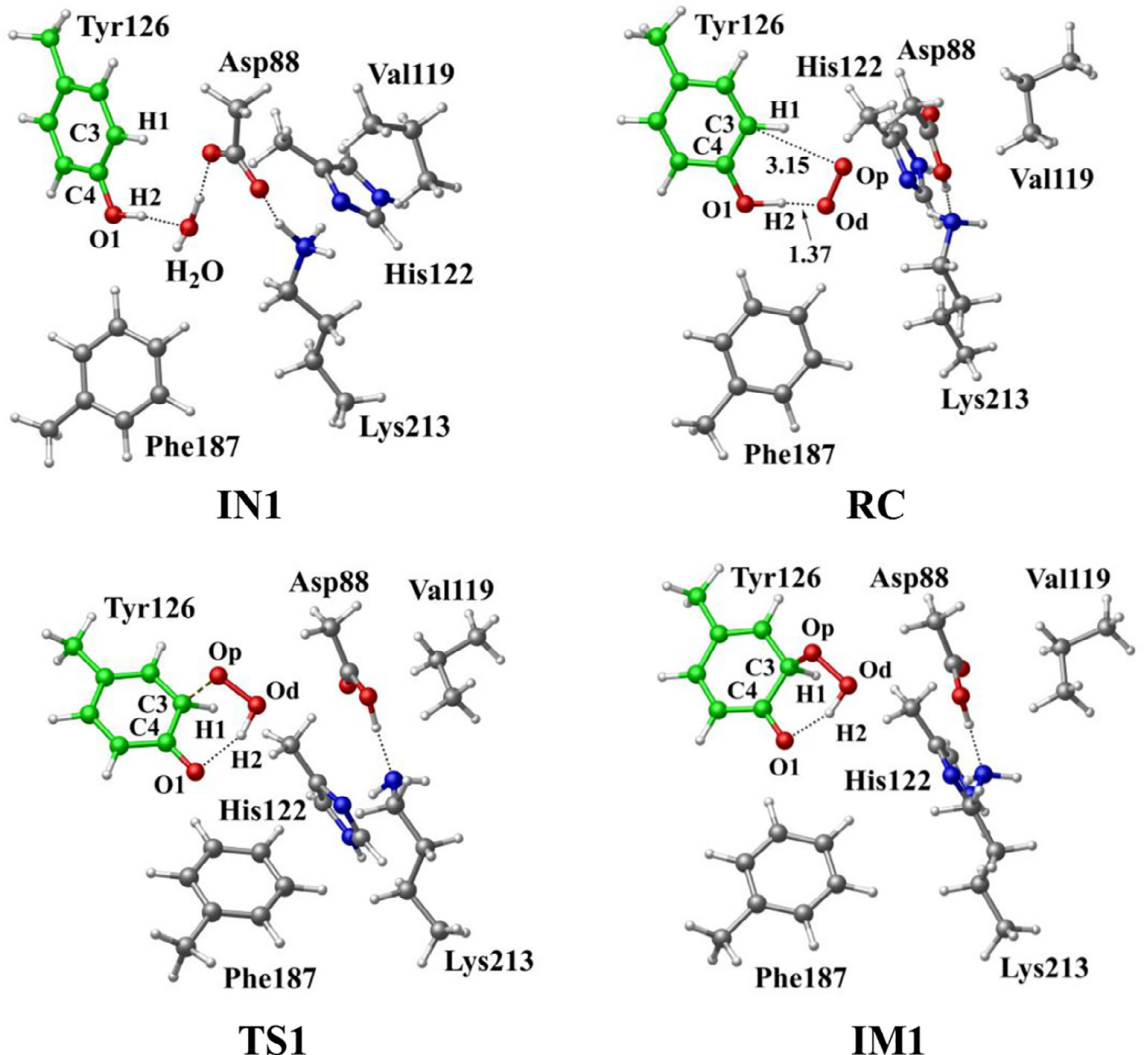
Structures and geometrical parameters for the DOPA radical generation using O_2_^•−^ as the oxidant in the deprotonated state of Asp88. For clarity, only six residues of the model are shown here. The atoms in MM region which linked to QM region are replaced by hydrogens. Red, blue, and white represent oxygen, nitrogen, and hydrogen, respectively. For Tyr126, the carbon atoms and the C–C bonds are shown in green. For other residues, the carbon atoms are shown in gray. The dotted line in black represents hydrogen bond.

**Scheme 1:**
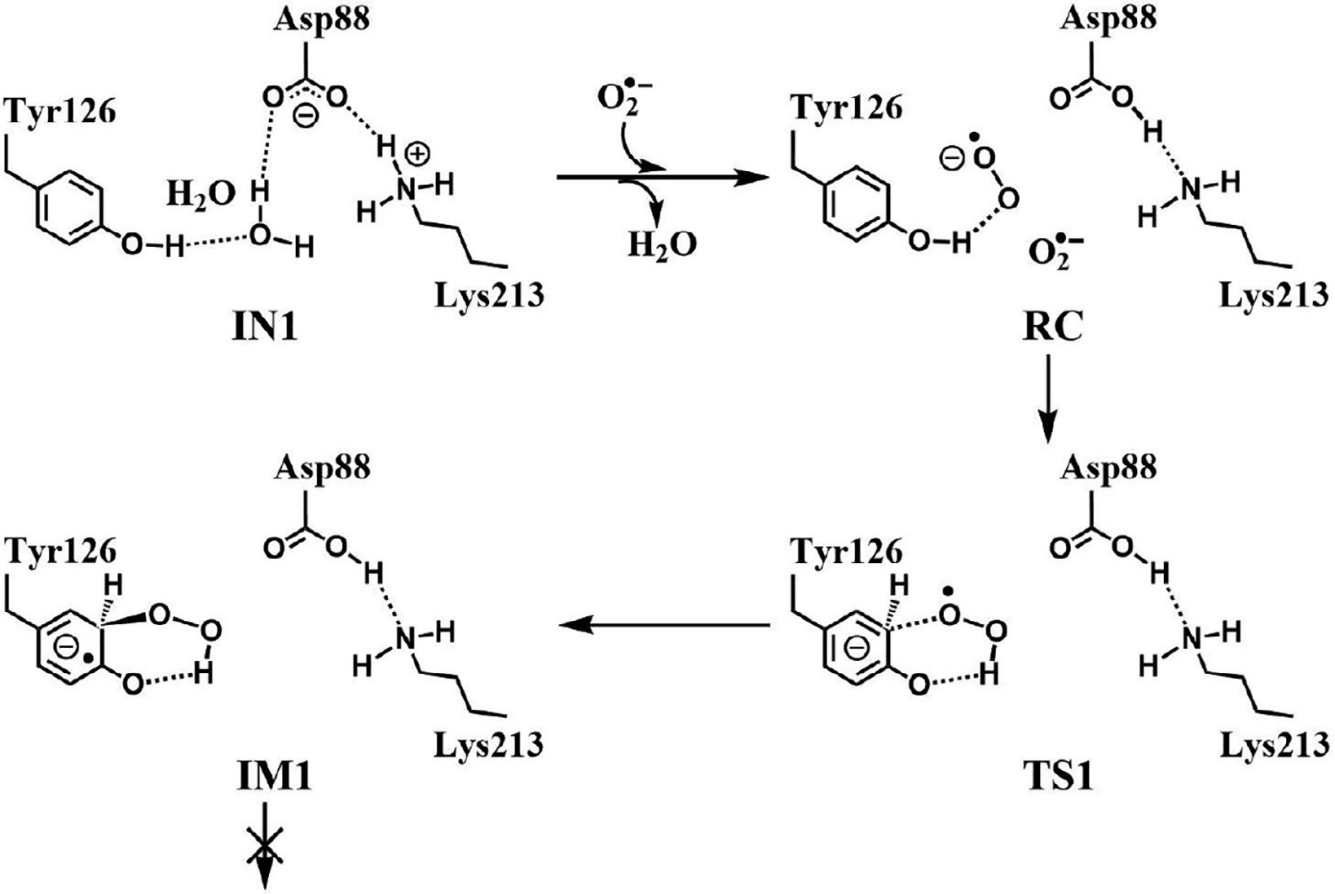
The pathway for the DOPA radical generation using O_2_^•−^ as the oxidant in the deprotonated state of Asp88 based on DFT simulation.

In R2 protein, Asp88 was found to be located in the vicinity of Tyr126 and interact with Tyr126 via a hydrogen-bonded water (W530). Asp88 is an acidic amino acid, and its protonation state is depended on the side chain pKa. According to the protein *pKa* predictor PROPKA 3.0 [28], [29], [30], the *pKa* of Asp88 is 5.86 in the inactive *MfR2* and 6.20 in the active *MfR2*. Therefore, we speculate that Asp88 may exhibit different protonation states in the active center, and the protonation state plays an important role in the radical generation and transfer. In order to analyze the protonation state of Asp88, we carried out MD simulations (30 ns) for the crystal structures in different protonation states. Through the overlay of the initial crystal structures and the structures after the MD simulations, it is found that the structure of Asp88 in deprotonated state is more close to the initial structure after MD simulation (Fig. S2). Therefore, we have simulated the direct oxidation by O_2_^•−^ when Asp88 is in deprotonated state. As illustrated in Fig. 3, the structures have been obtained after optimization, including the initial state when Asp88 is deprotonated (IN1), reactant complex (RC), the first transition state (TS1), and the first intermediate (IM1). In IN1, there are a salt bridge between Asp88 and Lys213 and a hydrogen bond between Asp88 and W530. In RC, O_2_^•−^ enters into the active site and occupies the position of the water molecule (W530). Asp88 and O_2_^•−^ are separated away from each other. The distal oxygen atom Od of O_2_^•−^ forms a hydrogen bond with the phenol OH group of Tyr126 at a distance of 1.37 Å. As the reaction proceeds, O_2_^•−^ moves toward Tyr126. The proximal oxygen atom Op of O_2_^•−^ approaches the meta position (C3′) of Tyr126 and forms a C-O bond through radical substitution reaction. Meanwhile, the phenol proton of Tyr126 moves toward O_2_^•−^ to form HO_2_^•^. Unfortunately, the reaction cannot proceed after IM1 (Scheme 1).

#### 3.1.2 Superoxide (O_2_^•−^) as the oxidant when Asp88 in the protonated state

**Fig. 4:**
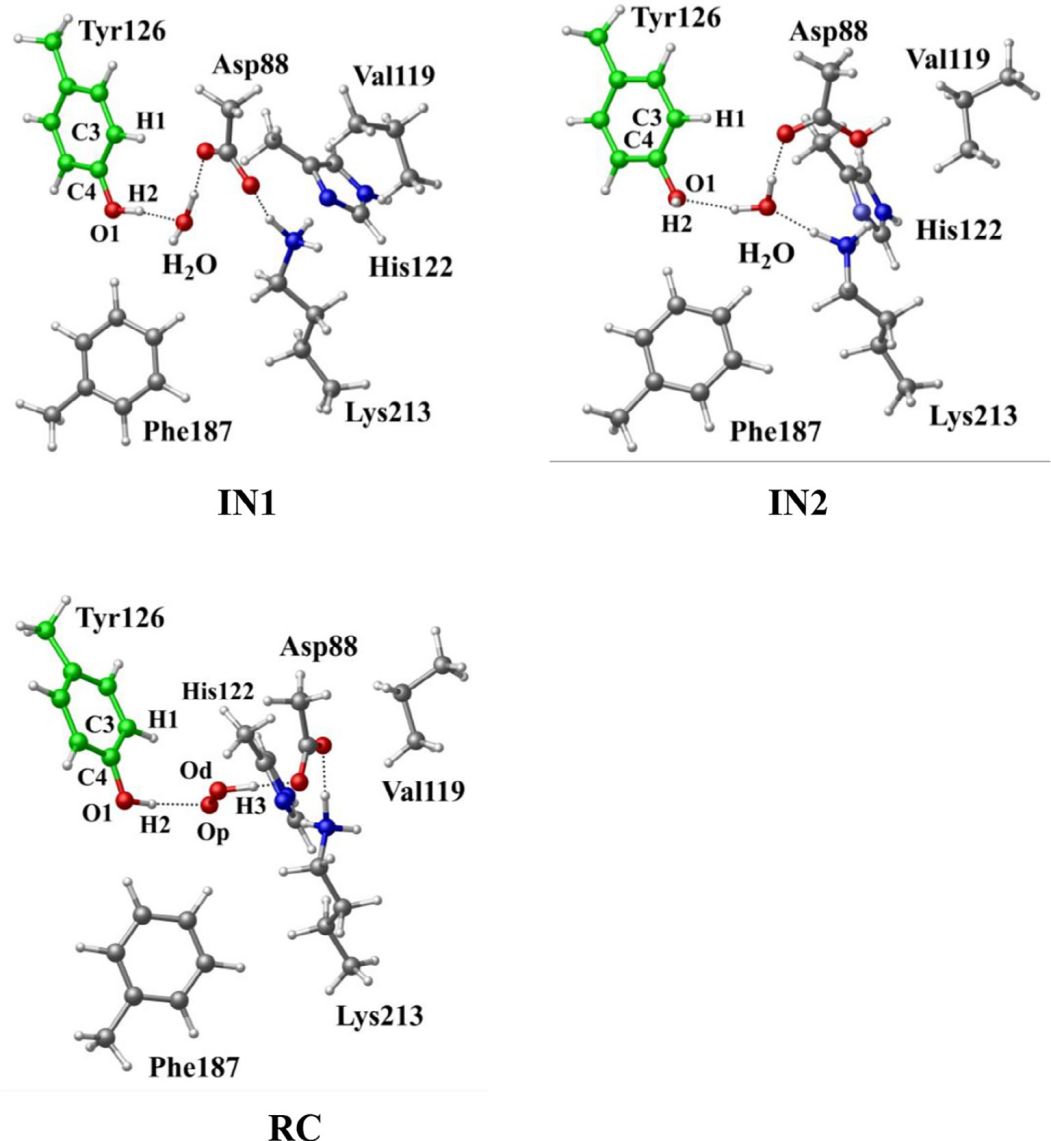
Structures for the DOPA radical generation using O_2_^•−^ as the oxidant in the protonated state of Asp88. For clarity, only six residues of the model are shown here. The atoms in the MM region which is linked to the QM region are replaced by hydrogens. Red, blue, and white represent oxygen, nitrogen, and hydrogen, respectively. For Tyr126, the carbon atoms and the C–C bonds are shown in green. For other residues, the carbon atoms are shown in gray. The dotted line in black represents hydrogen bond.

**Scheme 2:**
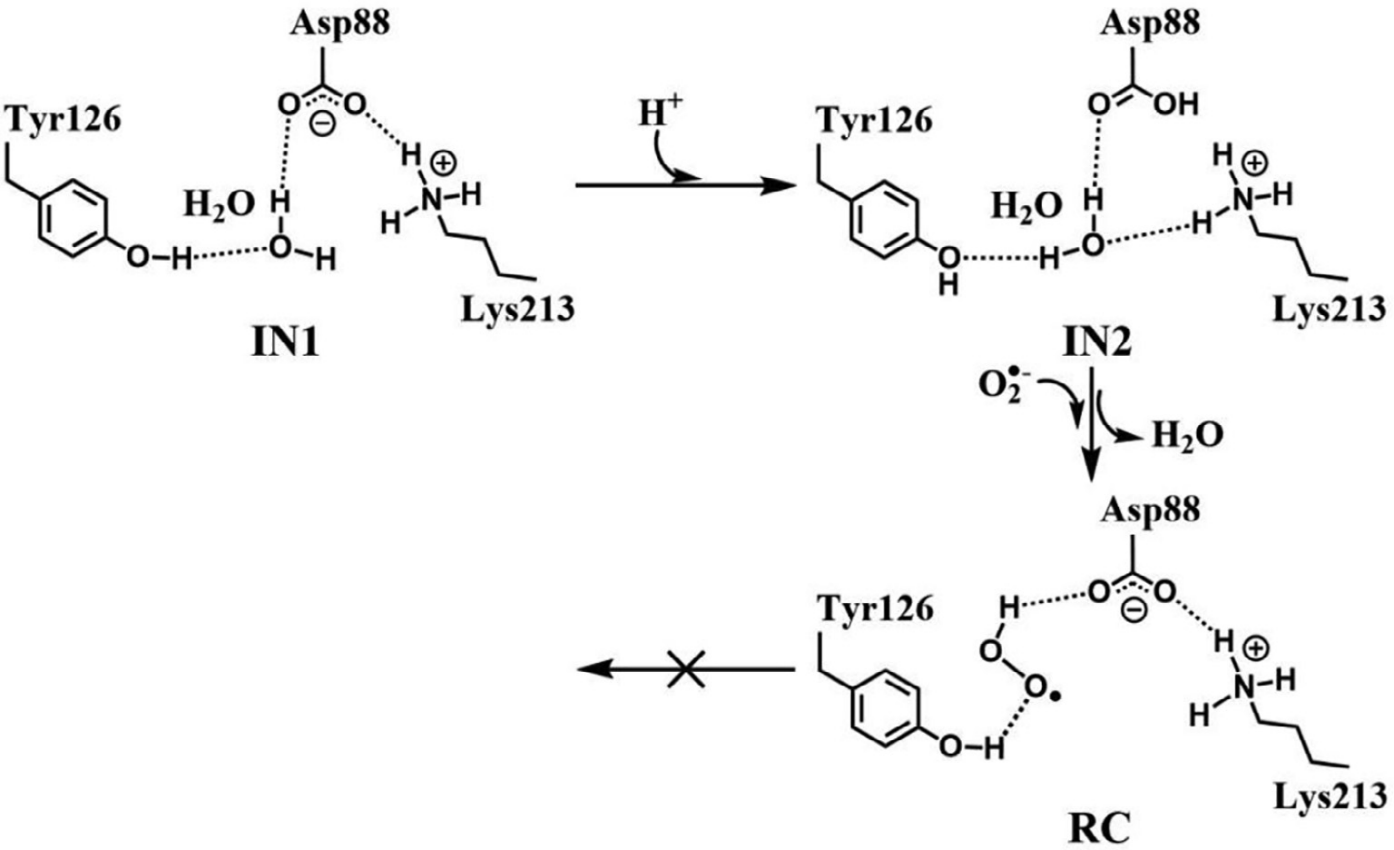
The pathway for the DOPA radical generation using O_2_^•−^ as the oxidant in the protonated state of Asp88 based on DFT simulation.

Then we carried out the calculations for the direct oxidation by O_2_^•−^ when Asp88 is protonated. The optimized geometries are shown in Fig. 4. The salt bridge between Asp88 and Lys213 disappears when Asp88 is protonated in IN2. As O_2_^•−^ enters the active site, it abstracts the proton from Asp88 due to its strong electronegativity as indicated in RC. However, the oxidation reaction cannot proceed after RC as illustrated in Scheme 2.

#### 3.1.3 Hydroperoxyl radical (HO_2_^•^) as the oxidant when Asp88 in deprotonated state

**Fig. 5:**
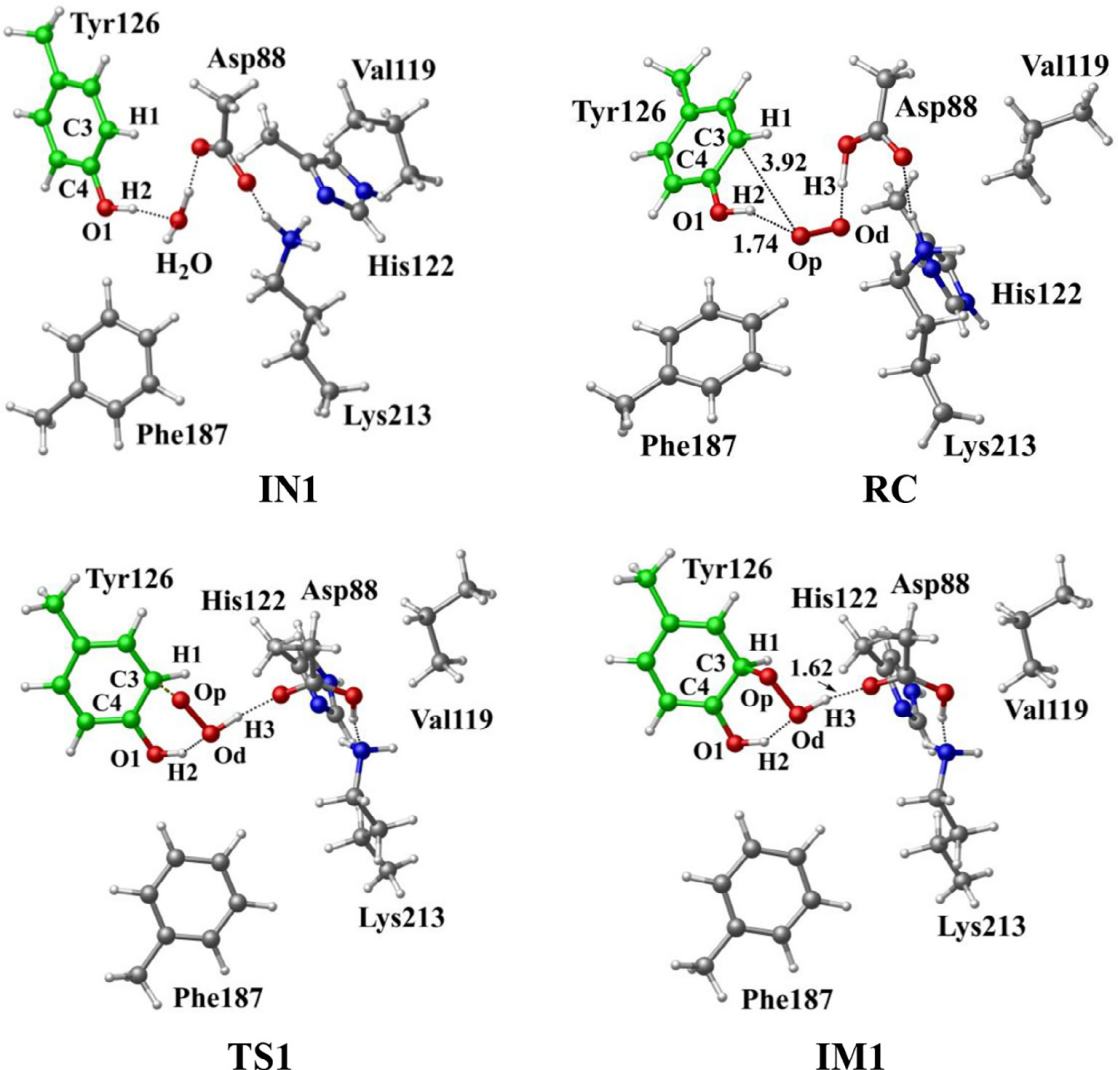
Structures and geometrical parameters for the DOPA radical generation using HO_2_^•^ as the oxidant in the deprotonated state of Asp88. For clarity, only six residues of the model are shown here. The atoms in the MM region which is linked to the QM region are replaced by hydrogens. Red, blue, and white represent oxygen, nitrogen, and hydrogen, respectively. For Tyr126, the carbon atoms and the C–C bonds are shown in green. For other residues, the carbon atoms are shown in gray. The dotted line in black represents hydrogen bond.

**Scheme 3:**
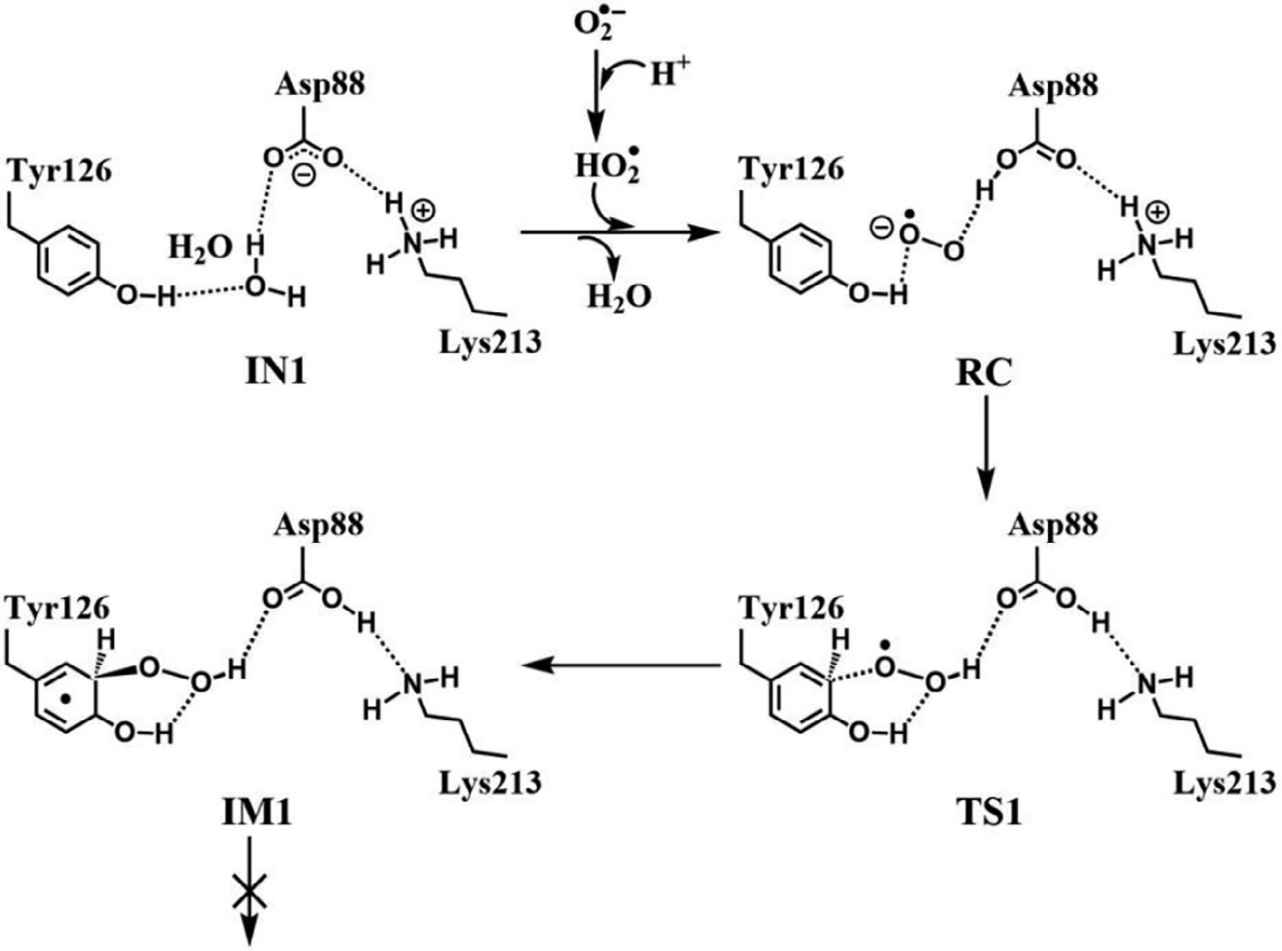
The pathway for the DOPA radical generation using HO_2_^•^ as the oxidant in the deprotonated state of Asp88 based on DFT simulation.

The above results have demonstrated that, no matter Asp88 is protonated or deprotonated, through the direct oxidation by O_2_^•−^, the DOPA radical cannot be formed. O_2_^•−^ can be protonated to form hydroperoxyl radical (HO_2_^•^) (O_2_^•−^ + H^+^ → HO_2_^•^), and this protonation reaction is reversible [64], [65], [66]. As an oxidant, HO_2_^•^ has been shown to have a stronger oxidation capability than O_2_^•−^ [67]. Thus, we started the simulation with HO_2_^•^ as the oxidation. The optimized structures and geometries of the key steps of the oxidation process are shown in Fig. 5. HO_2_^•^ gradually approaches the meta position (C3′) of Tyr126 and the C-O bond is formed through the radical substitution reaction. Then the dioxygen bond breaks, in the meantime the distal oxygen atom Od abstracts a proton to form a water molecule. However, after IM1 as illustrated in Scheme 3, the oxidation cannot be proceeded. It is ascribed to that, in IM1, HO_2_^•^ is hydrogen bonded to Asp88 in deprotonated state with a distance of 1.62 Å, and the strong hydrogen bonding interaction restricts the movement of Od.

#### 3.1.4 Hydroperoxyl radical (HO_2_^•^) as the oxidant when Asp88 in protonated state

**Fig. 6:**
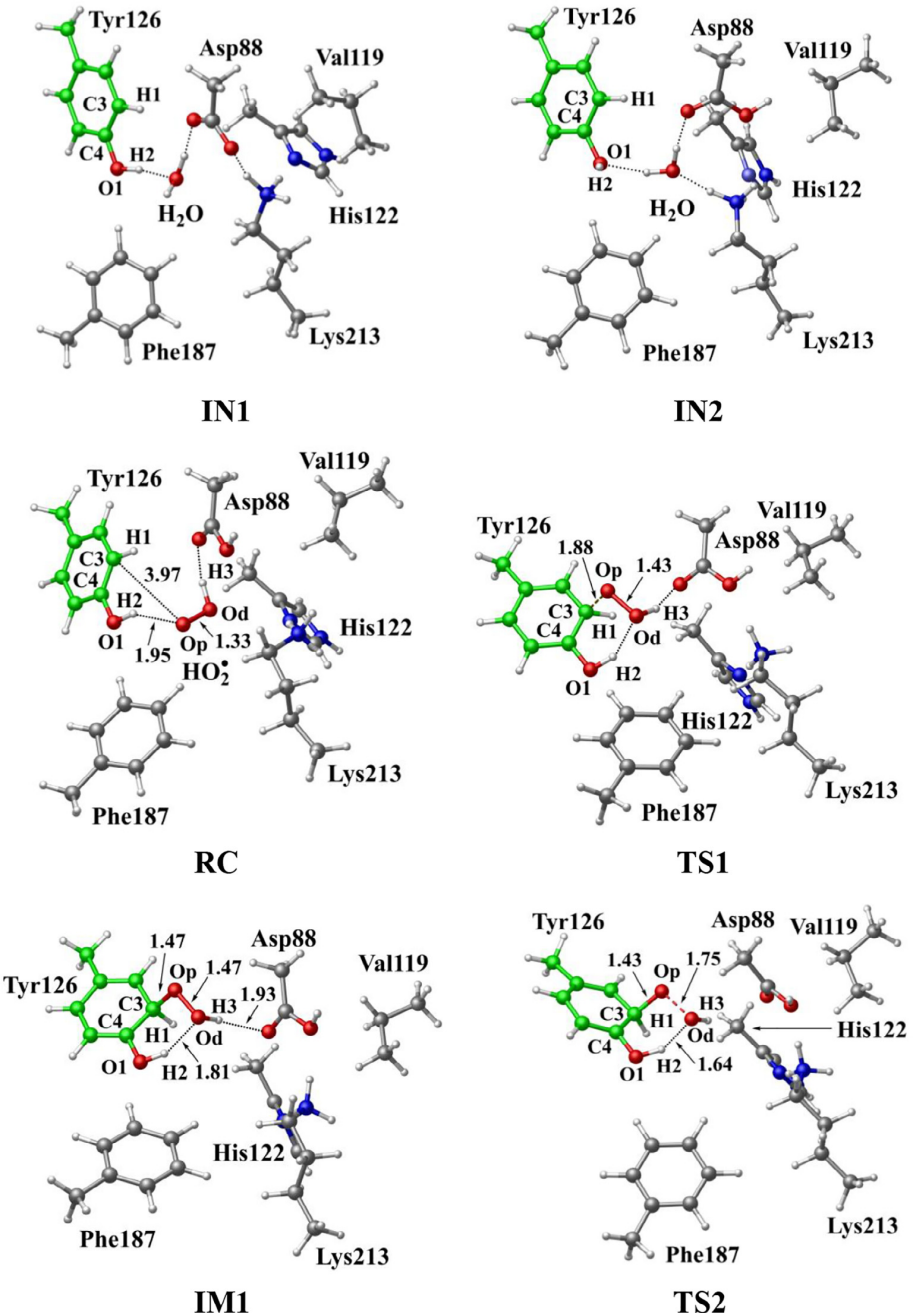

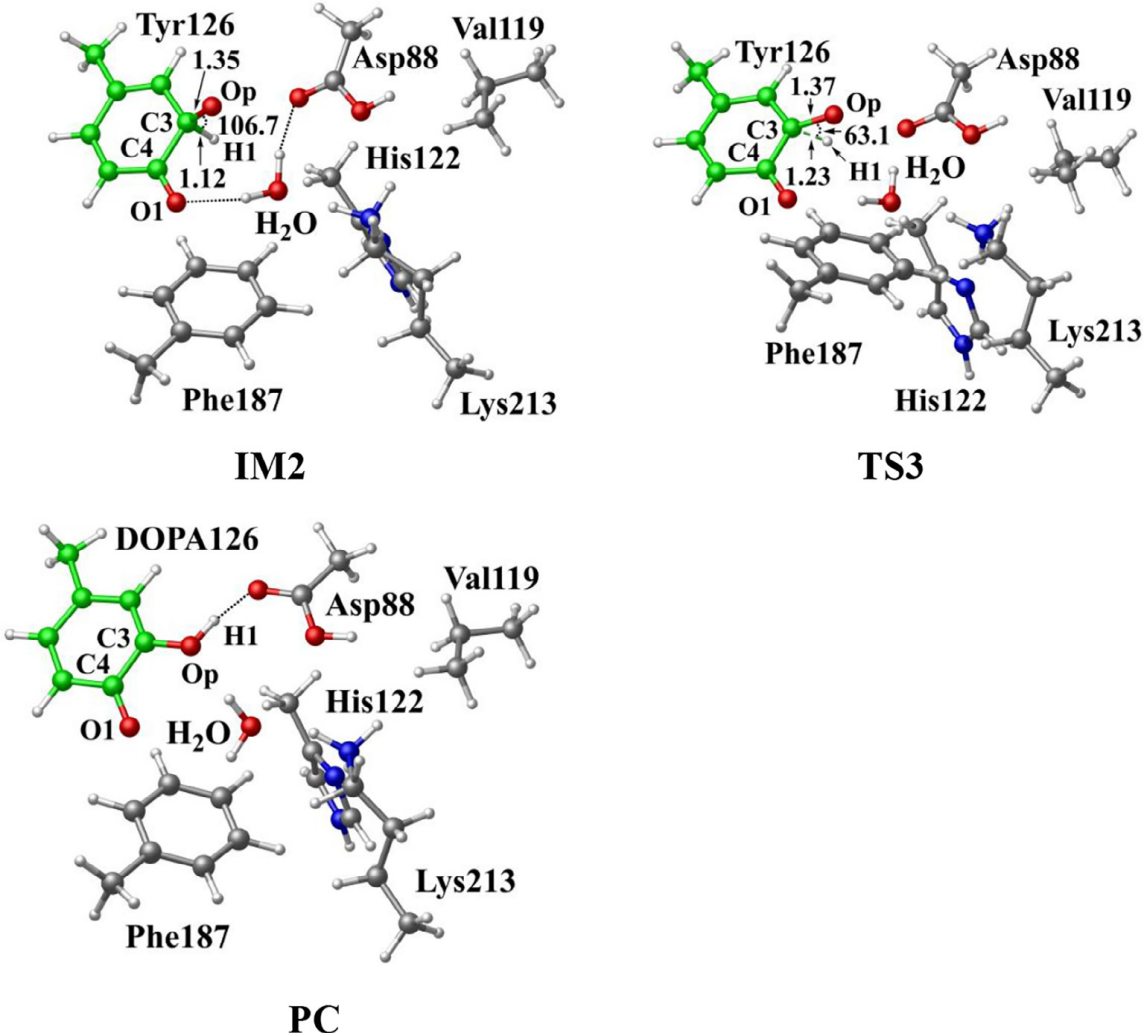
Structures and geometrical parameters for the DOPA radical generation using HO_2_^•^ as the oxidant in the protonated state of Asp88 (pathway A). For clarity, only six residues of the model are shown here. The atoms in the MM region which is linked to the QM region are replaced by hydrogens. Red, blue, and white represent oxygen, nitrogen, and hydrogen, respectively. For Tyr126/DOPA126, the carbon atoms and the C–C bonds are shown in green. For other residues, the carbon atoms are shown in gray. The dotted line in black represents hydrogen bond. The length is in Angstrom and angle in Degree.

**Fig. 7:**
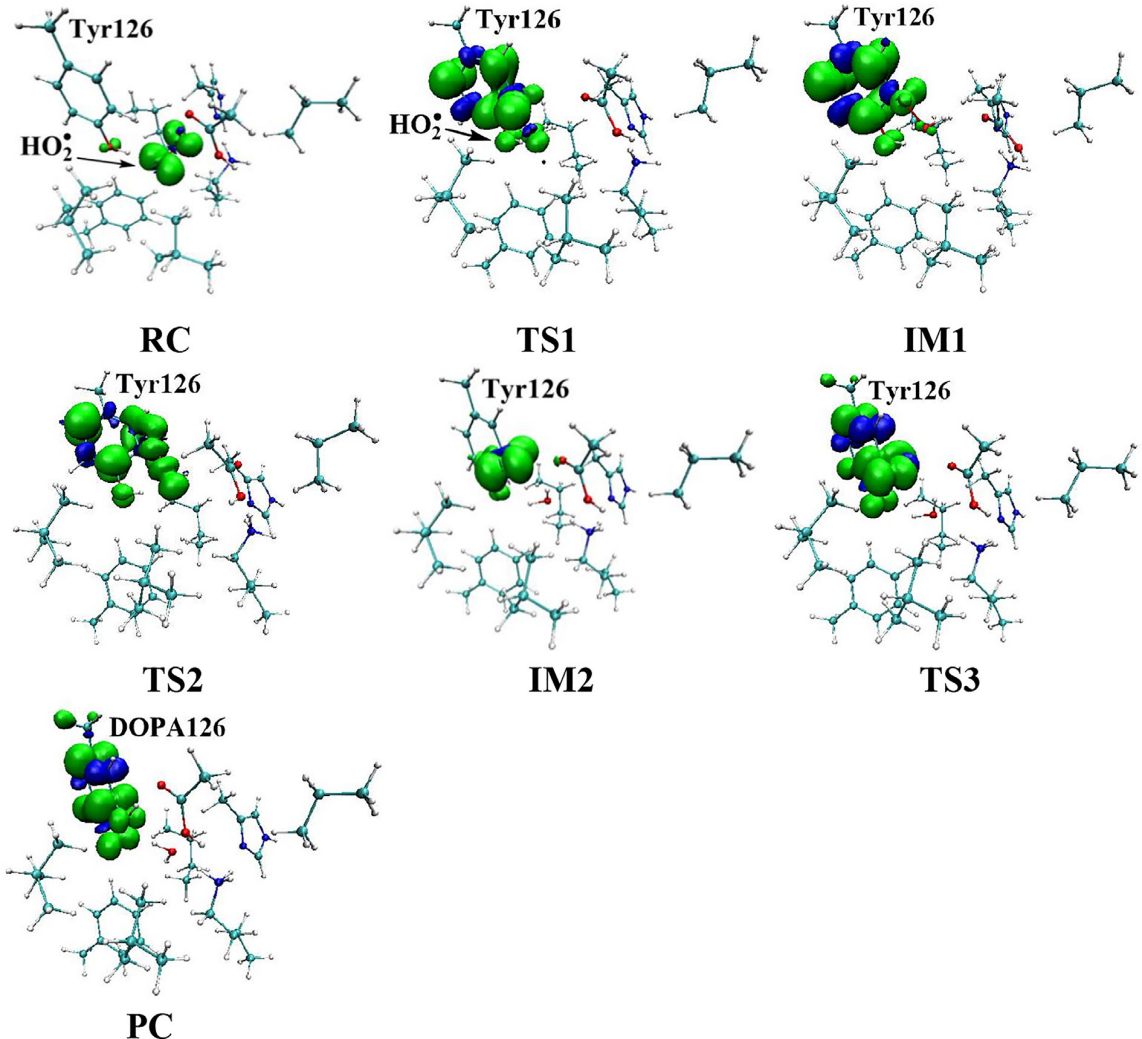
Spin density distributions for the DOPA radical generation using HO_2_^•^ as the oxidant in the protonated state of Asp88 (pathway A). Blue and green represent the negative and positive spin density, respectively.

**Fig. 8:**
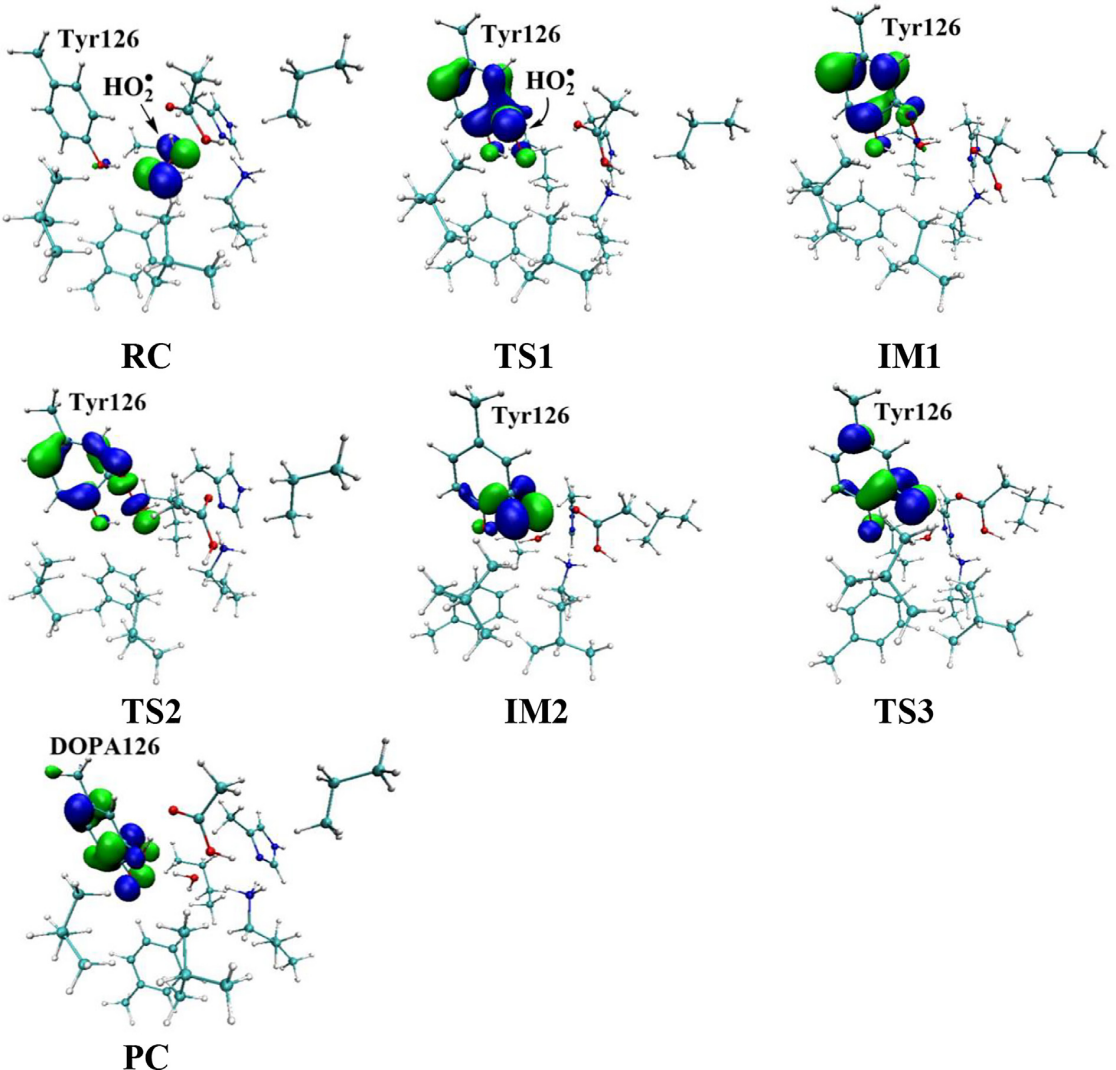
Spin natural orbitals distribution of unpaired single-electron for the DOPA radical generation using HO_2_^•^ as the oxidant in the protonated state of Asp88 (pathway A). Blue and green represent the negative and positive spin density, respectively.

Further calculations were carried out when Asp88 is in protonated state, and the optimized structures and geometries are shown in Fig. 6. In the reactant complex RC, the proximal oxygen atom Op forms a hydrogen bond with the phenol OH group of Tyr126 at a distance 1.95 Å. According to the spin density analysis (Table 1, Fig. 7, Fig. 8), the unpaired single-electron is mainly located on the dioxygen of HO_2_^•^, with the spin densities of Op and Od being 0.68 and 0.30, respectively. Due to possessing an unpaired single-electron, HO_2_^•^ has a strong oxidation reactivity.

**Table 1:** Atomic dipole corrected hirshfeld (ADCH) atomic charges (unit: C), spin densities and spin natural orbitals (SNO) distribution of unpaired single-electron for the DOPA radical generation using HO_2_^•^ as the oxidant in the protonated state of Asp88 (pathway A).

		HO_2_^•^	Tyr126/DOPA126	Op	Od	O1
ADCH charge	RC	0.00	0.02	−0.08	−0.18	−0.12

	TS1	−0.20	0.22	−0.20	−0.18	−0.27
IM1	−0.14	0.03	−0.16	−0.25	−0.23
	TS2	—	0.20	−0.22	−0.37	−0.23
	IM2	—	0.16	−0.16	−0.45	−0.23
	TS3	—	0.15	−0.19	−0.44	−0.24
	PC	—	0.08	−0.16	−0.51	−0.28
Spin density	RC	0.98	0.01	0.68	0.30	0.01
	TS1	0.40	0.60	0.36	0.04	0.06
	IM1	0.04	0.99	0.03	0.01	0.07
	TS2	—	0.77	0.21	0.23	0.04
	IM2	—	0.98	0.79	0.00	0.04
	TS3	—	0.99	0.50	0.00	0.18
	PC	—	0.99	0.06	0.00	0.35
SNO distribution	RC	92%	3%	62%	30%	1%
	TS1	35%	64%	29%	5%	6%
	IM1	—	98%	4%	1%	6%
	TS2	—	79%	22%	18%	4%
	IM2	—	96%	69%	0	3%
	TS3	—	97%	40%	0	10%
	PC	—	98%	6%	0	25%

The oxidation reaction starts with HO_2_^•^ moving toward the aromatic ring of Tyr126. HO_2_^•^ approaches the meta position (C3′) of Tyr126 forming the C-O bond. This step undergoes the first transition state (TS1) and the first intermediate (IM1). As HO_2_^•^ approaches Tyr126, the C3-Op distance is shortened from 3.97 Å in RC to 1.88 Å in TS1. Meanwhile, Od departs from Op, and the Od-Op distance is elongated from 1.33 Å in RC to 1.43 Å in TS1. In IM1, the C3-Op bond is formed with a bond length of 1.47 Å. In the meantime, Od further departs from Op, and the Od-Op distance is 1.47 Å. The spin density on HO_2_^•^ is changed from 0.98 in RC to 0.04 in IM1, and the spin density on Tyr126 increases from 0.01 in RC to 0.99 in IM1 (Table 1, Fig. 7). The unpaired spin density located HO_2_^•^ is almost transferred to Tyr126. This step is endothermic. The calculated free energy barrier is 15.5 kcal/mol and the reaction free energy is 7.1 kcal/mol.

In the second step (IM1 → IM2), the distal oxygen atom Od abstracts the phenol proton of Tyr126, with the formation of a water molecule. In IM1, the hydrogen bond distance between HO_2_^•^ and the carboxyl group of Asp88 is 1.93 Å. This hydrogen bond is very weak, hence has little effect on the oxidation reaction. From IM1 to IM2, the Od-Op distance is gradually elongated and the Od-H2 distance is gradually shortened. In the intermediate IM2, the Od-Op and O1-H2 bonds break simultaneously, and the Od-H2 bond is formed with the formation of a water molecule. This step is exothermic. The free energy barrier is 10.5 kcal/mol, and the reaction free energy is −42.9 kcal/mol.

In IM2, the unpaired single-electron is essentially located on Op with a spin density of 0.79 (Table 1, Fig. 7), revealing that Op has a strong activity and can receive a hydrogen. Therefore, the third step (IM2 → PC) involves the abstraction of hydrogen H1 by Op to generate the C3′ hydroxyl group. This step undergoes the third transition state (TS3). In IM2, the distances of the C3-Op and C3-H1 bonds are 1.35 Å and 1.12 Å, respectively, and the angle ∠Op-C3-H1 is 106.7°. Due to the abstraction by Op, H2 moves toward Op, and the angle ∠Op-C3-H1 gradually becomes smaller. In TS3, the angle ∠Op-C3-H1 becomes 63.1°, and the distances of the C3-Op and C3-H1 bonds are elongated to 1.37 Å and 1.23 Å, respectively. Upon the generation of the hydroxyl group, the DOPA radical is finally formed in the product complex (PC). In PC, the charge and spin density of the oxygen atom O1 are −0.28 and 0.35, respectively (Table 1). 25% of the unpaired single-electron is located on O1 (Table 1, Fig. 8g), confirming the formation of phenoxyl-radical. In the final step, the calculated free energy barrier is 18.3 kcal/mol, and the reaction free energy is −43.4 kcal/mol, revealing that this step is highly exothermic.

**Scheme 4:**
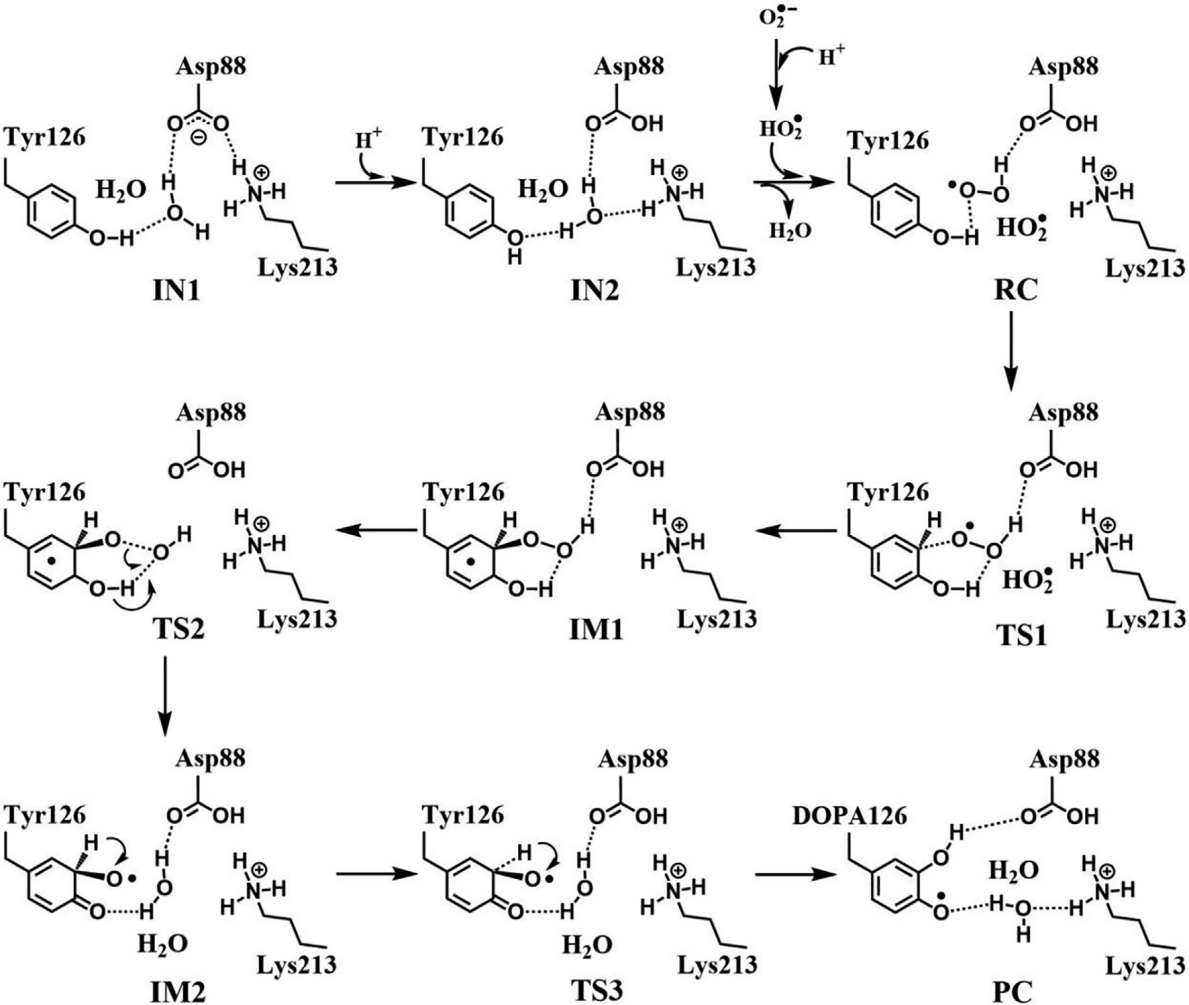
The pathway for the DOPA radical generation based on DFT simulation (pathway A).

**Fig. 9:**
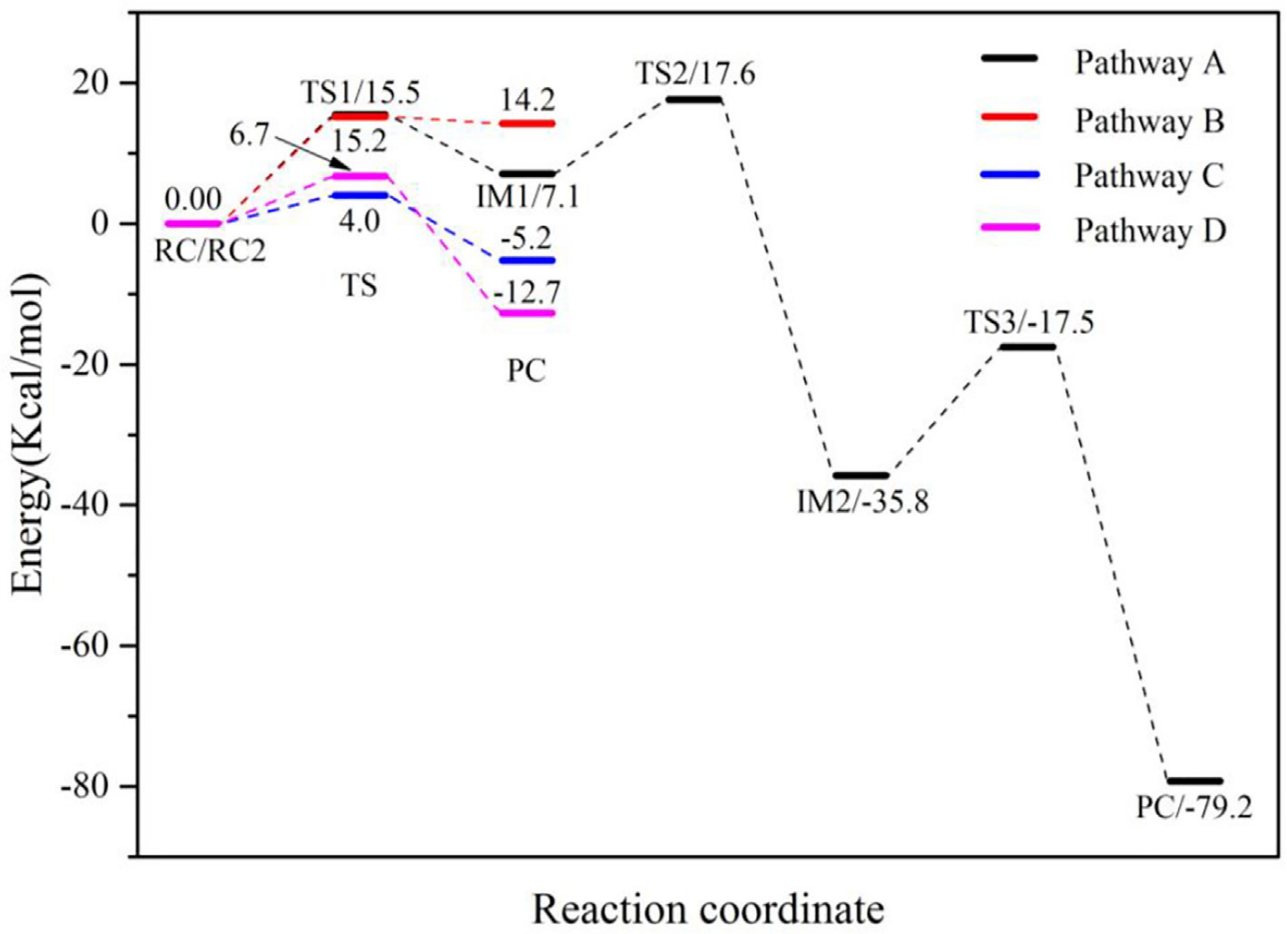
Potential energy profile of pathways for the DOPA radical generation (pathway A), transfer (pathway B) and regeneration (pathway C, D) based on DFT simulation.

Scheme 4 illustrates the mechanism of the DOPA radical generation. Based on the DFT simulations, it has been found that HO_2_^•^, not O_2_^•−^, can directly oxidize Tyr126 to form the DOPA radical. It is suggested that the oxidation does not need the participation of metal ions. The reaction is a three-electron oxidation, which undergoes the tyrosine meta-hydroxylation and the DOPA radical generation. The calculation results are consistent with that proposed by Högbom et al. [25]. Fig. 9 shows the calculated potential energy profile for the pathway A, which undergoes three steps after RC. The third step is the rate-determining step, and it is exothermic. The calculated rate constant in the third step is 1.43 × 10^2^ s^−1^, which is close to the experimental reaction rate of HO_2_^•^ with phenylalanine <180.0 ± 50.0 s^−1^ [68]. The total reaction energy of the pathway is −79.2 kcal/mol. Therefore, the reaction pathway is thermodynamically favorable. It is noticed that in the pathway, the DOPA radical, not DOPA, is directly formed.

When Asp88 is in the deprotonated state, the oxygen atom of the carboxyl group can form a strong hydrogen bond with the surrounding hydrogen atom. The hydrogen bonding interaction is not favorable for the oxidation reaction. Whereas Asp88 in the protonated state, the hydrogen bonding interaction is significantly weakened. Therefore, the prerequisite of the oxidation reaction is that the side chain of Asp88 is protonated. According to the *pKa* of Asp88 of the inactive *MfR2* and the MD simulation of the initial crystal structure (Fig. S2), Asp88 is likely to be deprotonated in the initial state. Therefore, there may be a switch mechanism in class Ie. Once the reaction of the DOPA radical generation is initiated, the switch mechanism can facilitate Asp88 to be protonated. Previous researches have proposed a histidine switch mechanism for viral membrane fusion [69], [70], [71]. This mechanism shows that, in many viral fusion proteins, histidines are located in the vicinity of positively charged residues in the prefusion conformation, and are singly protonated at neutral pH. However, once stimulated by external conditions such as mildly acidic conditions (pH 4.5–6.5), that are close to the *pKa* of histidine side-chain protonation, histidines are doubly protonated and positively charged. Many proteins make use of conformational changes to achieve their functional purposes [72]. The conformational change is usually coupled with the protonation states of ionizable residues such as Lys, Glu, Asp, and Arg residues, and the coupling is an effective regulation mechanism of proteins [72]. The internal ionizable residues would adjust their protonation states and *pKa* values in different microenvironments. For aspartic acids, when they are exposed to a hydrophilic region, they exhibit deprotonation states. However, when they are located in a relatively hydrophobic region of the protein, their *pKa* values would tend to rise, and they exhibit neutral protonation states. In the active site of the R2 subunit, Asp88 is located in vicinity of the ε-amino group of Lys213. As shown in Fig. 2b, the total electrostatic potential (ESP) around the ε-amino group of Lys213 is positive. Based on the protein *pKa* predictor PROPKA 3.0 [28], [29], [30], the *pKa* of Asp88 of the inactive *MfR2* is 5.86, and that of the active *MfR2* is 6.20. Therefore, it is speculated that there is a “switch phenomenon” in class Ie. When the flavoprotein NrdI binds to the R2 subunit, the microenvironment of the active site is changed from neutral hydrophilic to mildly acidic and relatively hydrophobic, leading to that the *pKa* of Asp88 is increased from 5.86 to 6.20. In the meantime, Asp88 turns to be in a protonated state. After the generation of the DOPA radical, R2 separates from NrdI. The microenvironment of the active site is changed from relatively hydrophobic to hydrophilic, and the *pKa* of Asp88 is decreased to 5.86. Thus, Asp88 is deprotonated and returned to its initial state. The possible reason for this mechanism is that when the protein aggregates, the solvent channel between the active site and the external aqueous solution is blocked to some extent, the active site of protein becomes more hydrophobic.

O_2_^•−^ can be protonated to form hydroperoxyl radical HO_2_^•^. Therefore, like the protonation of Asp88, it is speculated that when R2 is bonded to NrdI, the microenvironment of the active site turns to be mildly acidic, which benefits the protonation of O_2_^•−^. In the active site, the positively charged ε-amino group of Lys213 is located in the vicinity of the solvent access channel (Fig. 2a). We speculate that O_2_^•−^ can be protonated to HO_2_^•^ at such a mildly acidic condition. HO_2_^•^ has been demonstrated to be more reactive than O_2_^•−^ [67]. It is reasonable that HO_2_^•^ is the oxidant that oxidizes the side chain of Tyr126 to form the DOPA radical.

### 3.2 Transfer mechanism of the DOPA radical

**Fig. 10:**
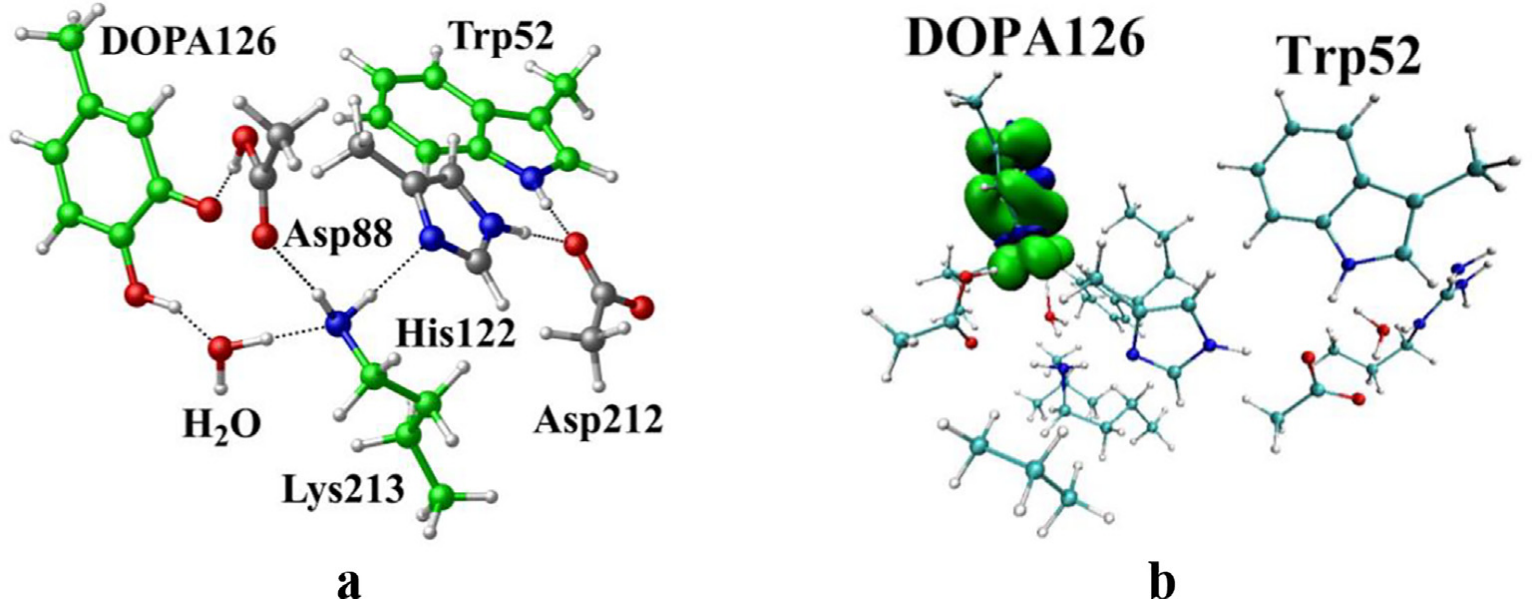
(a) Structure for the DOPA radical transfer between DOPA126 and Trp52 in the deprotonated state of Asp88. For clarity, only six residues of the model are shown here. The atoms in the MM region which is linked to the QM region are replaced by hydrogens. Red, blue, and white represent oxygen, nitrogen, and hydrogen, respectively. For DOPA126, Lys213 and Trp52, the carbon atoms and the C–C bonds are shown in green. For other residues, the carbon atoms are shown in gray. (b) Pin density distribution. Blue and green represent the negative and positive spin density, respectively.

The radical transfer in proteins is accomplished through a proton-coupled electron transfer (PCET) mechanism [73]. Electron is transferred by a long-range electron tunneling. In biological proteins, the amino acids, which can serve as waystations for electron tunneling (hopping) reactions, are almost tyrosine and tryptophan residues [74]. After careful analysis, it is found that Trp52 is the first electron waystation nearby DOPA126. So, when the DOPA radical transfer is initiated, an electron located on Trp52 is transferred to the phenol group of DOPA126 over the radical transfer chain, creating a cationic tryptophan radical Trp52^+•^. The radical transfer from DOPA126 to Trp52 is the first and most important step. The radical transfer in the deprotonated state of Asp88 has been studied. However, we could not obtain Trp52^+•^ after optimization. As shown in Fig. 10a, the hydrogen on C3-OH of DOPA126 has been transferred to the carboxyl group of the side-chain of Asp88. This leads to that the radical is still on DOPA126 and cannot be transferred to Trp52, although the C4′ phenoxyl radical (C-O•) has been protonated. Fig. 10b shows that Trp52 has no spin density. This result is consistent with the opinion of Boal et al. [26], who proposed that the retention of this hydrogen is critical for tuning of the redox potential of the DOPA radical. Hence, in order to hold the hydrogen of C3-OH on its position, Asp88 should be protonated before the initiation of the proton/electron transfer.

**Fig. 11:**
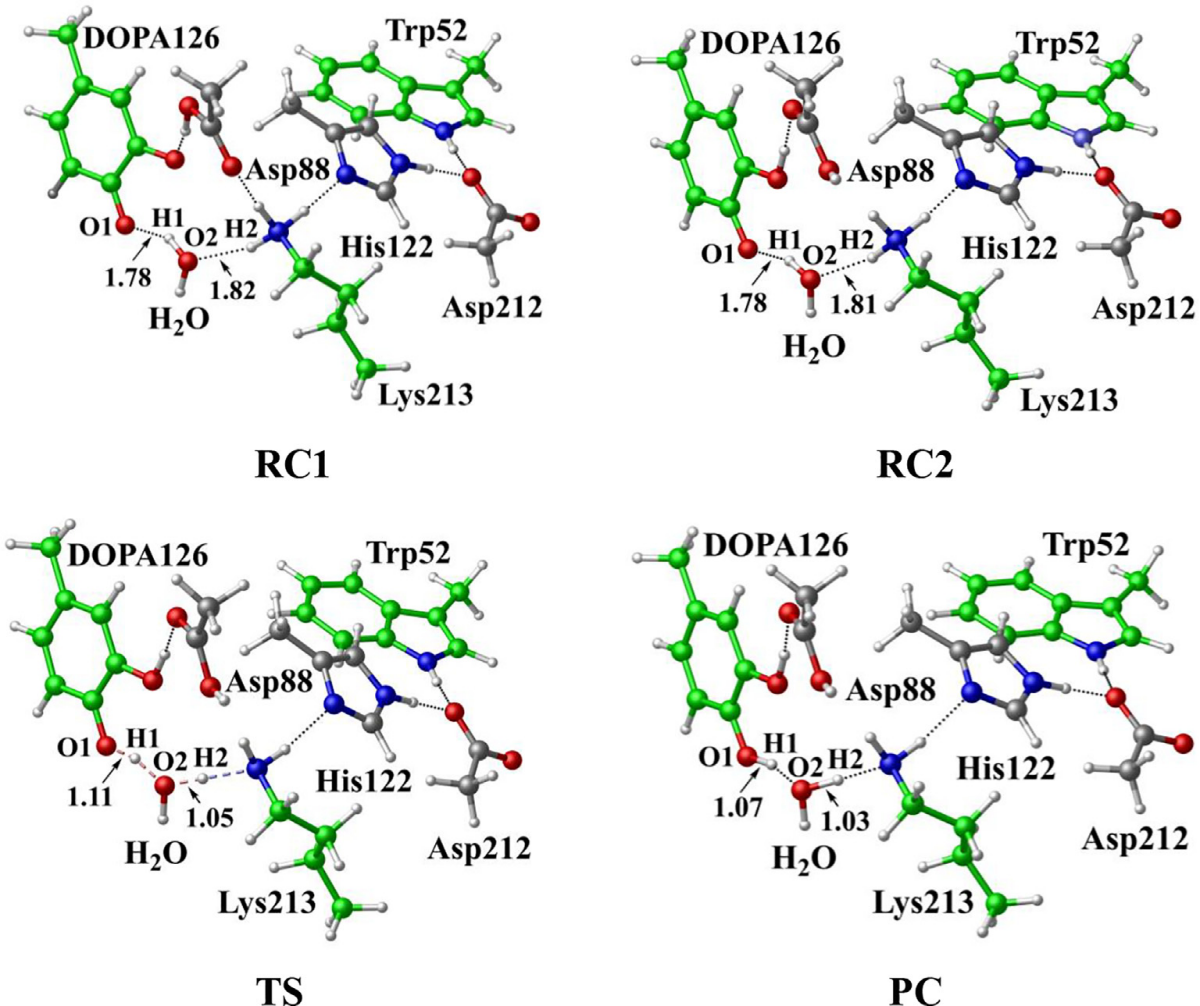
Structures and geometrical parameters for the DOPA radical transfer between DOPA126 and Trp52 in the protonated state of Asp88 (pathway B). For clarity, only six residues of the model are shown here. The atoms in the MM region which is linked to the QM region are replaced by hydrogens. Red, blue, and white represent oxygen, nitrogen, and hydrogen, respectively. For DOPA126, Lys213 and Trp52, the carbon atoms and the C–C bonds are shown in green. For other residues, the carbon atoms are shown in gray. The length is in Angstrom.

**Fig. 12:**
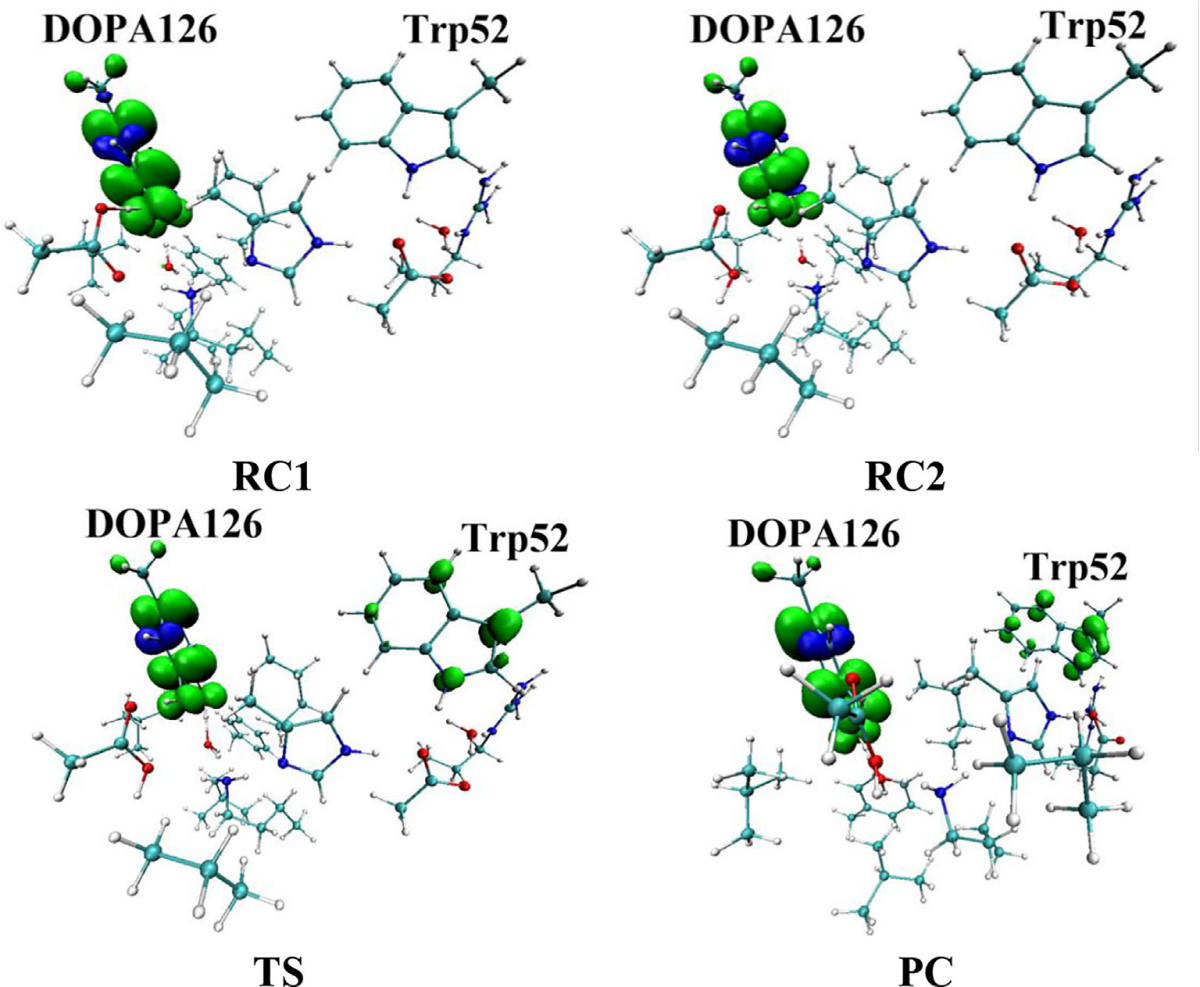
Spin density distributions for the DOPA radical transfer between DOPA126 and Trp52 in the protonated state of Asp88 (pathway B). Blue and green represent the negative and positive spin density, respectively.

**Fig. 13:**
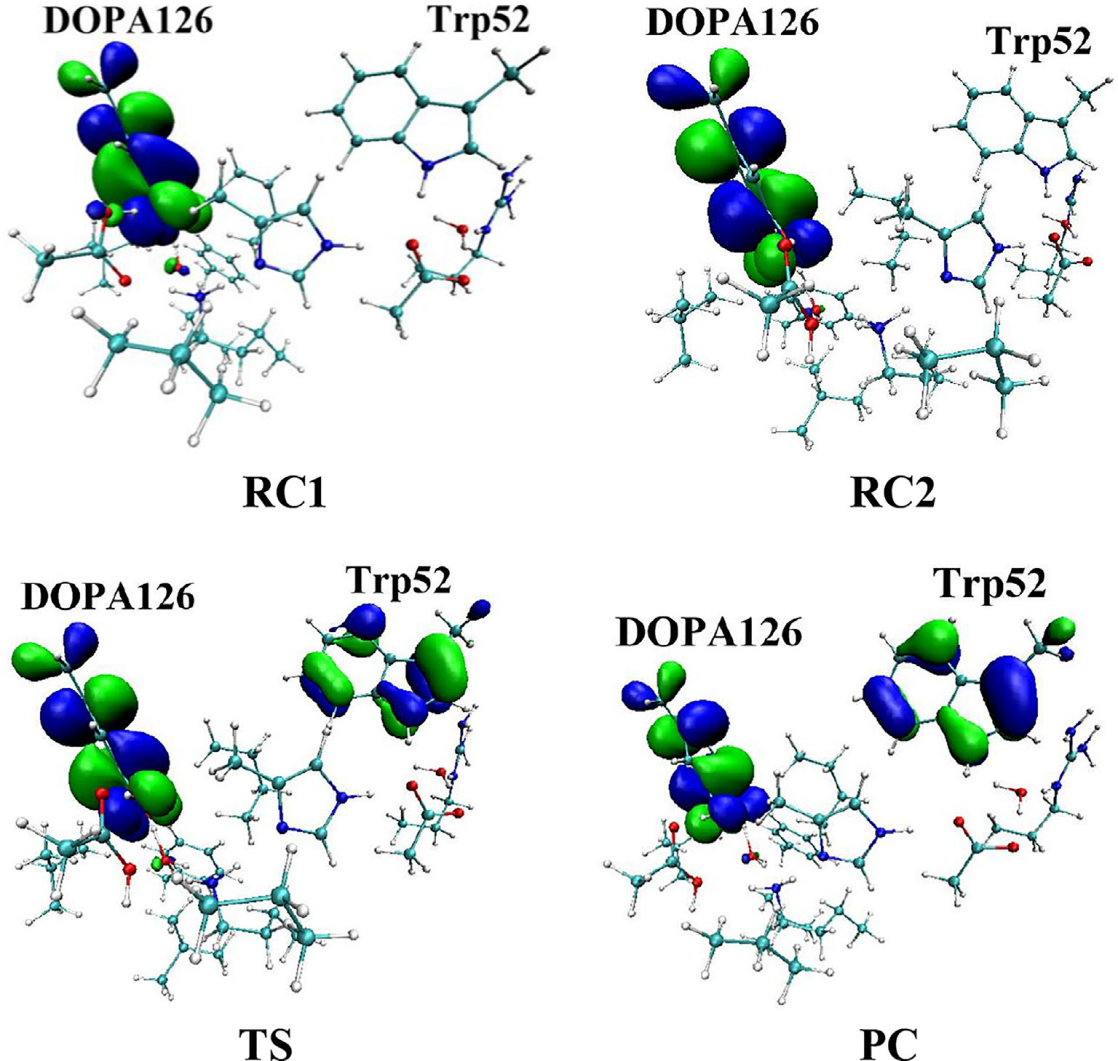
Spin natural orbitals distribution of unpaired single-electron for the DOPA radical transfer between DOPA126 and Trp52 in the protonated state of Asp88 (pathway B). Blue and green represent the negative and positive spin density, respectively.

Further calculations were carried out. The structures are illustrated in Fig. 11, including the reactant which has a neutral DOPA radical in the deprotonated state of Asp88 (DOPA126^•^-Trp52, RC1), the reactant in the protonated state of Asp88 (DOPA126^•^-Trp52, RC2), the transition state (TS), and the product complex (DOPA126-Trp52^+•^, PC). In RC2, there are two intermolecular hydrogen bonds between DOPA126 and Lys213. The optimized distances of O1-H1 and O2-H2 are 1.78 Å and 1.81 Å, respectively. About 98% of the unpaired single-electron is distributed on DOPA126, which has a spin density of 1.0 (Table 2, Fig. 12, Fig. 13). There is almost no spin density resides on the other residues. The charge analysis (Table 2) shows that the total charge of Trp52 is −0.10, indicating that Trp52 is initially electronegative. As the radical transfer is initiated, the atom H1 gradually moves toward O1, and H2 gradually approaches to O2. This step undergoes a transition state (TS). From RC2 to TS, the O1-H1 distance changes from 1.78 Å to 1.11 Å, and the O2-H2 distance changes from 1.81 Å to 1.05 Å. In TS, about 14% of the unpaired single-electron is transferred to Trp52 (Table 2, Fig. 12, Fig. 13). As the reaction proceeds, the O1-H1 and O2-H2 distances are gradually shortened. In PC, the O1-H1 and O2-H2 bonds are formed simultaneously. The lengths of the O1-H1 and O2-H2 bonds are 1.07 Å and 1.03 Å, respectively. As the reaction going from RC2 to PC, the charge of Trp52 changes from −0.01 to 0.06, and the spin density changes from 0.00 to 0.19 (Table 2, Fig. 12). About 19% of the unpaired single-electron is distributed on Trp52 in PC (Table 2, Fig. 13), revealing that the cationic tryptophan radical Trp52^+•^ is formed, although most of the unpaired single-electron (79%) is still located on DOPA126. The unpaired spin density mainly resides on the aromatic ring of DOPA126 in PC, demonstrating that the electron transfer between these two residues is complicated. The changes of protein environment and conformation may be needed in the transfer process. Our calculations help to understand radical transfer mechanism, and address the concern from the articles that how the DOPA radical is transferred from the radical trap.

**Table 2:** Atomic dipole corrected hirshfeld (ADCH) atomic charges (unit: C), spin densities and spin natural orbitals (SNO) distribution of unpaired single-electron for the DOPA radical transfer between DOPA126 and Trp52 in the protonated state of Asp88 (pathway B).

		DOPA126	Trp52
ADCH charge	RC1	−0.50	−0.10
	RC2	0.10	−0.10
	TS	0.50	0.02
	PC	0.49	0.06
Spin density	RC1	0.99	0.00
	RC2	1.00	0.00
	TS	0.86	0.14
	PC	0.80	0.19
SNO distribution	RC1	97%	0
	RC2	98%	0
	TS	84%	14%
	PC	79%	19%

From RC2 to PC, the reaction is endothermic. The forward and backward energy barriers are 15.2 and 1.0 kcal/mol (Fig. 9), respectively, and the calculated rate constants are 1.17 × 10^3^ and 2.89 × 10^13^ s^−1^, respectively. The forward rate constant obtained is in agreement with previous study [74]. The backward energy barrier is slightly endothermic (1.0 kcal/mol), which means that the transfer rate is very fast.

**Scheme 5:**
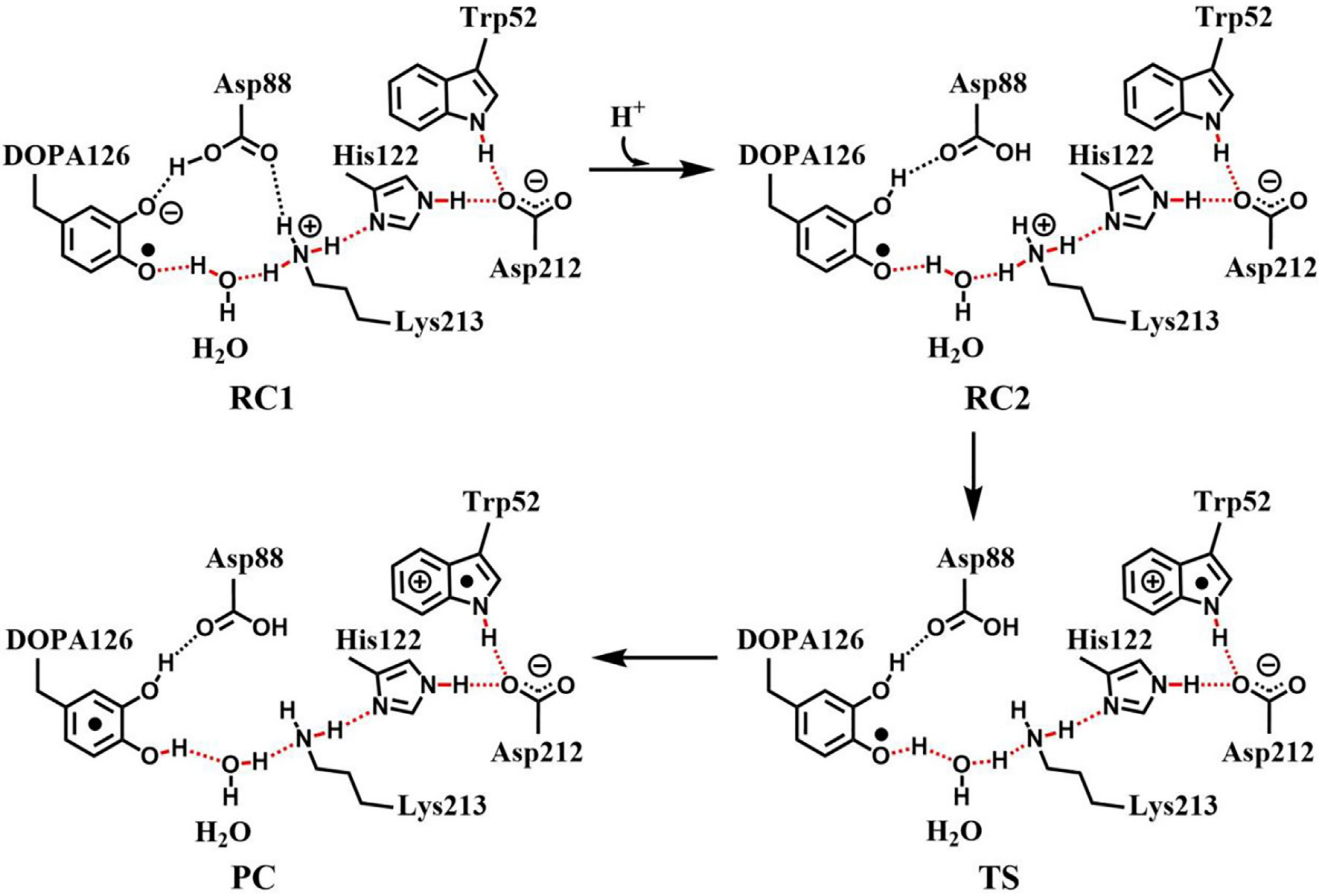
The pathway for the DOPA radical transfer between DOPA126 and Trp52 based on DFT simulation (pathway B). The line in red represents the radical transfer chain.

**Fig. 14:**
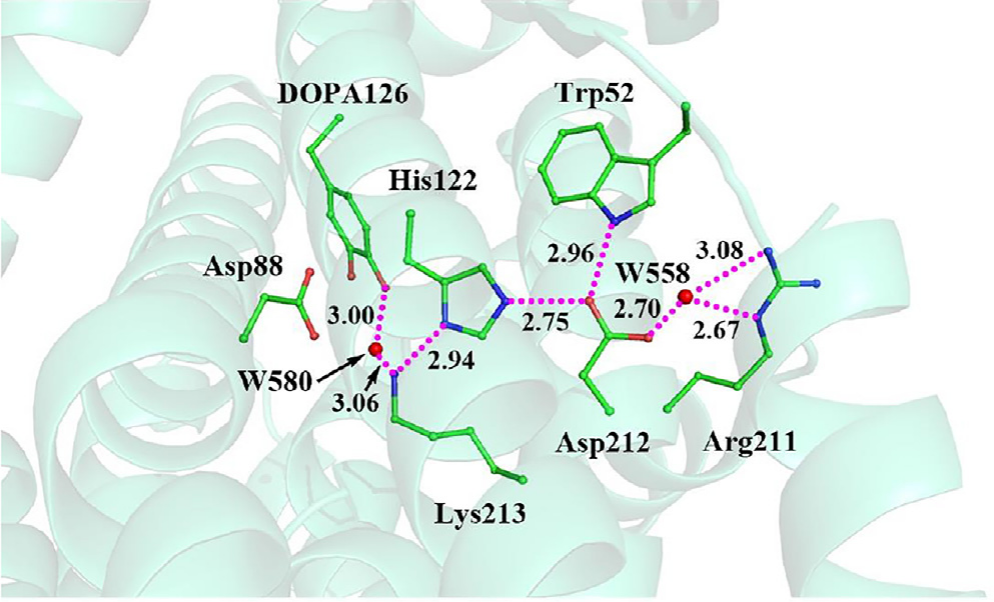
Radical transfer chain in the active *MfR2* (PDB: 6GP2). Green, red, and blue represent carbon, oxygen, and nitrogen, respectively, and water is represented by red sphere. The dotted line in pink represents radical transfer chain.

Scheme 5 illustrates the radical transfer between DOPA126 and Trp52. According to the mechanism of the radical transfer between DOPA126 and Trp52 and the hydrogen bonds formed, the residues along the radical transfer chain are identified, DOPA126 ↔ H_2_O ↔ Lys213 ↔ His122 ↔ Asp212 ↔ Trp52 ↔ Arg211 ↔ Tyr356 (Fig. 14) (Tyr356 was not resolved in the crystal structure).

The ε-amino group of Lys213, not only as a bridge for electron transfer but also as a proton donor, provides a proton to the phenoxyl radical (C-O•) of DOPA126 to form the phenol group (C-OH). Lys213 is the key residue in the radical transfer chain between DOPA126 and Trp52. Between the side chains of DOPA126 and Lys213, there is a water molecule (W580). The water molecule forms hydrogen bonds with both DOPA126 and Lys213. Thus, an electron and proton channel is formed. It is a water mediated double-proton-coupled electron transfer (dPCET), that occurs between the side chains of DOPA126 and Lys213. The dPCET mechanism indicates that protons can be transferred between the side chains of DOPA126 and Lys213. Previous studies have shown that, the existence of water molecule between the side chains can decrease the barrier height of electron tunneling and facilitate the electron transfer [53], [75], [76].

### 3.3 Mechanism of the DOPA radical regeneration

#### 3.3.1 Superoxide (O_2_^•−^) as the oxidant

**Fig. 15:**
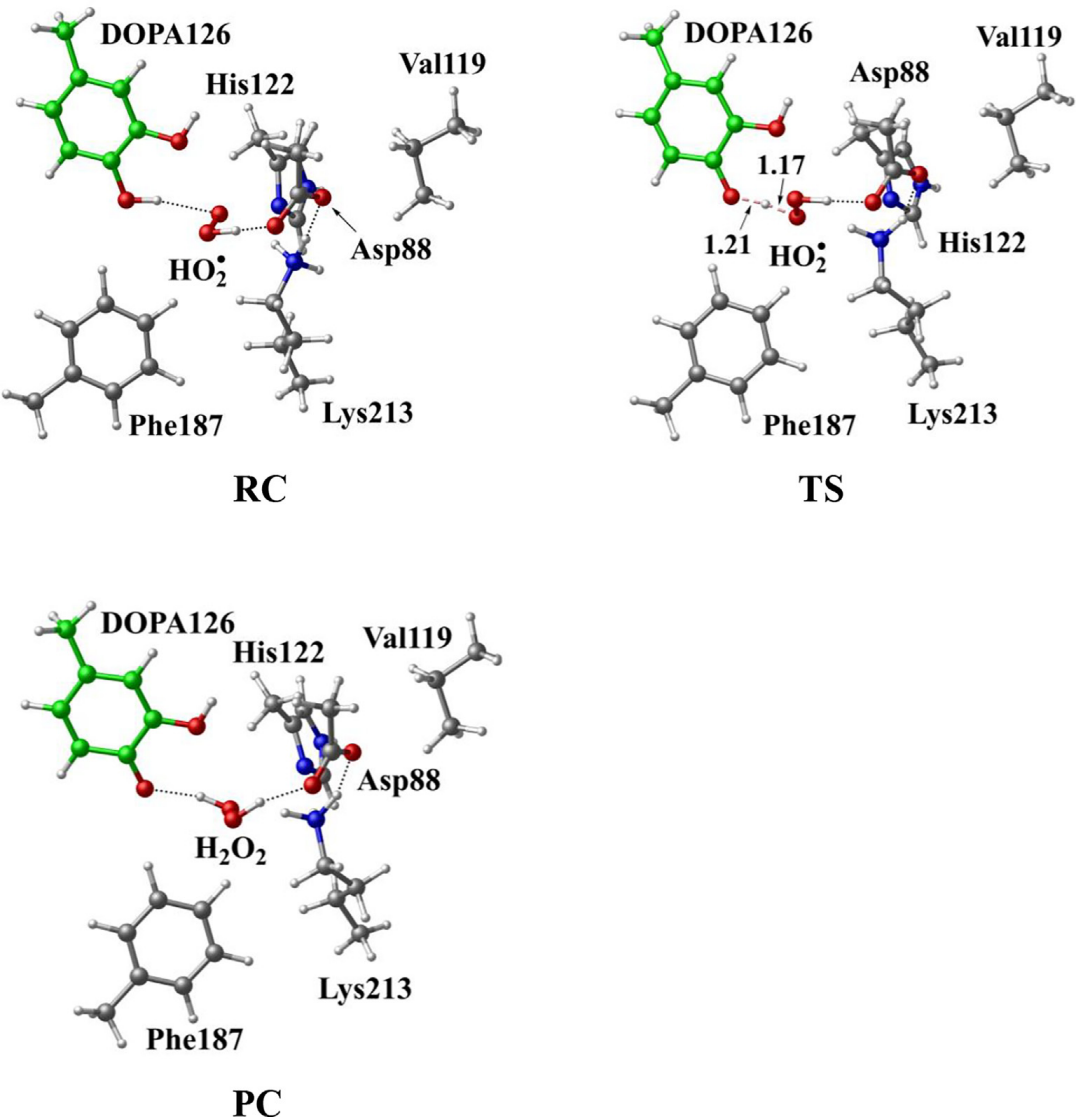
Structures for the DOPA radical regeneration using O_2_^•−^ as the oxidant in the protonated state of Asp88 (pathway C). For clarity, only six residues of the model are shown here. The atoms in the MM region which is linked to the QM region are replaced by hydrogens. Red, blue, and white represent oxygen, nitrogen, and hydrogen, respectively. For DOPA126, the carbon atoms and the C–C bonds are shown in green. For other residues, the carbon atoms are shown in gray. The dotted line in black represents hydrogen bond. The length in Angstrom.

**Fig. 16:**
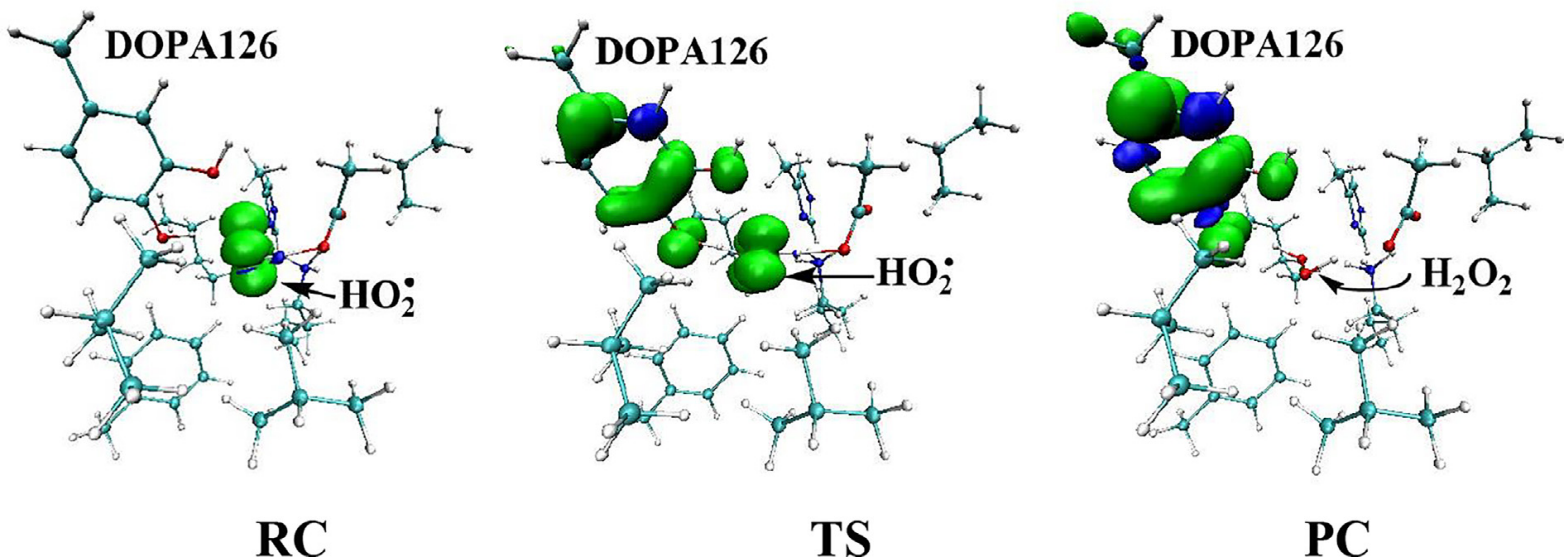
Spin density distributions for the DOPA radical regeneration using O_2_^•−^ as the oxidant in the protonated state of Asp88 (pathway C). Blue and green represent the negative and positive spin density, respectively.

**Fig. 17:**
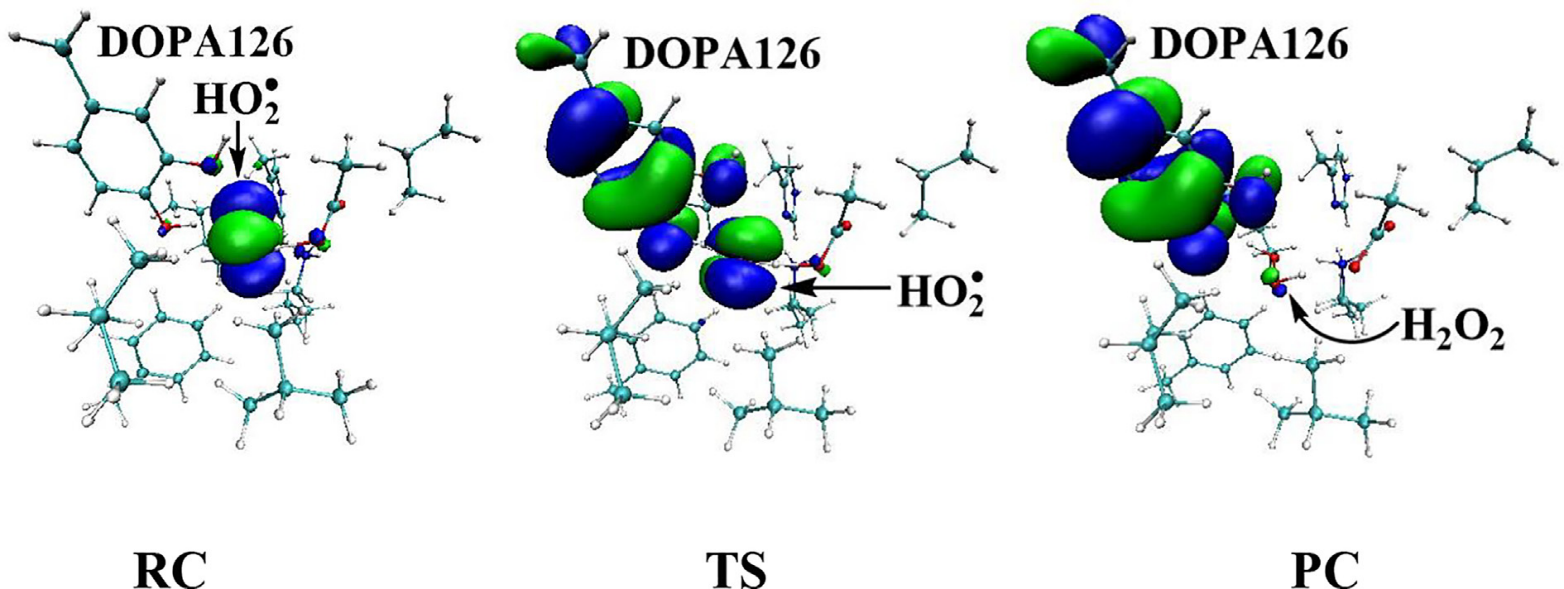
Spin natural orbitals distribution of unpaired single-electron for the DOPA radical regeneration using O_2_^•−^ as the oxidant in the protonated state of Asp88 (pathway C). Blue and green represent the negative and positive spin density, respectively.

**Scheme 6:**
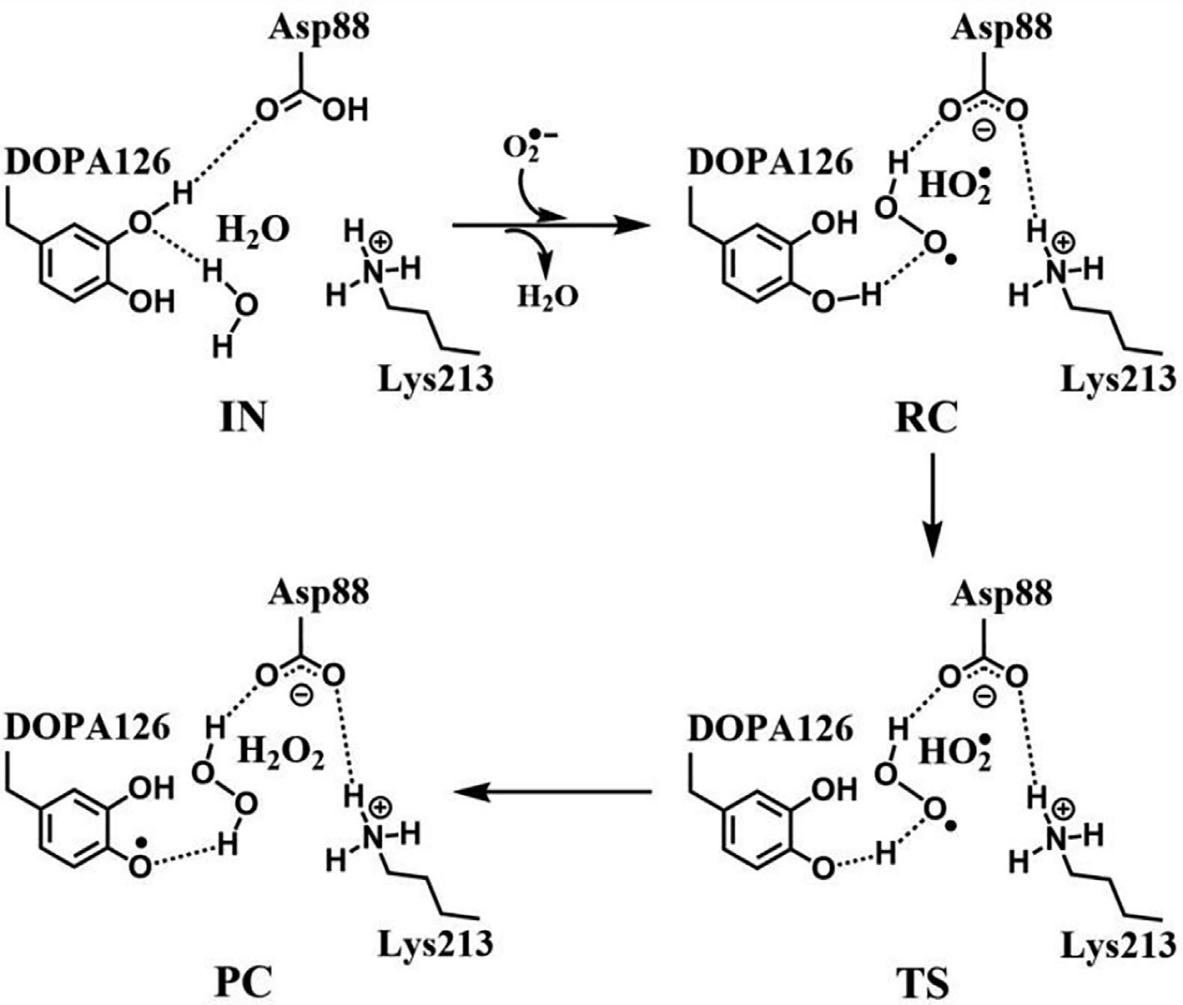
The pathway for the DOPA radical regeneration using O_2_^•−^ as the oxidant in the protonated state of Asp88 based on DFT simulation (pathway C).

The DFT calculations have shown that the Asp88 being in the protonation state is the prerequisite for the DOPA radical generation and transfer. For the regeneration of the DOPA radical, we simulated the oxidation by O_2_^•−^ when Asp88 is in the protonated state. The optimized structures of the key steps of the process are illustrated in Fig. 15. The oxidant O_2_^•−^ occupies the position of the water molecule when it enters the active site. In RC, the proton in the carboxyl group of the side-chain of Asp88 is transferred to O_2_^•−^ to form HO_2_^•^ due to the strong electronegativity of O_2_^•−^. As the reaction going from RC to PC, the phenol proton of DOPA126 is transferred to HO_2_^•^, the free radical is transferred concomitantly from HO_2_^•^ to DOPA126. HO_2_^•^ is protonated to form a hydrogen peroxide molecule in PC. The reaction undergoes a transition state (TS). Fig. 16, Fig. 17 show the transfer of the unpaired single-electron from HO_2_^•^ to DOPA126. The calculated energy barrier is 4.0 kcal/mol, and the reaction free energy is −5.2 kcal/mol (pathway C in Fig. 9), indicating that the reaction is exothermic. Hence, the regeneration reaction is thermodynamically favorable from the energy point of view. The reaction mechanism is illustrated in Scheme 6.

#### 3.3.2 Hydroperoxyl radical (HO_2_^•^) as the oxidant

**Fig. 18:**
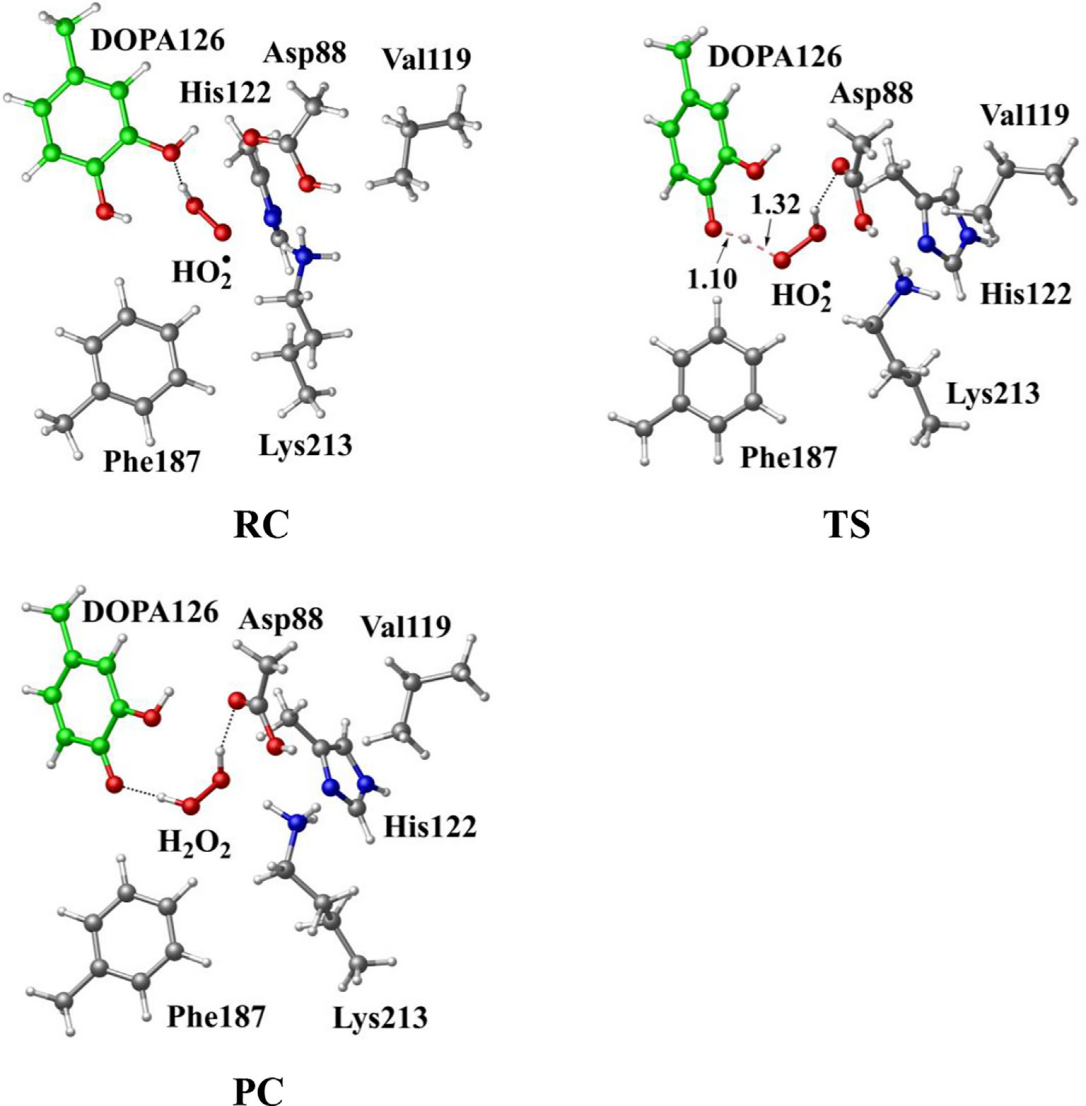
Structures for the DOPA radical regeneration using HO_2_^•^ as the oxidant in the protonated state of Asp88 (pathway D). For clarity, only six residues of the model are shown here. The atoms in the MM region which is linked to the QM region are replaced by hydrogens. Red, blue, and white represent oxygen, nitrogen, and hydrogen, respectively. For DOPA126, the carbon atoms and the C–C bonds are shown in green. For other residues, the carbon atoms are shown in gray. The dotted line in black represents hydrogen bond. The length is in Angstrom.

**Fig. 19:**
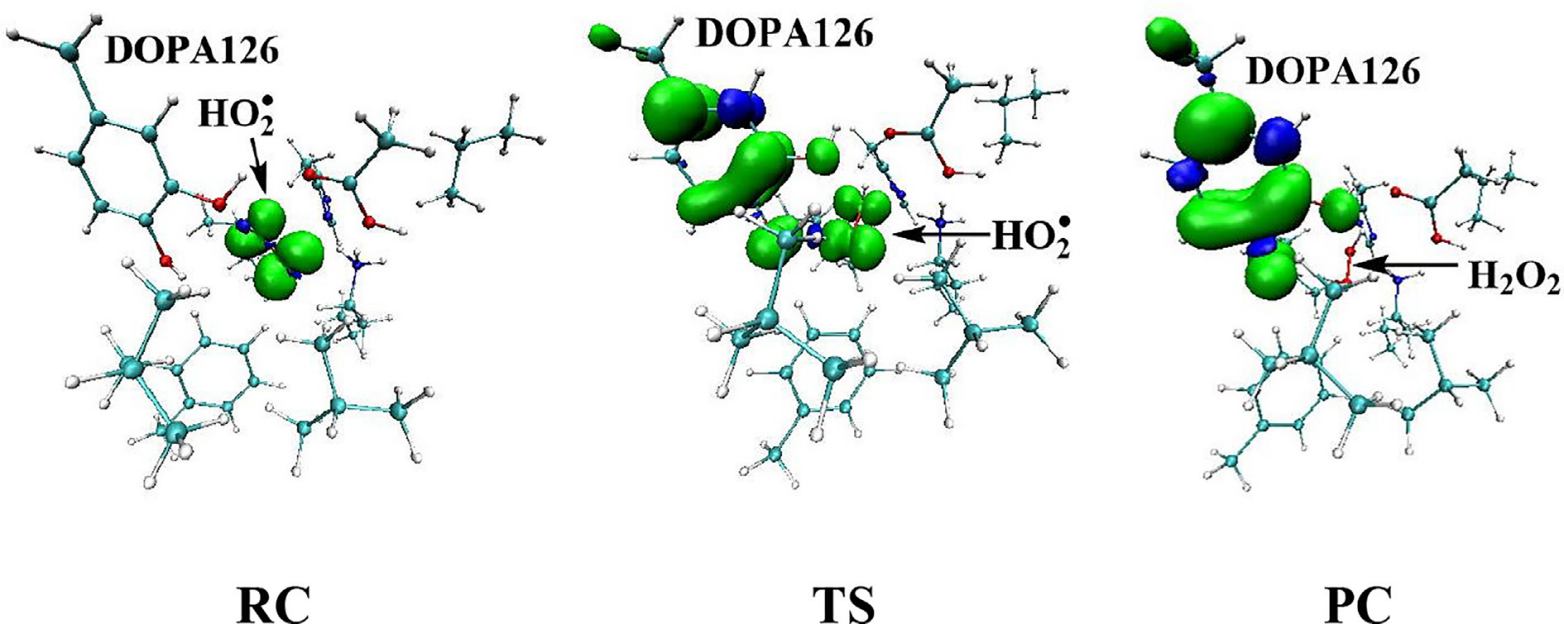
Spin density distributions for the DOPA radical regeneration using HO_2_^•^ as the oxidant in the protonated state of Asp88 (pathway D). Blue and green represent the negative and positive spin density, respectively.

**Fig. 20:**
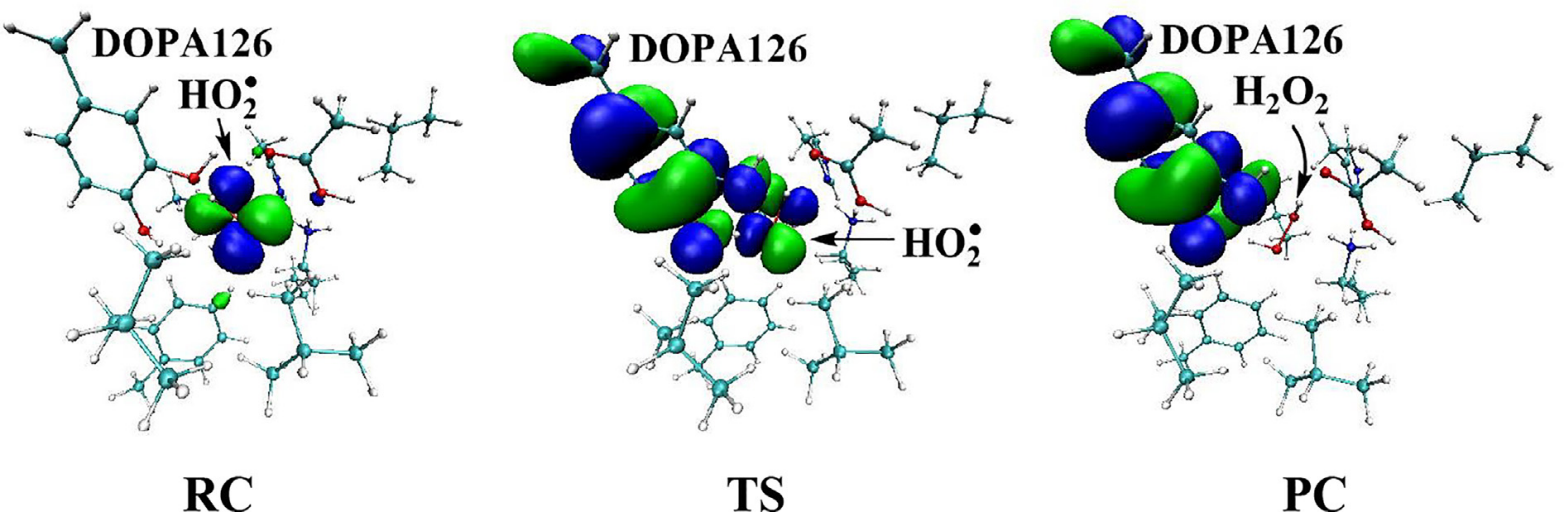
Spin natural orbitals distribution of unpaired single-electron for the DOPA radical regeneration using HO_2_^•^ as the oxidant in the protonated state of Asp88 (pathway D). Blue and green represent the negative and positive spin density, respectively.

**Scheme 7:**
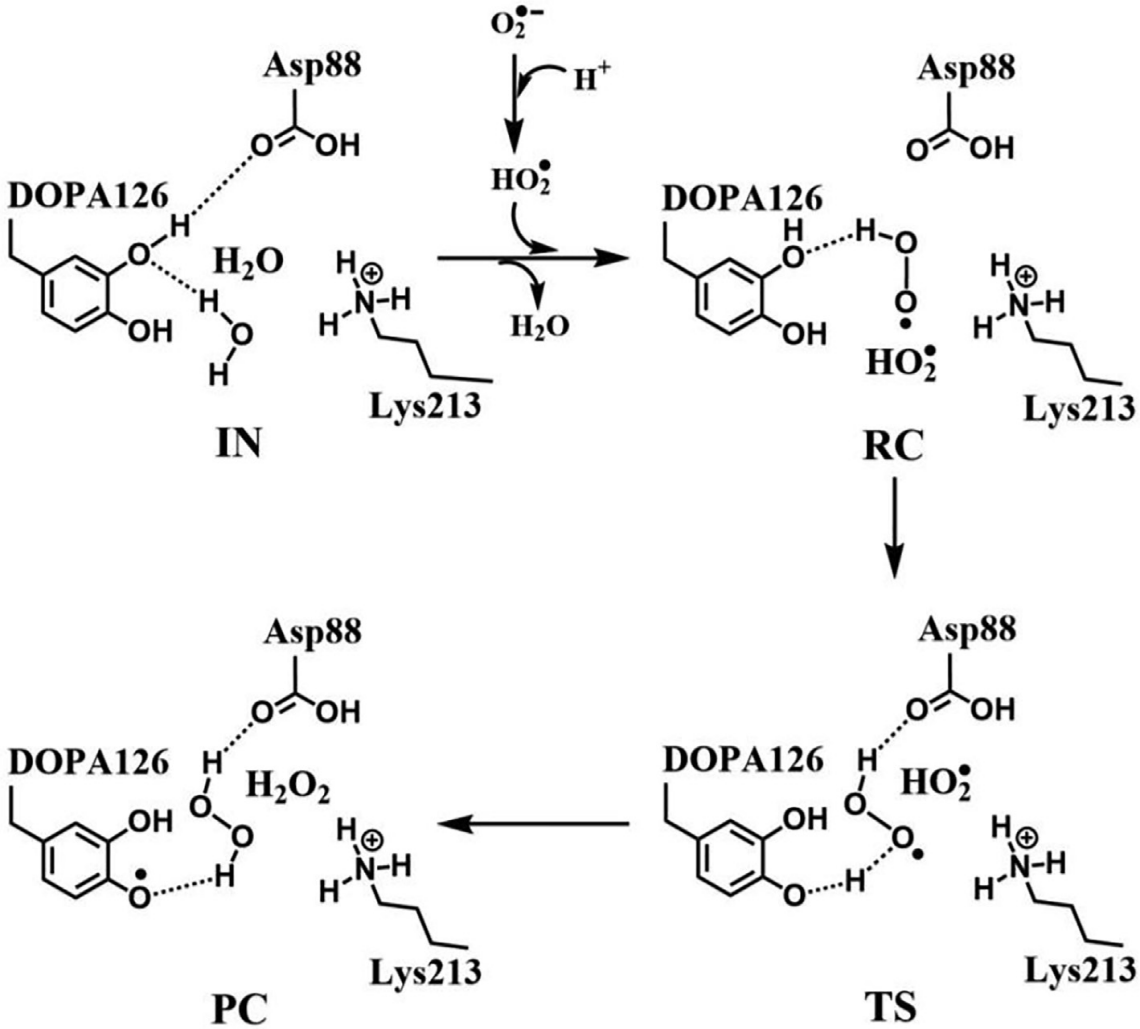
The pathway for the DOPA radical regeneration using HO_2_^•^ as the oxidant in the protonated state of Asp88 based on DFT simulation (pathway D).

DFT calculations also have been carried out using hydroperoxyl radical (HO_2_^•^) as the oxidant, and the optimized structures of the key steps of the process are illustrated in Fig. 18. In RC, HO_2_^•^ is hydrogen bonded to the C3′ hydroxyl group of DOPA126. As the reaction going from RC to PC, the phenol proton of DOPA126 is transferred to HO_2_^•^, the free radical is transferred concomitantly from HO_2_^•^ to DOPA126. HO_2_^•^ is protonated to form a hydrogen peroxide molecule in PC. The reaction also undergoes a transition state (TS). Fig. 19, Fig. 20 confirm the transfer of the unpaired single-electron from HO_2_^•^ to DOPA126. The calculated energy barrier is 6.7 kcal/mol, and the reaction free energy is −12.7 kcal/mol (pathway D in Fig. 9). The reaction is exothermic and the regeneration reaction is thermodynamically favorable. The reaction mechanism is illustrated in Scheme 7.

## 4 Conclusions

For class Ie RNR, the mechanisms of the covalent modification of Tyr126 to the DOPA radical and the radical transfer and regeneration have been studied based on DFT calculations. As a new class of RNR enzyme, class Ie exhibits different mechanisms of radical generation and transfer. The DFT calculations have showed that O_2_^•−^ cannot directly oxidize Tyr126 to form the DOPA radical in class Ie. It must be protonated to HO_2_^•^ prior to the oxidation. The DFT calculations suggest that the covalent modification of Tyr126 and the DOPA radical generation can be carried out with no involvement of metal cofactors. The DFT study reveals the steps of bond formation and bond breaking upon the oxidation of Tyr126 by HO_2_^•^. This work supports that metal environment and amount may not have an effect on the survival of pathogens containing class Ie [25], [26]. The protonation of Asp88 is the prerequisite for the generation and transfer of the DOPA radical in class Ie. The protonation state of Asp88 can be regulated by the change of protein microenvironment, which is induced by the protein aggregation and separation of the R2 subunit with flavoprotein NrdI or the R1 subunit. The ε-amino group of Lys213 plays an important role in the DOPA radical generation and transfer. Once the radical is quenched, it can be regenerated via the oxidations by superoxide O_2_^•−^ and hydroperoxyl radical HO_2_^•^.

## Declaration of Competing Interest

The authors declare that they have no known competing financial interests or personal relationships that could have appeared to influence the work reported in this paper.
