# Supplementary material for: Mechanism of DOPA radical generation and transfer in metal-free class Ie ribonucleotide reductase based on density functional theory
Source: Comput Struct Biotechnol J. 2022 Mar 2;20:1111–31. doi: 10.1016/j.csbj.2022.02.027 (PMC8902622; doi:10.1016/j.csbj.2022.02.027)
Supplement: Supplementary data 1 [file mmc1.doc]

**Supplementary Information**

Mechanism of DOPA Radical Generation and Transfer in Metal-Free Class Ie Ribonucleotide Reductase Based on Density Functional Theory

Jinxin Zou, Yao Chen, Wei Feng[[1]](#footnote-2)*

Department of Biological Engineering, Beijing University of Chemical Technology, Beijing, 100029, China


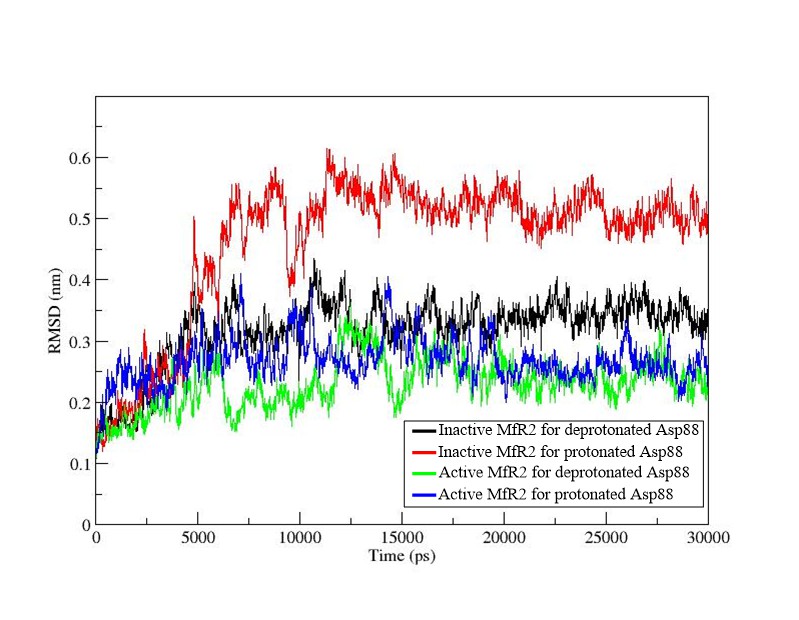


**Figure S1.** RMSD changes in skeleton atoms of protein along the MD trajectories for the inactive and active MfR2.


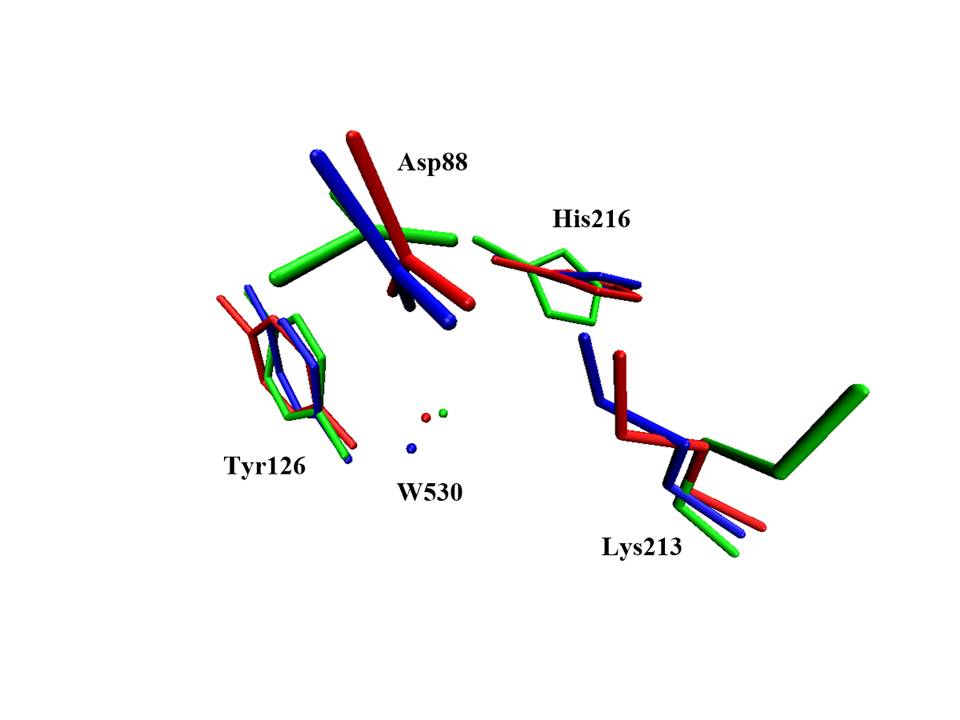

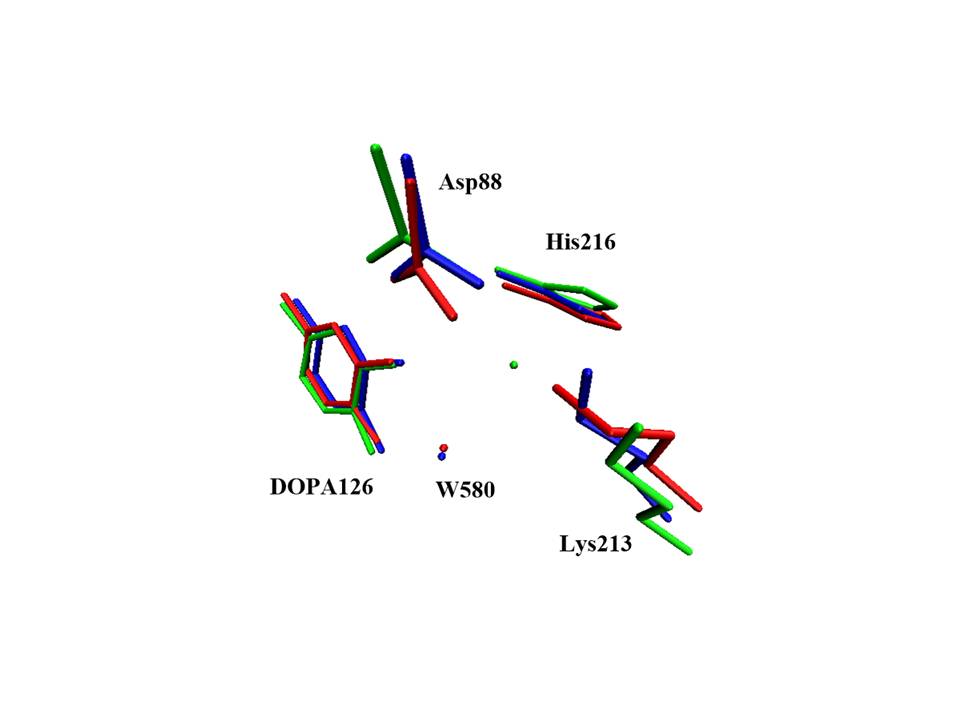


**a b**

**Figure S2.** Overlay of the initial crystal structures and the structures after the MD simulations (30 ns) for the Ie RNR-R2 active site. (a) Structure of active site in the inactive MfR2 (PDB: 6GP3). (b) Structure of active site in the active MfR2 (PDB: 6GP2). Red, blue and Green represent the initial crystal structure, structures of state 1 (Asp88 is in deprotonated state) and state 2 (Asp88 is in protonated state) after 30 ns of running, respectively. Compared to the initial crystal structure, the deprotonated state in the active site maintains stable after 30 ns of running both in the inactive and active MfR2. The root mean square deviations (RMSD) for states 1 and 2 in the inactive MfR2 are 0.49 Å and 1.11 Å, respectively, and in the active MfR2 are 0.51 Å and 0.96 Å, respectively.

**Table S1.** Absolute (Hartree) and relative (kcal/mol) energies and energy corrections for the DOPA radical generation using HO_2_^•^ as the oxidant in the protonated state of Asp88 (Pathway A).

|  | **E**  **(Hartree)** | **△E_ZPE_**  **(Hartree)** | **E+△E_ZPE_ (Hartree)** | **△E**  **(kcal/mol)** |
| --- | --- | --- | --- | --- |
| **RC** | -2073.643658 | 9.152583 | -2064.491075 | 0.0 |
| **TS1** | -2073.614661 | 9.148342 | -2064.466319 | 15.5 |
| **IM1** | -2073.628711 | 9.148937 | -2064.479774 | 7.1 |
| **TS2** | -2073.614418 | 9.151412 | -2064.463006 | 17.6 |
| **IM2** | -2073.697064 | 9.148975 | -2064.548089 | -35.8 |
| **TS3** | -2073.665164 | 9.146143 | -2064.519021 | -17.5 |
| **PC** | -2073.770036 | 9.152768 | -2064.617268 | -79.2 |

**Table S2.** Absolute (Hartree) and relative (kcal/mol) energies and energy corrections for the DOPA radical transfer between DOPA126 and Trp52 in the protonated state of Asp88 (Pathway B).

|  | **E**  **(Hartree)** | **△E_ZPE_**  **(Hartree)** | **E+△E_ZPE_ (Hartree)** | **△E**  **(kcal/mol)** |
| --- | --- | --- | --- | --- |
| **RC2** | -3106.109156 | 50.499340 | -3055.609816 | 0.0 |
| **TS** | -3106.079100 | 50.493490 | -3055.585610 | 15.2 |
| **PC** | -3106.082581 | 50.495374 | -3055.587207 | 14.2 |

**Table S3.** Absolute (Hartree) and relative (kcal/mol) energies and energy corrections for the DOPA radical regeneration using O_2_^•-^ as the oxidant in the protonated state of Asp88 (Pathway C).

|  | **E**  **(Hartree)** | **△E_ZPE_**  **(Hartree)** | **E+△E_ZPE_ (Hartree)** | **△E**  **(kcal/mol)** |
| --- | --- | --- | --- | --- |
| **RC** | -2148.1403895 | 9.152736 | -2138.987654 | 0.0 |
| **TS** | -2148.1299768 | 9.148739 | -2138.981245 | 4.0 |
| **PC** | -2148.1506808 | 9.154784 | -2138.995897 | -5.2 |

**Table S4.** Absolute (Hartree) and relative (kcal/mol) energies and energy corrections for the DOPA radical regeneration using HO_2_^•^ as the oxidant in the protonated state of Asp88 (Pathway D).

|  | **E**  **(Hartree)** | **△E_ZPE_**  **(Hartree)** | **E+△E_ZPE_ (Hartree)** | **△E**  **(kcal/mol)** |
| --- | --- | --- | --- | --- |
| **RC** | -2148.5446234 | 9.162386 | -2139.382242 | 0.0 |
| **TS** | -2148.5282236 | 9.156723 | -2139.371501 | 6.7 |
| **PC** | -2148.5653189 | 9.162806 | -2139.402513 | -12.7 |

**Cartesian coordinates for the DOPA radical generation using HO_2_^•^ as the oxidant in the protonated state of Asp88 (Pathway A).**

| **IN1** | | | | | | | | | |
| --- | --- | --- | --- | --- | --- | --- | --- | --- | --- |
| 1 | C | 50.83627900 | 66.82764400 | 55.89152400 | 63 | H | 50.76239500 | 60.96318500 | 58.92788300 |
| 2 | H | 51.25244300 | 66.61209100 | 56.89198200 | 64 | C | 51.55660100 | 63.53804400 | 58.65032800 |
| 3 | H | 50.60751000 | 67.90495500 | 55.89811800 | 65 | H | 51.59887800 | 64.46862400 | 58.06275400 |
| 4 | C | 51.90819800 | 66.52067200 | 54.82886400 | 66 | H | 52.16122400 | 63.67042000 | 59.56396200 |
| 5 | H | 51.50932900 | 66.77506000 | 53.83464000 | 67 | H | 50.51863200 | 63.39992700 | 58.98654400 |
| 6 | C | 53.13926300 | 67.40768800 | 55.05708200 | 68 | C | 58.73479400 | 66.06088200 | 55.68626600 |
| 7 | H | 53.61909600 | 67.17340100 | 56.02031500 | 69 | H | 59.57275100 | 66.64705300 | 56.10952700 |
| 8 | H | 52.86478000 | 68.47341500 | 55.08414900 | 70 | H | 59.17593600 | 65.09899400 | 55.38275500 |
| 9 | H | 53.88998100 | 67.26826300 | 54.26268100 | 71 | C | 57.70739900 | 65.83128400 | 56.77169800 |
| 10 | C | 52.29895400 | 65.03811700 | 54.78581400 | 72 | C | 56.61942600 | 66.69132000 | 56.99092500 |
| 11 | H | 51.45317200 | 64.38777000 | 54.50893300 | 73 | H | 56.45281600 | 67.54781500 | 56.33324100 |
| 12 | H | 52.67713100 | 64.70303900 | 55.76391900 | 74 | C | 55.72898700 | 66.48125900 | 58.04747700 |
| 13 | H | 53.09903200 | 64.85200600 | 54.05268600 | 75 | H | 54.90171800 | 67.17715900 | 58.19993800 |
| 14 | C | 47.86436400 | 65.47437300 | 61.43529300 | 76 | C | 55.89792400 | 65.38456700 | 58.89760200 |
| 15 | H | 47.65013800 | 65.87159700 | 62.44173500 | 77 | H | 55.19409900 | 65.21903200 | 59.71612100 |
| 16 | H | 47.46628000 | 66.19761800 | 60.71109800 | 78 | C | 57.86500200 | 64.74204100 | 57.64355700 |
| 17 | C | 49.38712700 | 65.40544900 | 61.33525500 | 79 | H | 58.71003000 | 64.06402100 | 57.49973100 |
| 18 | O | 49.95382500 | 64.32037200 | 61.68606400 | 80 | C | 56.96796100 | 64.51060400 | 58.68811100 |
| 19 | O | 49.98529700 | 66.44759800 | 60.98236200 | 81 | H | 57.10654500 | 63.64139300 | 59.33786300 |
| 20 | C | 48.71743900 | 63.99350500 | 66.20396800 | 82 | C | 55.81733500 | 68.39052800 | 62.83662400 |
| 21 | H | 47.98867800 | 63.77190100 | 67.00428300 | 83 | H | 56.60560300 | 68.64828300 | 62.11186700 |
| 22 | C | 48.04409300 | 63.73541300 | 64.85993500 | 84 | C | 55.16837000 | 67.07656000 | 62.38127900 |
| 23 | H | 48.74657100 | 63.81293400 | 64.01561700 | 85 | H | 54.51942800 | 66.65790900 | 63.16724400 |
| 24 | H | 47.20993600 | 64.42074600 | 64.65951700 | 86 | H | 55.91615000 | 66.31276800 | 62.11254300 |
| 25 | H | 47.62952300 | 62.71765600 | 64.86723500 | 87 | H | 54.53775100 | 67.21898900 | 61.49312100 |
| 26 | C | 49.91395400 | 63.04097900 | 66.34041000 | 88 | C | 54.79124100 | 69.54226900 | 62.83981700 |
| 27 | H | 50.44654600 | 63.11689600 | 67.29671000 | 89 | H | 55.18701200 | 70.41855000 | 63.37852900 |
| 28 | H | 50.65480100 | 63.21971500 | 65.54310800 | 90 | H | 53.89601000 | 69.22391300 | 63.40046600 |
| 29 | H | 49.57661400 | 61.99703700 | 66.23830600 | 91 | C | 54.38900000 | 69.99662700 | 61.43645100 |
| 30 | C | 51.24560200 | 67.92093300 | 63.75461000 | 92 | H | 54.01815200 | 69.17765500 | 60.80516400 |
| 31 | H | 51.78510600 | 67.87459300 | 62.79329800 | 93 | H | 55.24544500 | 70.44860200 | 60.91411800 |
| 32 | H | 50.38371800 | 67.25597700 | 63.61020800 | 94 | H | 53.59594100 | 70.75646600 | 61.46980000 |
| 33 | N | 52.43883100 | 66.03399800 | 64.90338100 | 95 | C | 56.90145600 | 62.32399800 | 63.71397800 |
| 34 | C | 52.11797600 | 67.38021900 | 64.84404200 | 96 | H | 56.82131100 | 61.35195600 | 63.19222500 |
| 35 | C | 53.20905700 | 65.86991600 | 65.96147000 | 97 | H | 57.92753200 | 62.67281000 | 63.51452500 |
| 36 | H | 53.63221500 | 64.92717600 | 66.30652600 | 98 | C | 55.90472800 | 63.29693500 | 63.07536000 |
| 37 | N | 53.41623300 | 67.04819300 | 66.59426300 | 99 | H | 56.05660600 | 63.23864100 | 61.98265400 |
| 38 | C | 52.73434300 | 68.02031100 | 65.89759700 | 100 | H | 56.14246900 | 64.33717100 | 63.33925400 |
| 39 | H | 52.69270200 | 69.05079200 | 66.23521600 | 101 | C | 54.42133800 | 63.05509300 | 63.38147800 |
| 40 | C | 49.30169600 | 72.65948300 | 58.02289700 | 102 | H | 54.20574400 | 63.37938700 | 64.41505500 |
| 41 | H | 49.09634500 | 72.90867800 | 56.96683000 | 103 | H | 54.19012100 | 61.97539500 | 63.34636200 |
| 42 | H | 48.33460200 | 72.37459100 | 58.46377900 | 104 | C | 53.48254300 | 63.79028000 | 62.41612600 |
| 43 | C | 50.26555400 | 71.50375700 | 58.12547000 | 105 | H | 53.40387900 | 63.23870800 | 61.46701200 |
| 44 | C | 51.55914400 | 71.56579000 | 57.57936200 | 106 | H | 53.84052800 | 64.79864200 | 62.17610000 |
| 45 | H | 51.87728200 | 72.46354700 | 57.04038600 | 107 | N | 52.11961200 | 63.94392000 | 62.98249300 |
| 46 | C | 52.45830800 | 70.51249000 | 57.72370700 | 108 | H | 52.13562800 | 64.68092300 | 63.73988900 |
| 47 | H | 53.46380100 | 70.56945800 | 57.30312700 | 109 | H | 51.82263000 | 63.05117000 | 63.40741700 |
| 48 | C | 52.09239200 | 69.35723700 | 58.43605000 | 110 | H | 51.29894500 | 64.20956900 | 62.27782800 |
| 49 | O | 53.01258700 | 68.37580000 | 58.57493800 | 111 | O | 52.47095300 | 66.36979400 | 60.25591400 |
| 50 | H | 52.71746300 | 67.67700900 | 59.21910300 | 112 | H | 51.51296300 | 66.39150200 | 60.58905000 |
| 51 | C | 49.91152700 | 70.33468300 | 58.80879600 | 113 | H | 52.47941600 | 65.61743700 | 59.64807900 |
| 52 | H | 48.91218800 | 70.25864200 | 59.24374000 | 114 | H | 53.96656400 | 67.18862700 | 67.43687000 |
| 53 | C | 50.79751400 | 69.26749800 | 58.97032900 | 115 | H | 49.62385645 | 73.56317469 | 58.49666274 |
| 54 | H | 50.48017500 | 68.37947000 | 59.52234000 | 116 | H | 56.83715874 | 62.09871867 | 64.75801588 |
| 55 | C | 53.55285000 | 62.54721900 | 57.44450800 | 117 | H | 49.91124793 | 66.29636269 | 55.80811116 |
| 56 | H | 53.74241100 | 63.62461000 | 57.31582700 | 118 | H | 47.36409532 | 64.53231865 | 61.35064760 |
| 57 | H | 54.23227500 | 62.22578000 | 58.25300600 | 119 | H | 50.89047844 | 68.92391811 | 63.86778295 |
| 58 | C | 52.07229300 | 62.33961300 | 57.84770600 | 120 | H | 49.01684082 | 65.01306415 | 66.32949761 |
| 59 | H | 51.46759900 | 62.28531000 | 56.92735800 | 121 | H | 53.86117789 | 62.07741695 | 56.53394776 |
| 60 | C | 51.82328800 | 61.05578600 | 58.64866700 | 122 | H | 58.42441341 | 66.56870343 | 54.79706339 |
| 61 | H | 52.40821600 | 61.06000100 | 59.58646800 | 123 | H | 56.29234631 | 68.26938489 | 63.78772263 |
| 62 | H | 52.09717800 | 60.14356300 | 58.10106500 |  |  |  |  |  |

| **IN2** | | | | | | | | | |
| --- | --- | --- | --- | --- | --- | --- | --- | --- | --- |
| 1 | C | 50.85015300 | 66.93150700 | 55.87366200 | 63 | H | 52.77181700 | 61.46121600 | 59.71083600 |
| 2 | H | 51.25718300 | 66.74860200 | 56.88700500 | 64 | C | 52.11030900 | 63.98880900 | 58.74478000 |
| 3 | H | 50.67956900 | 68.01639200 | 55.78941400 | 65 | H | 52.64543200 | 64.08525200 | 59.70366100 |
| 4 | C | 51.88245300 | 66.46401800 | 54.82406500 | 66 | H | 52.29117700 | 64.92043000 | 58.18920600 |
| 5 | H | 51.40931300 | 66.46161600 | 53.82868800 | 67 | H | 51.03028300 | 63.93489500 | 58.95847100 |
| 6 | C | 53.04958800 | 67.44808200 | 54.76871000 | 68 | C | 58.85192600 | 66.30194800 | 55.80964100 |
| 7 | H | 52.72992700 | 68.43705200 | 54.40901200 | 69 | H | 59.67151700 | 66.93422700 | 56.19810300 |
| 8 | H | 53.51823900 | 67.59059000 | 55.75823700 | 70 | H | 59.30375100 | 65.31756700 | 55.62365300 |
| 9 | H | 53.84565600 | 67.10094100 | 54.09328100 | 71 | C | 57.78245100 | 66.19254700 | 56.86484800 |
| 10 | C | 52.38797900 | 65.03741100 | 55.06736400 | 72 | C | 56.77618800 | 67.16181300 | 57.01235800 |
| 11 | H | 52.99030600 | 64.99185200 | 55.98876600 | 73 | H | 56.72418500 | 68.01805900 | 56.33430000 |
| 12 | H | 51.57608400 | 64.30219400 | 55.15819900 | 74 | C | 55.81534500 | 67.05516200 | 58.01702900 |
| 13 | H | 53.04556000 | 64.70748000 | 54.24711400 | 75 | H | 55.04865900 | 67.82311800 | 58.11298800 |
| 14 | C | 48.12599000 | 65.89962300 | 61.54507900 | 76 | C | 55.82994100 | 65.96596100 | 58.89077100 |
| 15 | H | 47.76873600 | 66.31060400 | 62.50367900 | 77 | H | 55.05398900 | 65.87600600 | 59.65091700 |
| 16 | H | 47.73787200 | 66.51641000 | 60.72651000 | 78 | C | 56.83051100 | 65.00121700 | 58.76779300 |
| 17 | C | 49.61717100 | 65.96353600 | 61.58246700 | 79 | H | 56.85548800 | 64.13949400 | 59.43980600 |
| 18 | O | 50.22734000 | 65.34788500 | 62.62517000 | 80 | C | 57.79623600 | 65.11797200 | 57.76743000 |
| 19 | O | 50.29755500 | 66.53339500 | 60.76630500 | 81 | H | 58.57064400 | 64.35342400 | 57.68103200 |
| 20 | C | 48.97284500 | 64.37744800 | 65.77440200 | 82 | C | 56.66751200 | 68.60138700 | 62.88209100 |
| 21 | H | 48.43361900 | 64.51941700 | 64.81274500 | 83 | H | 57.43680000 | 69.05399200 | 62.24044800 |
| 22 | C | 50.21723000 | 63.51566300 | 65.50549600 | 84 | C | 56.22638800 | 67.30231600 | 62.20762000 |
| 23 | H | 50.95702300 | 63.99849300 | 64.84793800 | 85 | H | 57.07373200 | 66.65049800 | 61.94142800 |
| 24 | H | 50.71757100 | 63.23817100 | 66.44699300 | 86 | H | 55.53970000 | 66.73604300 | 62.85082400 |
| 25 | H | 49.92480200 | 62.56307200 | 65.03275800 | 87 | H | 55.70584600 | 67.50712400 | 61.26371400 |
| 26 | C | 48.04260400 | 63.64339600 | 66.73007300 | 88 | C | 55.51691100 | 69.62550900 | 63.01570700 |
| 27 | H | 47.60368800 | 62.76621200 | 66.23552900 | 89 | H | 55.87517100 | 70.51086200 | 63.56481800 |
| 28 | H | 48.60045600 | 63.24099600 | 67.58655800 | 90 | H | 54.71142700 | 69.19870100 | 63.63514300 |
| 29 | H | 47.23483100 | 64.27359100 | 67.11379900 | 91 | C | 54.92942100 | 70.11033600 | 61.68953500 |
| 30 | C | 51.79728400 | 68.28355400 | 63.94950300 | 92 | H | 54.19487500 | 70.91444200 | 61.85470900 |
| 31 | H | 52.20830900 | 68.17645500 | 62.93313700 | 93 | H | 55.71702100 | 70.51136900 | 61.03273600 |
| 32 | H | 50.95215100 | 67.58009800 | 64.00393100 | 94 | H | 54.41337800 | 69.31012700 | 61.13929000 |
| 33 | C | 52.86723800 | 67.87318200 | 64.92984500 | 95 | C | 57.70573100 | 62.75223500 | 63.99117000 |
| 34 | N | 53.64166500 | 66.74735100 | 64.69629200 | 96 | H | 57.64880200 | 61.84633500 | 63.36945100 |
| 35 | C | 54.53385200 | 66.68476100 | 65.66697900 | 97 | H | 58.71764200 | 63.14926800 | 63.83714800 |
| 36 | H | 55.31779600 | 65.93458300 | 65.75586300 | 98 | C | 56.64275800 | 63.75500400 | 63.51657800 |
| 37 | N | 54.35490000 | 67.68778800 | 66.55444000 | 99 | H | 57.07438700 | 64.35022100 | 62.69850100 |
| 38 | C | 53.30237700 | 68.45645000 | 66.10369600 | 100 | H | 56.38570600 | 64.48628800 | 64.29878200 |
| 39 | H | 52.97497700 | 69.33614100 | 66.65328800 | 101 | C | 55.36290400 | 63.10016900 | 62.98083000 |
| 40 | C | 49.35594900 | 72.85071200 | 58.26254200 | 102 | H | 54.70008600 | 62.74730100 | 63.79141900 |
| 41 | H | 49.12322100 | 73.11804200 | 57.21596200 | 103 | H | 55.66642200 | 62.19778400 | 62.43624900 |
| 42 | H | 48.39842700 | 72.57879200 | 58.72891400 | 104 | C | 54.59366900 | 63.94510300 | 61.97017700 |
| 43 | C | 50.30084100 | 71.67686500 | 58.30210300 | 105 | H | 54.01854000 | 63.30212900 | 61.28917800 |
| 44 | C | 51.65146800 | 71.79060100 | 57.92873500 | 106 | H | 55.29551800 | 64.51859300 | 61.35136900 |
| 45 | H | 52.04864400 | 72.74243600 | 57.56343800 | 107 | N | 53.61091400 | 64.92613400 | 62.52899100 |
| 46 | C | 52.51383700 | 70.69671300 | 58.00004400 | 108 | H | 53.87766600 | 65.49647100 | 63.38093900 |
| 47 | H | 53.56310000 | 70.78821600 | 57.71015000 | 109 | H | 53.37957900 | 65.66625100 | 61.80351800 |
| 48 | C | 52.02729300 | 69.46310000 | 58.43168600 | 110 | H | 52.75376900 | 64.43019400 | 62.79149400 |
| 49 | O | 52.90792500 | 68.38876100 | 58.52470600 | 111 | O | 52.93229800 | 66.93509900 | 60.89072900 |
| 50 | H | 52.63649200 | 67.69679300 | 57.90190800 | 112 | H | 51.96374300 | 66.82514000 | 60.78669900 |
| 51 | C | 50.68883800 | 69.31616000 | 58.79753700 | 113 | H | 53.16558900 | 67.55849100 | 60.17168200 |
| 52 | H | 50.31958000 | 68.35036500 | 59.14644000 | 114 | H | 54.90829600 | 67.82606800 | 67.40767500 |
| 53 | C | 49.84434200 | 70.42457400 | 58.73306400 | 115 | H | 49.58596000 | 64.96421000 | 63.24673100 |
| 54 | H | 48.79860300 | 70.32318600 | 59.02738400 | 116 | H | 49.71632796 | 73.74045813 | 58.73517170 |
| 55 | C | 54.08878400 | 62.87066000 | 57.59639000 | 117 | H | 51.41811336 | 69.27963480 | 64.04412156 |
| 56 | H | 54.34473600 | 63.93362000 | 57.46971700 | 118 | H | 57.11738889 | 68.44128448 | 63.83962845 |
| 57 | H | 54.72529700 | 62.50857200 | 58.42193100 | 119 | H | 57.64911187 | 62.44465643 | 65.01444401 |
| 58 | C | 52.58720200 | 62.75641200 | 57.95998300 | 120 | H | 49.25397152 | 65.35901908 | 66.09438027 |
| 59 | H | 52.00409100 | 62.73903100 | 57.02328100 | 121 | H | 49.89522484 | 66.45014719 | 55.83753808 |
| 60 | C | 52.22847700 | 61.49412900 | 58.74890300 | 122 | H | 47.74709390 | 64.90197147 | 61.46743131 |
| 61 | H | 51.15165600 | 61.46547100 | 58.97669100 | 123 | H | 54.39213044 | 62.38171165 | 56.69427557 |
| 62 | H | 52.46760500 | 60.56416900 | 58.21228600 | 124 | H | 58.55908470 | 66.71427358 | 54.86670326 |

| **RC** | | | | | | | | | |
| --- | --- | --- | --- | --- | --- | --- | --- | --- | --- |
| 1 | C | 50.99262900 | 67.09882500 | 56.02388400 | 63 | H | 52.90595500 | 61.46417400 | 59.54815200 |
| 2 | H | 51.40895500 | 66.88332000 | 57.01719800 | 64 | C | 52.20459100 | 63.96414900 | 58.61298500 |
| 3 | H | 50.82828600 | 68.18013500 | 55.99003900 | 65 | H | 52.75225500 | 64.04823300 | 59.56032300 |
| 4 | C | 52.03015400 | 66.68965500 | 54.95193400 | 66 | H | 52.36009600 | 64.90020800 | 58.07111400 |
| 5 | H | 51.69565600 | 67.06818000 | 53.98147100 | 67 | H | 51.13486500 | 63.88518300 | 58.84137100 |
| 6 | C | 53.38092200 | 67.34365800 | 55.25253800 | 68 | C | 58.94802100 | 66.35153000 | 55.75371300 |
| 7 | H | 53.29747900 | 68.42826300 | 55.36169000 | 69 | H | 59.75336500 | 66.99119700 | 56.14155400 |
| 8 | H | 53.81818900 | 66.95531600 | 56.17750400 | 70 | H | 59.41854300 | 65.39204200 | 55.52652400 |
| 9 | H | 54.09886600 | 67.15141500 | 54.44788700 | 71 | C | 57.91339100 | 66.16759200 | 56.83472600 |
| 10 | C | 52.20128300 | 65.16925500 | 54.80193900 | 72 | C | 56.87624600 | 67.08431400 | 57.05411600 |
| 11 | H | 52.55807800 | 64.72401600 | 55.73622600 | 73 | H | 56.75925600 | 67.94624500 | 56.40264700 |
| 12 | H | 51.26788200 | 64.67495300 | 54.52403200 | 74 | C | 55.97322900 | 66.91198600 | 58.10185300 |
| 13 | H | 52.94640100 | 64.93977400 | 54.03287000 | 75 | H | 55.18673700 | 67.64337400 | 58.24820100 |
| 14 | C | 48.20729400 | 65.99535400 | 61.44594900 | 76 | C | 56.07910100 | 65.80084800 | 58.93953100 |
| 15 | H | 47.93132400 | 66.41936700 | 62.41742600 | 77 | H | 55.35742800 | 65.65472900 | 59.73363800 |
| 16 | H | 47.72234000 | 66.60936900 | 60.68525200 | 78 | C | 57.10365900 | 64.88081300 | 58.73247200 |
| 17 | C | 49.69019100 | 66.17422700 | 61.37290800 | 79 | H | 57.18845400 | 64.00615600 | 59.37099200 |
| 18 | O | 50.49091200 | 65.08838300 | 61.43104600 | 80 | C | 58.01438100 | 65.06891700 | 57.69627100 |
| 19 | O | 50.20506100 | 67.28044200 | 61.36491300 | 81 | H | 58.81723500 | 64.35304900 | 57.55452200 |
| 20 | C | 48.86161100 | 64.54442100 | 65.74086000 | 82 | C | 56.47506600 | 68.68244600 | 62.81005100 |
| 21 | H | 48.09807500 | 64.72319000 | 64.97342400 | 83 | H | 57.25230900 | 69.09015100 | 62.15747100 |
| 22 | C | 50.04767700 | 63.87317000 | 65.02756300 | 84 | C | 55.95946400 | 67.39630300 | 62.15978300 |
| 23 | H | 50.46534300 | 64.48879200 | 64.22109400 | 85 | H | 56.76252300 | 66.69273700 | 61.91661100 |
| 24 | H | 50.84983300 | 63.62095600 | 65.73338000 | 86 | H | 55.22914400 | 66.89459600 | 62.80368900 |
| 25 | H | 49.73045200 | 62.92339500 | 64.58354900 | 87 | H | 55.46148700 | 67.61664800 | 61.21533000 |
| 26 | C | 48.28021000 | 63.59425600 | 66.78298000 | 88 | C | 55.35915800 | 69.74714500 | 62.93921700 |
| 27 | H | 47.84761200 | 62.71812400 | 66.29267500 | 89 | H | 55.73645000 | 70.61635100 | 63.48871800 |
| 28 | H | 49.05309100 | 63.20869100 | 67.45504900 | 90 | H | 54.54062500 | 69.34246500 | 63.54680100 |
| 29 | H | 47.50139100 | 64.06060900 | 67.38181500 | 91 | C | 54.80406000 | 70.25034900 | 61.60292900 |
| 30 | C | 51.65234200 | 68.55060000 | 63.98053200 | 92 | H | 54.06843900 | 71.04542600 | 61.76215600 |
| 31 | H | 52.15055300 | 68.53312100 | 63.00950700 | 93 | H | 55.60720000 | 70.66547400 | 60.98760600 |
| 32 | H | 50.83084800 | 67.83071200 | 63.93115900 | 94 | H | 54.31788300 | 69.46822800 | 61.01426300 |
| 33 | C | 52.65931900 | 68.12709200 | 65.00791900 | 95 | C | 57.63886500 | 62.79153400 | 63.75360900 |
| 34 | N | 53.34978500 | 66.93781300 | 64.83739200 | 96 | H | 57.61448700 | 61.86726700 | 63.17009900 |
| 35 | C | 54.25216100 | 66.88279500 | 65.80229700 | 97 | H | 58.64395600 | 63.19262300 | 63.62584600 |
| 36 | H | 54.97853300 | 66.09687400 | 65.93165000 | 98 | C | 56.57792100 | 63.75578500 | 63.20897200 |
| 37 | N | 54.15098900 | 67.94578000 | 66.62372900 | 99 | H | 56.98618000 | 64.33528400 | 62.37645600 |
| 38 | C | 53.14460000 | 68.75289500 | 66.13245700 | 100 | H | 56.31188700 | 64.50764700 | 63.96412000 |
| 39 | H | 52.88377900 | 69.67908700 | 66.62085500 | 101 | C | 55.30073700 | 63.05977700 | 62.71527700 |
| 40 | C | 49.35034400 | 72.89904400 | 58.28884300 | 102 | H | 54.99141600 | 62.26940600 | 63.41223200 |
| 41 | H | 49.08229900 | 73.17505900 | 57.26025300 | 103 | H | 55.47890600 | 62.56601900 | 61.75811200 |
| 42 | H | 48.42443200 | 72.58710700 | 58.77874400 | 104 | C | 54.14316400 | 64.03131900 | 62.53374500 |
| 43 | C | 50.34705200 | 71.76666000 | 58.29428400 | 105 | H | 53.31385900 | 63.59291300 | 61.97892000 |
| 44 | C | 51.65863600 | 71.92697700 | 57.81824200 | 106 | H | 54.44113100 | 64.95306900 | 62.03852400 |
| 45 | H | 51.97070100 | 72.87145400 | 57.37760600 | 107 | N | 53.59655300 | 64.40274300 | 63.89609400 |
| 46 | C | 52.58832300 | 70.89748300 | 57.89507400 | 108 | H | 53.32526900 | 65.43613700 | 64.05715800 |
| 47 | H | 53.60370500 | 71.03235300 | 57.53626200 | 109 | H | 52.83367100 | 63.75848700 | 64.14572900 |
| 48 | C | 52.21773700 | 69.66489800 | 58.44060600 | 110 | H | 54.29848000 | 64.19481100 | 64.62412800 |
| 49 | O | 53.17333000 | 68.69110100 | 58.50713300 | 111 | O | 52.75693800 | 66.86322900 | 61.54479100 |
| 50 | H | 52.83155400 | 67.89296000 | 58.94046700 | 112 | H | 51.80256500 | 67.15270900 | 61.41163400 |
| 51 | C | 50.90883000 | 69.46895000 | 58.88465300 | 113 | H | 54.71186600 | 68.08397100 | 67.46532200 |
| 52 | H | 50.60694300 | 68.50911800 | 59.29071600 | 114 | O | 53.20846100 | 66.57629400 | 60.32671800 |
| 53 | C | 49.99614600 | 70.52003100 | 58.81404600 | 115 | H | 49.97189000 | 64.27577900 | 61.33507500 |
| 54 | H | 48.98092700 | 70.37130500 | 59.16602700 | 116 | H | 49.69205170 | 73.79094795 | 58.77117400 |
| 55 | C | 54.18705800 | 62.90475500 | 57.43803500 | 117 | H | 49.14706377 | 65.50906583 | 66.10538308 |
| 56 | H | 54.40994500 | 63.96809700 | 57.30470100 | 118 | H | 56.92738320 | 68.50782875 | 63.76389479 |
| 57 | H | 54.83192800 | 62.56490800 | 58.25753600 | 119 | H | 47.84365154 | 64.98968743 | 61.40990874 |
| 58 | C | 52.69036900 | 62.75066900 | 57.80625500 | 120 | H | 50.03954521 | 66.61514201 | 55.97307012 |
| 59 | H | 52.10518400 | 62.73122400 | 56.87838300 | 121 | H | 51.24362255 | 69.53349000 | 64.08904775 |
| 60 | C | 52.36422300 | 61.47588100 | 58.59223000 | 122 | H | 57.53083983 | 62.52487891 | 64.78420379 |
| 61 | H | 51.29404400 | 61.42129300 | 58.81379000 | 123 | H | 54.49402670 | 62.41677384 | 56.53662244 |
| 62 | H | 52.62631000 | 60.55883400 | 58.05996600 | 124 | H | 58.62951340 | 66.78285789 | 54.82774909 |

| **TS1** | | | | | | | | | |
| --- | --- | --- | --- | --- | --- | --- | --- | --- | --- |
| 1 | C | 51.05218500 | 66.82371300 | 55.67015600 | 63 | H | 52.78788000 | 61.46989200 | 59.46674800 |
| 2 | H | 51.52435300 | 66.69448800 | 56.64842500 | 64 | C | 52.10929200 | 63.95658700 | 58.45868600 |
| 3 | H | 50.86821500 | 67.89927300 | 55.57244100 | 65 | H | 52.61867300 | 64.05811100 | 59.42685300 |
| 4 | C | 52.02331700 | 66.35497400 | 54.56978200 | 66 | H | 52.26459900 | 64.89017100 | 57.91633100 |
| 5 | H | 51.52541800 | 66.37346300 | 53.59323100 | 67 | H | 51.03462000 | 63.86971100 | 58.66652600 |
| 6 | C | 53.20293700 | 67.32292900 | 54.51266600 | 68 | C | 58.80525400 | 66.38651400 | 55.73370700 |
| 7 | H | 52.86334900 | 68.34788200 | 54.33176900 | 69 | H | 59.58638600 | 67.04649800 | 56.13509400 |
| 8 | H | 53.75933400 | 67.32588500 | 55.45749200 | 70 | H | 59.28988700 | 65.42072200 | 55.57152600 |
| 9 | H | 53.91102900 | 67.07231900 | 53.72069300 | 71 | C | 57.70716200 | 66.25011000 | 56.75963500 |
| 10 | C | 52.51921200 | 64.91673100 | 54.77684200 | 72 | C | 56.64581900 | 67.16117300 | 56.84673500 |
| 11 | H | 53.12424400 | 64.86009500 | 55.68803600 | 73 | H | 56.56231400 | 67.98964800 | 56.14873700 |
| 12 | H | 51.70138400 | 64.19765700 | 54.87329800 | 74 | C | 55.65128300 | 67.01875800 | 57.81027800 |
| 13 | H | 53.15905700 | 64.59032500 | 53.95155900 | 75 | H | 54.84048800 | 67.73559400 | 57.82540200 |
| 14 | C | 48.39932800 | 65.88168200 | 61.44267100 | 76 | C | 55.70450700 | 65.96520200 | 58.72175000 |
| 15 | H | 48.11795900 | 66.31177700 | 62.41071600 | 77 | H | 54.90598900 | 65.84023200 | 59.44738200 |
| 16 | H | 47.98616300 | 66.54337300 | 60.67926900 | 78 | C | 56.76253400 | 65.05863200 | 58.66191600 |
| 17 | C | 49.89480400 | 66.02291100 | 61.40772800 | 79 | H | 56.80744000 | 64.22071100 | 59.35324600 |
| 18 | O | 50.72510300 | 64.93920700 | 61.48639700 | 80 | C | 57.74757000 | 65.19996100 | 57.68580700 |
| 19 | O | 50.43091200 | 67.10863900 | 61.40299200 | 81 | H | 58.55027500 | 64.47273400 | 57.62789600 |
| 20 | C | 48.87713500 | 64.47589100 | 65.77521300 | 82 | C | 56.43276300 | 68.73403000 | 62.80365600 |
| 21 | H | 48.15134500 | 64.66160100 | 64.97334400 | 83 | H | 57.18625100 | 69.17692200 | 62.14719000 |
| 22 | C | 50.06764300 | 63.75377500 | 65.12867200 | 84 | C | 55.89556500 | 67.49385800 | 62.08231100 |
| 23 | H | 50.61136600 | 64.38373200 | 64.41175100 | 85 | H | 56.68702700 | 66.78676200 | 61.81083600 |
| 24 | H | 50.77391200 | 63.38827300 | 65.88034400 | 86 | H | 55.14780500 | 66.97209900 | 62.68852700 |
| 25 | H | 49.71994500 | 62.86995200 | 64.58369700 | 87 | H | 55.42295200 | 67.78605400 | 61.14026100 |
| 26 | C | 48.22385000 | 63.56037600 | 66.80439400 | 88 | C | 55.33015600 | 69.79565500 | 63.04228200 |
| 27 | H | 47.79423100 | 62.68665100 | 66.30798400 | 89 | H | 55.74012100 | 70.62661400 | 63.62505300 |
| 28 | H | 48.95673600 | 63.16809000 | 67.51419100 | 90 | H | 54.53692600 | 69.36243500 | 63.66242600 |
| 29 | H | 47.43295800 | 64.05926200 | 67.35685100 | 91 | C | 54.71628500 | 70.37930900 | 61.77020100 |
| 30 | C | 51.67960100 | 68.41183800 | 63.97680500 | 92 | H | 54.02117300 | 71.19100600 | 62.00971400 |
| 31 | H | 52.08098000 | 68.34439600 | 62.96149600 | 93 | H | 55.48879700 | 70.78809200 | 61.11170400 |
| 32 | H | 50.83029200 | 67.72263200 | 64.00471700 | 94 | H | 54.16807800 | 69.64035200 | 61.18396600 |
| 33 | C | 52.74248900 | 67.96127900 | 64.93371900 | 95 | C | 57.41966100 | 62.81205800 | 63.82162800 |
| 34 | N | 53.48364000 | 66.82074600 | 64.65864800 | 96 | H | 57.34183600 | 61.89280500 | 63.23425300 |
| 35 | C | 54.37128300 | 66.69214400 | 65.62866100 | 97 | H | 58.41320900 | 63.20839700 | 63.61241500 |
| 36 | H | 55.10354300 | 65.90266500 | 65.69864000 | 98 | C | 56.32311400 | 63.79990900 | 63.36335500 |
| 37 | N | 54.22168800 | 67.67455100 | 66.54257800 | 99 | H | 56.75963900 | 64.53952400 | 62.68481100 |
| 38 | C | 53.19684700 | 68.49235800 | 66.11599600 | 100 | H | 55.92019400 | 64.37364000 | 64.20716500 |
| 39 | H | 52.89976100 | 69.35970200 | 66.68495300 | 101 | C | 55.16659000 | 63.11435900 | 62.63021800 |
| 40 | C | 49.30289900 | 72.98978400 | 58.25490200 | 102 | H | 54.66601100 | 62.38409400 | 63.27938400 |
| 41 | H | 49.08929000 | 73.29942800 | 57.22499700 | 103 | H | 55.56582900 | 62.52598100 | 61.80163400 |
| 42 | H | 48.37314500 | 72.59485400 | 58.67131200 | 104 | C | 54.13152400 | 64.04515200 | 62.00733600 |
| 43 | C | 50.39444600 | 71.94692000 | 58.28465900 | 105 | H | 53.52012300 | 63.49269900 | 61.29193300 |
| 44 | C | 51.59984000 | 72.13210900 | 57.55143800 | 106 | H | 54.60059200 | 64.87708300 | 61.47912400 |
| 45 | H | 51.69176100 | 72.98073600 | 56.88093400 | 107 | N | 53.16693700 | 64.64314900 | 63.00537300 |
| 46 | C | 52.69043600 | 71.28954700 | 57.74358400 | 108 | H | 53.42867100 | 65.55689800 | 63.51381000 |
| 47 | H | 53.62598900 | 71.44192000 | 57.22145900 | 109 | H | 52.26804400 | 64.81359900 | 62.52636600 |
| 48 | C | 52.59576700 | 70.22370100 | 58.62715100 | 110 | H | 52.98935100 | 63.96275200 | 63.75593100 |
| 49 | O | 53.64946100 | 69.39472500 | 58.81272500 | 111 | O | 51.90241300 | 67.37635900 | 59.00949500 |
| 50 | H | 53.26119300 | 68.51876500 | 59.03755400 | 112 | H | 51.55001200 | 67.13806300 | 59.88789300 |
| 51 | C | 51.37286800 | 69.94886800 | 59.32974900 | 113 | H | 54.77701700 | 67.79107900 | 67.39080800 |
| 52 | H | 51.45281700 | 69.45398900 | 60.29437000 | 114 | O | 50.91491300 | 68.30661500 | 58.54554900 |
| 53 | C | 50.30192500 | 70.87444300 | 59.14566700 | 115 | H | 50.30764100 | 64.04193100 | 61.44389700 |
| 54 | H | 49.39389900 | 70.74735300 | 59.71996300 | 116 | H | 49.55800252 | 73.87076877 | 58.80598096 |
| 55 | C | 54.14215300 | 62.86858100 | 57.39710200 | 117 | H | 56.91365712 | 68.52958386 | 63.73738113 |
| 56 | H | 54.38291900 | 63.92851600 | 57.27991600 | 118 | H | 49.16463307 | 65.44110654 | 66.13660451 |
| 57 | H | 54.74625400 | 62.51632900 | 58.24259400 | 119 | H | 57.40507343 | 62.53065600 | 64.85385865 |
| 58 | C | 52.62969800 | 62.72913500 | 57.70110100 | 120 | H | 47.95414385 | 64.91142469 | 61.36978873 |
| 59 | H | 52.08454700 | 62.69097600 | 56.74993300 | 121 | H | 58.52471690 | 66.78048138 | 54.77924975 |
| 60 | C | 52.27554400 | 61.46760200 | 58.49401800 | 122 | H | 54.48077246 | 62.37991023 | 56.50747413 |
| 61 | H | 51.20046500 | 61.43565600 | 58.69143300 | 123 | H | 51.30709915 | 69.40701208 | 64.10238732 |
| 62 | H | 52.54513300 | 60.54065900 | 57.98546000 | 124 | H | 50.09949079 | 66.33747318 | 55.69921773 |

| **IM1** | | | | | | | | | |
| --- | --- | --- | --- | --- | --- | --- | --- | --- | --- |
| 1 | C | 50.96706500 | 67.03265800 | 55.87619100 | 63 | H | 52.70004200 | 61.38353100 | 59.46262800 |
| 2 | H | 51.38709800 | 66.89110000 | 56.87940500 | 64 | C | 52.16981200 | 63.94324100 | 58.63590600 |
| 3 | H | 50.77946000 | 68.10636800 | 55.77107600 | 65 | H | 52.71491700 | 63.96373500 | 59.58970800 |
| 4 | C | 51.99694300 | 66.56922600 | 54.82150800 | 66 | H | 52.33303200 | 64.90449700 | 58.14511700 |
| 5 | H | 51.55728700 | 66.64980400 | 53.82107600 | 67 | H | 51.09871800 | 63.87206500 | 58.86914800 |
| 6 | C | 53.22012200 | 67.48576100 | 54.86210300 | 68 | C | 58.86241800 | 66.37074000 | 55.75396800 |
| 7 | H | 52.95888800 | 68.51200700 | 54.59437200 | 69 | H | 59.66087000 | 67.01228600 | 56.15179000 |
| 8 | H | 53.67223600 | 67.50333200 | 55.86012100 | 70 | H | 59.33072200 | 65.40200200 | 55.56316200 |
| 9 | H | 53.99457100 | 67.15668900 | 54.16230500 | 71 | C | 57.78799600 | 66.22565400 | 56.80338700 |
| 10 | C | 52.43326800 | 65.10736500 | 54.99027600 | 72 | C | 56.75738900 | 67.16286400 | 56.95960500 |
| 11 | H | 52.97405800 | 64.97909900 | 55.93345200 | 73 | H | 56.68008300 | 68.01353000 | 56.28788900 |
| 12 | H | 51.59550400 | 64.40555700 | 54.98262400 | 74 | C | 55.80214900 | 67.02410500 | 57.96426500 |
| 13 | H | 53.12099100 | 64.81158200 | 54.19091500 | 75 | H | 55.01779400 | 67.76480800 | 58.06050400 |
| 14 | C | 48.63239700 | 65.58986400 | 61.49419300 | 76 | C | 55.85521700 | 65.93188700 | 58.83038700 |
| 15 | H | 48.19418900 | 66.27315900 | 62.22734400 | 77 | H | 55.09394500 | 65.80566400 | 59.59496100 |
| 16 | H | 48.68355200 | 66.14598500 | 60.55381600 | 78 | C | 56.87693300 | 64.99408400 | 58.69492400 |
| 17 | C | 50.03864800 | 65.35983200 | 61.99383700 | 79 | H | 56.92080200 | 64.13280500 | 59.35602200 |
| 18 | O | 50.52528900 | 64.11635700 | 62.26838800 | 80 | C | 57.83248500 | 65.14428600 | 57.69218000 |
| 19 | O | 50.77788500 | 66.28746500 | 62.26806300 | 81 | H | 58.62082900 | 64.40569000 | 57.59297000 |
| 20 | C | 49.07123400 | 64.45670000 | 65.87337200 | 82 | C | 56.46737700 | 68.69092600 | 62.77600000 |
| 21 | H | 48.62427700 | 64.56634800 | 64.87560900 | 83 | H | 57.23790100 | 69.10913300 | 62.12275700 |
| 22 | C | 50.35537700 | 63.62711000 | 65.71577800 | 84 | C | 55.94070100 | 67.42451500 | 62.09210700 |
| 23 | H | 51.11797300 | 64.12854100 | 65.11195300 | 85 | H | 56.73704200 | 66.71606300 | 61.84065500 |
| 24 | H | 50.79300100 | 63.37953700 | 66.68889200 | 86 | H | 55.19807200 | 66.91310300 | 62.71388200 |
| 25 | H | 50.12407300 | 62.67201400 | 65.23039200 | 87 | H | 55.46372500 | 67.68236900 | 61.14353200 |
| 26 | C | 48.10027700 | 63.69490100 | 66.76830000 | 88 | C | 55.35698700 | 69.75816900 | 62.93997800 |
| 27 | H | 47.69945200 | 62.83150600 | 66.23331100 | 89 | H | 55.74363500 | 70.61220200 | 63.50568600 |
| 28 | H | 48.60801800 | 63.28470200 | 67.64294900 | 90 | H | 54.54349200 | 69.34460700 | 63.54691100 |
| 29 | H | 47.27316500 | 64.30950200 | 67.11186000 | 91 | C | 54.78916700 | 70.29502800 | 61.62296800 |
| 30 | C | 51.71771400 | 68.49427700 | 64.04916400 | 92 | H | 54.06739300 | 71.09687600 | 61.81102700 |
| 31 | H | 52.12489800 | 68.42899300 | 63.03552200 | 93 | H | 55.58681900 | 70.71133700 | 61.00095500 |
| 32 | H | 50.88514900 | 67.78953100 | 64.07464200 | 94 | H | 54.28397700 | 69.53075300 | 61.02634600 |
| 33 | C | 52.78706300 | 68.05520200 | 65.00117700 | 95 | C | 57.48520800 | 62.82899500 | 63.81955900 |
| 34 | N | 53.52527000 | 66.91709700 | 64.71611000 | 96 | H | 57.45112600 | 61.91529800 | 63.21939400 |
| 35 | C | 54.42620200 | 66.78994300 | 65.67319000 | 97 | H | 58.47680500 | 63.24911400 | 63.65562100 |
| 36 | H | 55.16274200 | 66.00220000 | 65.72703200 | 98 | C | 56.38498900 | 63.78891400 | 63.33088400 |
| 37 | N | 54.28922900 | 67.77199100 | 66.59015700 | 99 | H | 56.78392000 | 64.42657900 | 62.53594500 |
| 38 | C | 53.25335600 | 68.58610700 | 66.17922700 | 100 | H | 56.07067500 | 64.47720000 | 64.12528300 |
| 39 | H | 52.96192000 | 69.45202500 | 66.75385900 | 101 | C | 55.15991900 | 63.05338000 | 62.77798200 |
| 40 | C | 49.36285200 | 72.93370100 | 58.25767300 | 102 | H | 54.76451100 | 62.34364700 | 63.51812300 |
| 41 | H | 49.16738600 | 73.20257700 | 57.21255600 | 103 | H | 55.46457200 | 62.44478800 | 61.92352100 |
| 42 | H | 48.40797000 | 72.64399000 | 58.70187300 | 104 | C | 54.02371900 | 63.94685800 | 62.29249100 |
| 43 | C | 50.34301100 | 71.79341900 | 58.34576100 | 105 | H | 53.32397300 | 63.37639900 | 61.67998400 |
| 44 | C | 51.62445000 | 71.88047000 | 57.72135200 | 106 | H | 54.38809700 | 64.78719800 | 61.69837300 |
| 45 | H | 51.88072500 | 72.74901900 | 57.12249000 | 107 | N | 53.21787700 | 64.51863600 | 63.43636700 |
| 46 | C | 52.55902300 | 70.83972800 | 57.85724200 | 108 | H | 53.41712600 | 65.53350000 | 63.73812500 |
| 47 | H | 53.52323000 | 70.88912800 | 57.36089000 | 109 | H | 52.22089800 | 64.46939600 | 63.20308900 |
| 48 | C | 52.26821600 | 69.73561900 | 58.61807600 | 110 | H | 53.39086200 | 63.95100600 | 64.28168600 |
| 49 | O | 53.17861500 | 68.74265400 | 58.78632400 | 111 | O | 51.11060800 | 67.24619300 | 59.63201500 |
| 50 | H | 52.69895400 | 67.96365400 | 59.13281300 | 112 | H | 50.91178100 | 67.23267800 | 60.58896100 |
| 51 | C | 50.99398700 | 69.61554200 | 59.39614200 | 113 | H | 54.84591500 | 67.87344800 | 67.43991300 |
| 52 | H | 51.23939600 | 69.58016500 | 60.47680700 | 114 | O | 50.28441500 | 68.35292600 | 59.13852200 |
| 53 | C | 50.02719400 | 70.70253700 | 59.10133100 | 115 | H | 50.10428700 | 63.37814600 | 61.74219500 |
| 54 | H | 49.05005000 | 70.64064600 | 59.55965600 | 116 | H | 49.69819021 | 73.82003195 | 58.75452884 |
| 55 | C | 54.14461700 | 62.86875700 | 57.45793900 | 117 | H | 50.02188698 | 66.53180135 | 55.85009255 |
| 56 | H | 54.40524500 | 63.92635100 | 57.35809400 | 118 | H | 48.00017679 | 64.73184687 | 61.39930246 |
| 57 | H | 54.74931300 | 62.48907700 | 58.29108000 | 119 | H | 56.92984231 | 68.50884623 | 63.72356150 |
| 58 | C | 52.63222200 | 62.75720600 | 57.77567700 | 120 | H | 49.29654678 | 65.45025094 | 66.20046629 |
| 59 | H | 52.07609600 | 62.81641100 | 56.83198100 | 121 | H | 51.32900315 | 69.48366937 | 64.17125662 |
| 60 | C | 52.22935000 | 61.45243000 | 58.47206100 | 122 | H | 58.57264653 | 66.78443411 | 54.81068136 |
| 61 | H | 51.14558100 | 61.41893900 | 58.61210800 | 123 | H | 54.46540833 | 62.39091590 | 56.55590751 |
| 62 | H | 52.50825800 | 60.55592400 | 57.91643100 | 124 | H | 57.43495225 | 62.53871871 | 64.84820570 |

| **TS2** | | | | | | | | | |
| --- | --- | --- | --- | --- | --- | --- | --- | --- | --- |
| 1 | C | 51.00879900 | 66.83238800 | 55.55070200 | 63 | H | 52.54442900 | 61.32898200 | 59.28464800 |
| 2 | H | 51.47711100 | 66.76544400 | 56.53880200 | 64 | C | 52.15438700 | 63.90616800 | 58.50660100 |
| 3 | H | 50.82325900 | 67.89590000 | 55.37279000 | 65 | H | 52.60477900 | 63.84511400 | 59.50778200 |
| 4 | C | 51.99108800 | 66.31487800 | 54.48453100 | 66 | H | 52.42752200 | 64.87879800 | 58.08902700 |
| 5 | H | 51.49817200 | 66.27992300 | 53.50534800 | 67 | H | 51.06337600 | 63.90353200 | 58.62923800 |
| 6 | C | 53.16219100 | 67.29094900 | 54.39002600 | 68 | C | 58.67710200 | 66.46417900 | 55.72189100 |
| 7 | H | 52.81160900 | 68.30536500 | 54.16750800 | 69 | H | 59.45819600 | 67.12453500 | 56.12166300 |
| 8 | H | 53.71836700 | 67.33662900 | 55.33315200 | 70 | H | 59.14972400 | 65.48512100 | 55.60749300 |
| 9 | H | 53.87019800 | 67.02046100 | 53.60612400 | 71 | C | 57.55254500 | 66.38039100 | 56.72791100 |
| 10 | C | 52.49351200 | 64.89489600 | 54.77400600 | 72 | C | 56.50317700 | 67.31092800 | 56.77544200 |
| 11 | H | 53.02401700 | 64.87929100 | 55.73160300 | 73 | H | 56.43969400 | 68.11423900 | 56.04587000 |
| 12 | H | 51.67618400 | 64.17133300 | 54.83207300 | 74 | C | 55.49782600 | 67.21510900 | 57.73699100 |
| 13 | H | 53.19853200 | 64.55357000 | 54.01058500 | 75 | H | 54.67407100 | 67.92292600 | 57.72711000 |
| 14 | C | 48.75826800 | 65.45878900 | 61.37795400 | 76 | C | 55.52969000 | 66.18837500 | 58.68177900 |
| 15 | H | 48.24697300 | 66.36298400 | 61.70345600 | 77 | H | 54.72907200 | 66.10009800 | 59.41083100 |
| 16 | H | 49.21589200 | 65.68933300 | 60.40977200 | 78 | C | 56.57273500 | 65.26175900 | 58.65770700 |
| 17 | C | 49.91123000 | 65.20004000 | 62.30700100 | 79 | H | 56.59842900 | 64.44245400 | 59.37268500 |
| 18 | O | 50.58635400 | 64.01070600 | 62.22709100 | 80 | C | 57.56559900 | 65.35620000 | 57.68421600 |
| 19 | O | 50.36278800 | 65.98619600 | 63.11282200 | 81 | H | 58.35251400 | 64.61020300 | 57.64817000 |
| 20 | C | 49.05835000 | 64.43956000 | 65.91673800 | 82 | C | 56.45698100 | 68.67158500 | 62.74821900 |
| 21 | H | 48.58414600 | 64.56255000 | 64.93544300 | 83 | H | 57.21673400 | 69.09842300 | 62.08889100 |
| 22 | C | 50.34884800 | 63.63478400 | 65.70170500 | 84 | C | 55.95700500 | 67.38259400 | 62.08422700 |
| 23 | H | 51.06337800 | 64.17806300 | 65.07818900 | 85 | H | 56.77327200 | 66.70903500 | 61.80281100 |
| 24 | H | 50.82642800 | 63.37156800 | 66.65177300 | 86 | H | 55.26255600 | 66.84220300 | 62.73562600 |
| 25 | H | 50.11790800 | 62.68975000 | 65.19638100 | 87 | H | 55.43187800 | 67.61139700 | 61.15340300 |
| 26 | C | 48.11839200 | 63.64481700 | 66.81426700 | 88 | C | 55.32461100 | 69.71621300 | 62.90041300 |
| 27 | H | 47.72242600 | 62.78573600 | 66.26766400 | 89 | H | 55.68845100 | 70.58051000 | 63.46576500 |
| 28 | H | 48.64686900 | 63.22306100 | 67.67085600 | 90 | H | 54.51375800 | 69.28966900 | 63.50070500 |
| 29 | H | 47.28655100 | 64.23881300 | 67.18031900 | 91 | C | 54.75789300 | 70.23572200 | 61.57439300 |
| 30 | C | 51.70787000 | 68.54008800 | 64.15637500 | 92 | H | 54.00241900 | 71.00855900 | 61.75259100 |
| 31 | H | 52.08402800 | 68.50933500 | 63.12599900 | 93 | H | 55.54606000 | 70.68200900 | 60.96068400 |
| 32 | H | 50.87117400 | 67.84295300 | 64.18958100 | 94 | H | 54.28787000 | 69.44889900 | 60.97468500 |
| 33 | C | 52.80276000 | 68.06766100 | 65.05801600 | 95 | C | 57.45418600 | 62.81605200 | 63.81238200 |
| 34 | N | 53.53159900 | 66.93640400 | 64.72642900 | 96 | H | 57.40626200 | 61.89843100 | 63.21890200 |
| 35 | C | 54.43993800 | 66.77212700 | 65.67054800 | 97 | H | 58.44381500 | 63.23262000 | 63.62690900 |
| 36 | H | 55.16843600 | 65.97538300 | 65.69760400 | 98 | C | 56.34908800 | 63.77946800 | 63.33707300 |
| 37 | N | 54.32040300 | 67.72789000 | 66.61763000 | 99 | H | 56.75013700 | 64.43718100 | 62.55981100 |
| 38 | C | 53.28731700 | 68.56074500 | 66.24443900 | 100 | H | 56.02503400 | 64.44642100 | 64.14533200 |
| 39 | H | 53.00820200 | 69.41163400 | 66.84656600 | 101 | C | 55.12766900 | 63.05593700 | 62.76096800 |
| 40 | C | 48.76819000 | 72.85442300 | 58.57616600 | 102 | H | 54.71521900 | 62.34537800 | 63.49018800 |
| 41 | H | 48.51481900 | 73.11933200 | 57.54421400 | 103 | H | 55.44068200 | 62.44974800 | 61.90755700 |
| 42 | H | 47.81418600 | 72.70606000 | 59.08757700 | 104 | C | 54.00671100 | 63.96322700 | 62.26164200 |
| 43 | C | 49.57615800 | 71.57573100 | 58.54483000 | 105 | H | 53.30590900 | 63.39528800 | 61.64743200 |
| 44 | C | 49.98181100 | 71.04673800 | 57.29446600 | 106 | H | 54.38968200 | 64.79150800 | 61.66199600 |
| 45 | H | 49.63665500 | 71.58101100 | 56.42530400 | 107 | N | 53.19579100 | 64.55692600 | 63.38801200 |
| 46 | C | 50.92970400 | 70.01449500 | 57.19855400 | 108 | H | 53.41390900 | 65.56111100 | 63.69514700 |
| 47 | H | 51.29735400 | 69.68562100 | 56.23928900 | 109 | H | 52.19862300 | 64.53646200 | 63.13833400 |
| 48 | C | 51.36181400 | 69.36935100 | 58.33556800 | 110 | H | 53.33227200 | 63.99672600 | 64.24461800 |
| 49 | O | 52.27563800 | 68.39900300 | 58.32935600 | 111 | O | 50.99242000 | 67.23145500 | 60.21409000 |
| 50 | H | 52.05878000 | 67.82944800 | 59.12662300 | 112 | H | 51.14528800 | 67.29527300 | 61.16689900 |
| 51 | C | 50.81097400 | 69.70469200 | 59.69253600 | 113 | H | 54.88929300 | 67.81001100 | 67.46000100 |
| 52 | H | 51.64675900 | 69.83091100 | 60.40692200 | 114 | O | 49.96212000 | 68.64479700 | 60.14445000 |
| 53 | C | 49.92418100 | 70.89354900 | 59.67621800 | 115 | H | 50.29266800 | 63.41491900 | 61.48111400 |
| 54 | H | 49.51694300 | 71.23218500 | 60.60389100 | 116 | H | 49.23698353 | 73.72247045 | 58.99044398 |
| 55 | C | 54.15499200 | 62.84104000 | 57.37767600 | 117 | H | 48.06672782 | 64.64760526 | 61.28493187 |
| 56 | H | 54.42707800 | 63.89751800 | 57.29530300 | 118 | H | 56.92428460 | 68.50287672 | 63.69588184 |
| 57 | H | 54.72309700 | 62.45089600 | 58.23137200 | 119 | H | 57.42221834 | 62.53279460 | 64.84371285 |
| 58 | C | 52.63179800 | 62.74051400 | 57.62852500 | 120 | H | 50.04886489 | 66.36282772 | 55.60492053 |
| 59 | H | 52.11927600 | 62.84013800 | 56.66493800 | 121 | H | 58.43307070 | 66.82674074 | 54.74521413 |
| 60 | C | 52.17804900 | 61.41423900 | 58.25308000 | 122 | H | 51.33499958 | 69.52926859 | 64.32187249 |
| 61 | H | 51.08753500 | 61.35351100 | 58.25915800 | 123 | H | 49.28116332 | 65.43249458 | 66.24739665 |
| 62 | H | 52.53364200 | 60.53629400 | 57.71428000 | 124 | H | 54.50427134 | 62.36726432 | 56.48412099 |

| **IM2** | | | | | | | | | |
| --- | --- | --- | --- | --- | --- | --- | --- | --- | --- |
| 1 | C | 51.03587200 | 66.98109800 | 55.83970000 | 63 | H | 52.77190100 | 61.45689900 | 59.46169700 |
| 2 | H | 51.49614800 | 66.83945200 | 56.82454900 | 64 | C | 52.06314000 | 63.93998100 | 58.45340700 |
| 3 | H | 50.83797000 | 68.05430600 | 55.74163900 | 65 | H | 52.49527300 | 64.00614400 | 59.46029200 |
| 4 | C | 52.02614900 | 66.53046400 | 54.74083700 | 66 | H | 52.27712600 | 64.88054400 | 57.94311900 |
| 5 | H | 51.54209700 | 66.60675800 | 53.76101200 | 67 | H | 50.97658500 | 63.86526400 | 58.58134500 |
| 6 | C | 53.23402400 | 67.46666200 | 54.72826200 | 68 | C | 58.88296700 | 66.39734100 | 55.74396700 |
| 7 | H | 52.94754800 | 68.49169600 | 54.47984200 | 69 | H | 59.67894700 | 67.04017600 | 56.14404300 |
| 8 | H | 53.74212700 | 67.47845500 | 55.69912400 | 70 | H | 59.34811200 | 65.42447100 | 55.56662900 |
| 9 | H | 53.97601600 | 67.15563000 | 53.98715200 | 71 | C | 57.78417400 | 66.26993400 | 56.76659400 |
| 10 | C | 52.48660400 | 65.07461800 | 54.88870800 | 72 | C | 56.76146300 | 67.22176200 | 56.87608800 |
| 11 | H | 53.08902300 | 64.95338200 | 55.79455600 | 73 | H | 56.72962900 | 68.07937700 | 56.21018400 |
| 12 | H | 51.65691700 | 64.36503500 | 54.93900700 | 74 | C | 55.75056800 | 67.08407500 | 57.82038500 |
| 13 | H | 53.12320100 | 64.77997000 | 54.04797700 | 75 | H | 54.97278200 | 67.83192500 | 57.87609900 |
| 14 | C | 48.29300100 | 65.85718400 | 61.49326000 | 76 | C | 55.74214000 | 65.98762400 | 58.68069300 |
| 15 | H | 48.08485500 | 66.21714800 | 62.50843200 | 77 | H | 54.92763600 | 65.86729300 | 59.38549500 |
| 16 | H | 47.76328100 | 66.52090900 | 60.80981800 | 78 | C | 56.76496500 | 65.04390100 | 58.60370400 |
| 17 | C | 49.75925300 | 66.09691200 | 61.30474800 | 79 | H | 56.76815900 | 64.17663500 | 59.25943700 |
| 18 | O | 50.66932600 | 65.09994100 | 61.43913100 | 80 | C | 57.77428400 | 65.18732300 | 57.65353400 |
| 19 | O | 50.17756200 | 67.22326100 | 61.12822700 | 81 | H | 58.55550300 | 64.43738500 | 57.58709000 |
| 20 | C | 48.87524600 | 64.48828400 | 65.76324400 | 82 | C | 56.50712700 | 68.74935000 | 62.80873200 |
| 21 | H | 48.11986100 | 64.66958600 | 64.98725100 | 83 | H | 57.27456600 | 69.22149500 | 62.19015700 |
| 22 | C | 50.05440400 | 63.78973100 | 65.07366100 | 84 | C | 56.03202900 | 67.50834000 | 62.05379500 |
| 23 | H | 50.51983600 | 64.39770600 | 64.28669300 | 85 | H | 56.85912000 | 66.84499600 | 61.77631200 |
| 24 | H | 50.83084000 | 63.49502800 | 65.78522900 | 86 | H | 55.30945100 | 66.94419700 | 62.64682500 |
| 25 | H | 49.71074400 | 62.86112000 | 64.60436500 | 87 | H | 55.55069600 | 67.79428900 | 61.11699000 |
| 26 | C | 48.26505000 | 63.56072400 | 66.80813900 | 88 | C | 55.37882300 | 69.78705800 | 63.03179100 |
| 27 | H | 47.82672400 | 62.68504700 | 66.32162500 | 89 | H | 55.77718700 | 70.63781300 | 63.59469500 |
| 28 | H | 49.02500500 | 63.17020700 | 67.49110200 | 90 | H | 54.59662900 | 69.34921400 | 63.66204100 |
| 29 | H | 47.48661100 | 64.04555200 | 67.39203400 | 91 | C | 54.74248100 | 70.32874500 | 61.75061500 |
| 30 | C | 51.67640600 | 68.34382700 | 63.92659400 | 92 | H | 54.07073500 | 71.16409600 | 61.97767700 |
| 31 | H | 52.06937700 | 68.24157200 | 62.91076300 | 93 | H | 55.50542300 | 70.69489600 | 61.05619000 |
| 32 | H | 50.81524900 | 67.67032900 | 63.98753100 | 94 | H | 54.16186400 | 69.56592000 | 61.23089800 |
| 33 | C | 52.74908700 | 67.89716400 | 64.88462900 | 95 | C | 57.43809600 | 62.80248600 | 63.85636400 |
| 34 | N | 53.51037700 | 66.77001000 | 64.60287600 | 96 | H | 57.33613500 | 61.88278800 | 63.27321900 |
| 35 | C | 54.39096700 | 66.65014200 | 65.57865300 | 97 | H | 58.44602600 | 63.16699500 | 63.65230300 |
| 36 | H | 55.13338800 | 65.86974300 | 65.65463300 | 98 | C | 56.38343900 | 63.82475000 | 63.38241000 |
| 37 | N | 54.22246400 | 67.62332400 | 66.50403200 | 99 | H | 56.87525000 | 64.55207500 | 62.72914400 |
| 38 | C | 53.18502400 | 68.42572000 | 66.07694600 | 100 | H | 55.97925800 | 64.40820400 | 64.21685100 |
| 39 | H | 52.87040400 | 69.27652200 | 66.66197400 | 101 | C | 55.22269500 | 63.20156200 | 62.59296800 |
| 40 | C | 49.17334900 | 72.63427900 | 58.23038600 | 102 | H | 54.50427700 | 62.69451800 | 63.24525100 |
| 41 | H | 48.92541000 | 72.80675800 | 57.17522300 | 103 | H | 55.62318300 | 62.41503900 | 61.95023400 |
| 42 | H | 48.29706400 | 72.19685200 | 58.71088100 | 104 | C | 54.52446100 | 64.17556300 | 61.65074500 |
| 43 | C | 50.30829700 | 71.66478200 | 58.34497500 | 105 | H | 54.00442100 | 63.64023400 | 60.85445300 |
| 44 | C | 51.64791800 | 72.03602800 | 57.89392100 | 106 | H | 55.26388900 | 64.82370000 | 61.18000500 |
| 45 | H | 51.78618600 | 72.99079200 | 57.39275900 | 107 | N | 53.49732600 | 65.08703600 | 62.26430800 |
| 46 | C | 52.73681500 | 71.24412200 | 58.03533700 | 108 | H | 53.71648400 | 65.55094600 | 63.17949200 |
| 47 | H | 53.71797000 | 71.54108800 | 57.67839600 | 109 | H | 53.29090200 | 65.88160900 | 61.59842400 |
| 48 | C | 52.64195600 | 69.92317900 | 58.62717500 | 110 | H | 52.61502200 | 64.58789200 | 62.39718800 |
| 49 | O | 53.59495200 | 69.17824000 | 58.79224300 | 111 | O | 52.91568100 | 67.27347500 | 60.76989300 |
| 50 | H | 53.30011400 | 67.66540800 | 59.96314500 | 112 | H | 51.96287800 | 67.18082500 | 60.58261000 |
| 51 | C | 51.21132700 | 69.47388200 | 59.11620900 | 113 | H | 54.75799000 | 67.73108900 | 67.36726900 |
| 52 | H | 51.40992700 | 69.46623800 | 60.21510300 | 114 | O | 50.96200200 | 68.19359100 | 58.75627600 |
| 53 | C | 50.10765000 | 70.44121000 | 58.86322600 | 115 | H | 50.31252300 | 64.17887400 | 61.53856300 |
| 54 | H | 49.11620500 | 70.10765200 | 59.14973600 | 116 | H | 49.33614012 | 73.58747848 | 58.68843600 |
| 55 | C | 54.13117500 | 62.87159000 | 57.40772900 | 117 | H | 49.16677095 | 65.45335686 | 66.12177931 |
| 56 | H | 54.37258800 | 63.93243500 | 57.30157700 | 118 | H | 56.95652231 | 68.53270913 | 63.75531036 |
| 57 | H | 54.73300900 | 62.50836400 | 58.25011900 | 119 | H | 58.59733819 | 66.80324260 | 54.79604291 |
| 58 | C | 52.61551500 | 62.72062700 | 57.69902900 | 120 | H | 57.41014282 | 62.52470051 | 64.88929858 |
| 59 | H | 52.08237000 | 62.68016600 | 56.74128000 | 121 | H | 51.32339373 | 69.34792692 | 64.03643795 |
| 60 | C | 52.26503000 | 61.45480600 | 58.48720700 | 122 | H | 54.47285339 | 62.39148143 | 56.51461555 |
| 61 | H | 51.18946100 | 61.40488000 | 58.67644200 | 123 | H | 47.89928035 | 64.86544700 | 61.41362473 |
| 62 | H | 52.54876700 | 60.53293700 | 57.97595200 | 124 | H | 50.09131862 | 66.47842780 | 55.84614787 |

| **TS3** | | | | | | | | | |
| --- | --- | --- | --- | --- | --- | --- | --- | --- | --- |
| 1 | C | 51.04596600 | 66.99318300 | 55.78582600 | 63 | H | 52.57893400 | 61.37150300 | 59.26961500 |
| 2 | H | 51.51672300 | 66.86906300 | 56.76943800 | 64 | C | 52.06458700 | 63.90684800 | 58.39277100 |
| 3 | H | 50.86825000 | 68.06835200 | 55.66651400 | 65 | H | 52.47060900 | 63.89040600 | 59.41349200 |
| 4 | C | 52.01792700 | 66.50853500 | 54.68903400 | 66 | H | 52.31761700 | 64.87779700 | 57.96249500 |
| 5 | H | 51.53037200 | 66.57551000 | 53.71046800 | 67 | H | 50.97310200 | 63.85738000 | 58.48897900 |
| 6 | C | 53.23896300 | 67.42505100 | 54.66028200 | 68 | C | 58.83204300 | 66.42038300 | 55.74301100 |
| 7 | H | 52.95953000 | 68.46257400 | 54.45463200 | 69 | H | 59.61911900 | 67.07745600 | 56.13724700 |
| 8 | H | 53.77544800 | 67.40499700 | 55.61604800 | 70 | H | 59.30693600 | 65.44836400 | 55.58767500 |
| 9 | H | 53.95270700 | 67.12470400 | 53.88883700 | 71 | C | 57.72953800 | 66.30238600 | 56.76474400 |
| 10 | C | 52.45544600 | 65.04825100 | 54.85402500 | 72 | C | 56.71194400 | 67.26062700 | 56.87555400 |
| 11 | H | 53.03889500 | 64.92835700 | 55.77227600 | 73 | H | 56.68209100 | 68.11616600 | 56.20637200 |
| 12 | H | 51.61002700 | 64.35640100 | 54.89650800 | 74 | C | 55.70214100 | 67.13030000 | 57.82407900 |
| 13 | H | 53.10223300 | 64.73784500 | 54.02808100 | 75 | H | 54.92468700 | 67.88135000 | 57.88330900 |
| 14 | C | 48.29949400 | 65.87205200 | 61.45958700 | 76 | C | 55.69030700 | 66.03452000 | 58.68648700 |
| 15 | H | 48.11146000 | 66.21164500 | 62.48567400 | 77 | H | 54.87658600 | 65.92098600 | 59.39453700 |
| 16 | H | 47.76389300 | 66.55907600 | 60.80411500 | 78 | C | 56.70755400 | 65.08504500 | 58.60655600 |
| 17 | C | 49.76380400 | 66.11776900 | 61.25667100 | 79 | H | 56.70414600 | 64.21759800 | 59.26173400 |
| 18 | O | 50.68672900 | 65.12954300 | 61.28310100 | 80 | C | 57.71498200 | 65.22066600 | 57.65324900 |
| 19 | O | 50.17926300 | 67.25229500 | 61.16866300 | 81 | H | 58.48676900 | 64.46194800 | 57.58148800 |
| 20 | C | 48.81946800 | 64.48804900 | 65.77910900 | 82 | C | 56.46414400 | 68.73727200 | 62.81193000 |
| 21 | H | 47.99346300 | 64.67446200 | 65.08239500 | 83 | H | 57.20406700 | 69.22386000 | 62.17146300 |
| 22 | C | 49.95640500 | 63.87513800 | 64.95256600 | 84 | C | 55.98809400 | 67.49047200 | 62.06503400 |
| 23 | H | 50.28710800 | 64.52535200 | 64.13034900 | 85 | H | 56.81611200 | 66.83357000 | 61.77592800 |
| 24 | H | 50.82958000 | 63.64246500 | 65.56829900 | 86 | H | 55.27141800 | 66.91875700 | 62.66049500 |
| 25 | H | 49.63541600 | 62.92898200 | 64.50478600 | 87 | H | 55.49270200 | 67.77661700 | 61.13584500 |
| 26 | C | 48.32945700 | 63.49934400 | 66.82823600 | 88 | C | 55.32667000 | 69.75640700 | 63.06795700 |
| 27 | H | 47.90689200 | 62.61503400 | 66.34314600 | 89 | H | 55.72442800 | 70.60222200 | 63.63803800 |
| 28 | H | 49.14917400 | 63.13904200 | 67.45611200 | 90 | H | 54.56083400 | 69.29857400 | 63.70445200 |
| 29 | H | 47.56057600 | 63.92908300 | 67.46425400 | 91 | C | 54.66163000 | 70.31062700 | 61.80711600 |
| 30 | C | 51.73203800 | 68.08948300 | 63.94871500 | 92 | H | 53.97765600 | 71.12865500 | 62.05983700 |
| 31 | H | 52.12338900 | 67.95248800 | 62.93600600 | 93 | H | 55.40491700 | 70.70421700 | 61.10593000 |
| 32 | H | 50.88209700 | 67.40330600 | 64.03770900 | 94 | H | 54.08610500 | 69.54862600 | 61.28024800 |
| 33 | C | 52.81336400 | 67.69686500 | 64.90318700 | 95 | C | 57.50155000 | 62.79223200 | 63.81117600 |
| 34 | N | 53.58131300 | 66.57384400 | 64.63523300 | 96 | H | 57.44260400 | 61.86485100 | 63.23330500 |
| 35 | C | 54.45576800 | 66.47007400 | 65.61608700 | 97 | H | 58.50196100 | 63.18523100 | 63.62625200 |
| 36 | H | 55.19113800 | 65.68635900 | 65.70536400 | 98 | C | 56.42978900 | 63.77456800 | 63.29438100 |
| 37 | N | 54.28603100 | 67.46287000 | 66.52074500 | 99 | H | 56.90079700 | 64.46530600 | 62.58788500 |
| 38 | C | 53.24848800 | 68.25527700 | 66.07979100 | 100 | H | 56.03096100 | 64.40230000 | 64.09753400 |
| 39 | H | 52.92772200 | 69.11923400 | 66.64030500 | 101 | C | 55.26194600 | 63.09226700 | 62.57142400 |
| 40 | C | 49.18255200 | 72.76494100 | 58.15268300 | 102 | H | 54.60891300 | 62.56012000 | 63.26927700 |
| 41 | H | 48.94984000 | 72.94550100 | 57.09538200 | 103 | H | 55.66238600 | 62.31936100 | 61.91401500 |
| 42 | H | 48.32007400 | 72.27016500 | 58.59998800 | 104 | C | 54.46035800 | 64.01345400 | 61.65814200 |
| 43 | C | 50.35758600 | 71.82967700 | 58.26124400 | 105 | H | 53.92845600 | 63.43739800 | 60.89793400 |
| 44 | C | 51.71209600 | 72.28393800 | 57.99325800 | 106 | H | 55.12810800 | 64.70032200 | 61.13835200 |
| 45 | H | 51.86520300 | 73.28634500 | 57.60779500 | 107 | N | 53.40933100 | 64.86542800 | 62.31693900 |
| 46 | C | 52.80287000 | 71.49790400 | 58.18716000 | 108 | H | 53.65090200 | 65.36667500 | 63.21503800 |
| 47 | H | 53.80885600 | 71.84993500 | 57.98454400 | 109 | H | 53.13915100 | 65.62810000 | 61.64956100 |
| 48 | C | 52.67091600 | 70.11526300 | 58.62199800 | 110 | H | 52.57198000 | 64.30920400 | 62.50789800 |
| 49 | O | 53.63765600 | 69.37558700 | 58.83669400 | 111 | O | 52.93986900 | 67.13613100 | 60.76783000 |
| 50 | H | 53.28836900 | 67.60695300 | 59.99001600 | 112 | H | 51.97210800 | 67.14669400 | 60.64291500 |
| 51 | C | 51.25865900 | 69.60746900 | 58.79057900 | 113 | H | 54.82953200 | 67.60370200 | 67.37187000 |
| 52 | H | 51.12894600 | 69.04274800 | 59.88069900 | 114 | O | 51.07022600 | 68.25077000 | 58.76683900 |
| 53 | C | 50.15602200 | 70.51909200 | 58.58896200 | 115 | H | 50.35197200 | 64.19804300 | 61.32448400 |
| 54 | H | 49.16438200 | 70.11414300 | 58.75790100 | 116 | H | 49.27688182 | 73.71785030 | 58.63014059 |
| 55 | C | 54.13202700 | 62.85140400 | 57.34931200 | 117 | H | 47.89606119 | 64.88586616 | 61.36171233 |
| 56 | H | 54.39396200 | 63.90979800 | 57.26577000 | 118 | H | 51.35429293 | 69.08885713 | 64.00753683 |
| 57 | H | 54.68921100 | 62.47101700 | 58.21472600 | 119 | H | 49.11840522 | 65.44468938 | 66.15377648 |
| 58 | C | 52.60613900 | 62.72843600 | 57.57188400 | 120 | H | 57.45618044 | 62.52555549 | 64.84641763 |
| 59 | H | 52.11223500 | 62.77389700 | 56.59374600 | 121 | H | 58.55188170 | 66.80665308 | 54.78530276 |
| 60 | C | 52.17758800 | 61.42506100 | 58.24828700 | 122 | H | 50.08933011 | 66.51514425 | 55.82085071 |
| 61 | H | 51.08971300 | 61.38604300 | 58.32041000 | 123 | H | 56.94344276 | 68.52970683 | 63.74578727 |
| 62 | H | 52.50061300 | 60.52614300 | 57.72412400 | 124 | H | 54.50188941 | 62.37246366 | 56.46686203 |

| **PC** | | | | | | | | | |
| --- | --- | --- | --- | --- | --- | --- | --- | --- | --- |
| 1 | C | 51.03142700 | 66.96993300 | 55.79037200 | 63 | H | 52.73719000 | 61.45585800 | 59.44205900 |
| 2 | H | 51.49007400 | 66.84055900 | 56.77795700 | 64 | C | 52.03636000 | 63.91593500 | 58.40113300 |
| 3 | H | 50.83050200 | 68.04144600 | 55.68363100 | 65 | H | 52.49496500 | 64.01334000 | 59.39554000 |
| 4 | C | 52.02609100 | 66.51411900 | 54.69826000 | 66 | H | 52.22381000 | 64.84994700 | 57.86976800 |
| 5 | H | 51.53610700 | 66.55393500 | 53.71914700 | 67 | H | 50.95425400 | 63.82611700 | 58.55298200 |
| 6 | C | 53.21229800 | 67.47656500 | 54.65904000 | 68 | C | 58.84367500 | 66.40190900 | 55.74548600 |
| 7 | H | 52.90268400 | 68.48800100 | 54.38459200 | 69 | H | 59.62272800 | 67.05925800 | 56.15483600 |
| 8 | H | 53.71868300 | 67.52762700 | 55.62933900 | 70 | H | 59.33104600 | 65.43890000 | 55.57208400 |
| 9 | H | 53.96130300 | 67.16076900 | 53.92706200 | 71 | C | 57.74192300 | 66.24503800 | 56.76324600 |
| 10 | C | 52.51980200 | 65.07288500 | 54.87956600 | 72 | C | 56.67903100 | 67.15356300 | 56.86377800 |
| 11 | H | 53.14616700 | 64.99330600 | 55.77411300 | 73 | H | 56.61177800 | 68.00155300 | 56.18798300 |
| 12 | H | 51.70803100 | 64.34615500 | 54.97036400 | 74 | C | 55.67149800 | 66.97936100 | 57.80868200 |
| 13 | H | 53.14381700 | 64.76491500 | 54.03446000 | 75 | H | 54.85903400 | 67.69204100 | 57.86764000 |
| 14 | C | 48.20720100 | 65.94743000 | 61.50282900 | 76 | C | 55.71356800 | 65.88999600 | 58.67739600 |
| 15 | H | 48.32891200 | 66.14938500 | 62.57493400 | 77 | H | 54.89901000 | 65.73858500 | 59.37700100 |
| 16 | H | 47.42555800 | 66.60858500 | 61.13639200 | 78 | C | 56.77643700 | 64.99049600 | 58.61088400 |
| 17 | C | 49.45485600 | 66.41302000 | 60.82584100 | 79 | H | 56.81258000 | 64.12972600 | 59.27361200 |
| 18 | O | 50.56908500 | 65.66104400 | 60.80381100 | 80 | C | 57.77847200 | 65.16965900 | 57.65998000 |
| 19 | O | 49.49359700 | 67.54098500 | 60.36071600 | 81 | H | 58.59000400 | 64.45245400 | 57.59554900 |
| 20 | C | 48.84370000 | 64.52989600 | 65.76858800 | 82 | C | 56.54282500 | 68.76494500 | 62.78033800 |
| 21 | H | 48.05931900 | 64.71432600 | 65.02364400 | 83 | H | 57.31311600 | 69.27466400 | 62.19821100 |
| 22 | C | 50.01543000 | 63.88148300 | 65.02244100 | 84 | C | 56.13301600 | 67.54441400 | 61.95607400 |
| 23 | H | 50.43671100 | 64.52969100 | 64.23943200 | 85 | H | 56.95959100 | 66.85273900 | 61.76246100 |
| 24 | H | 50.82673500 | 63.60549100 | 65.70234900 | 86 | H | 55.31990100 | 66.98978900 | 62.43486700 |
| 25 | H | 49.68917800 | 62.95490200 | 64.53841500 | 87 | H | 55.81744500 | 67.87770400 | 60.96156800 |
| 26 | C | 48.28854800 | 63.56206900 | 66.80653500 | 88 | C | 55.38491800 | 69.77073700 | 62.99517200 |
| 27 | H | 47.86448200 | 62.68410800 | 66.31138000 | 89 | H | 55.75188800 | 70.60727300 | 63.59918900 |
| 28 | H | 49.07487000 | 63.18325900 | 67.46573700 | 90 | H | 54.59244000 | 69.29826800 | 63.58678000 |
| 29 | H | 47.50824800 | 64.01166100 | 67.41488300 | 91 | C | 54.78258100 | 70.34839600 | 61.71364500 |
| 30 | C | 51.63770900 | 68.33660900 | 63.84252000 | 92 | H | 54.10496000 | 71.17653000 | 61.94656200 |
| 31 | H | 52.02007000 | 68.25316700 | 62.81906100 | 93 | H | 55.56076300 | 70.73245100 | 61.04716600 |
| 32 | H | 50.77428900 | 67.66386400 | 63.90452300 | 94 | H | 54.20974400 | 69.61430800 | 61.14628500 |
| 33 | C | 52.71641500 | 67.86782400 | 64.78262100 | 95 | C | 57.46879900 | 62.80331500 | 63.80008800 |
| 34 | N | 53.45777100 | 66.72599200 | 64.50254600 | 96 | H | 57.40006300 | 61.88254400 | 63.21376900 |
| 35 | C | 54.34122300 | 66.59761000 | 65.47620500 | 97 | H | 58.46917200 | 63.19383000 | 63.61005100 |
| 36 | H | 55.06838800 | 65.80395700 | 65.55881200 | 98 | C | 56.39598300 | 63.79673800 | 63.30197400 |
| 37 | N | 54.19985600 | 67.58421400 | 66.39156000 | 99 | H | 56.86084700 | 64.47548500 | 62.57960100 |
| 38 | C | 53.17502200 | 68.40101400 | 65.96429300 | 100 | H | 56.02503100 | 64.43552700 | 64.11022500 |
| 39 | H | 52.88088300 | 69.26491800 | 66.54039700 | 101 | C | 55.20486500 | 63.12204700 | 62.60761400 |
| 40 | C | 49.35280700 | 72.56476300 | 58.26364300 | 102 | H | 54.56343700 | 62.59842500 | 63.32178000 |
| 41 | H | 49.00789200 | 72.71639200 | 57.23154200 | 103 | H | 55.58995400 | 62.34169000 | 61.94917200 |
| 42 | H | 48.55254900 | 72.04856100 | 58.79823500 | 104 | C | 54.39291100 | 64.03417300 | 61.69063700 |
| 43 | C | 50.58394500 | 71.70974300 | 58.25125600 | 105 | H | 53.89365400 | 63.44808400 | 60.91654900 |
| 44 | C | 51.85478800 | 72.23792800 | 57.87824700 | 106 | H | 55.05301400 | 64.73986900 | 61.18736500 |
| 45 | H | 51.94051500 | 73.24691900 | 57.49034900 | 107 | N | 53.29685300 | 64.85879900 | 62.31926700 |
| 46 | C | 52.99321300 | 71.48992600 | 58.00184700 | 108 | H | 53.50137900 | 65.39319000 | 63.20835000 |
| 47 | H | 53.97136900 | 71.89243600 | 57.75995900 | 109 | H | 53.03092300 | 65.62515500 | 61.64178900 |
| 48 | C | 52.94592600 | 70.12302000 | 58.46098400 | 110 | H | 52.47778200 | 64.26479500 | 62.49508800 |
| 49 | O | 53.97309800 | 69.44400500 | 58.70306300 | 111 | O | 53.16395100 | 67.13300900 | 60.85521500 |
| 50 | H | 52.67369000 | 67.42775600 | 60.05905100 | 112 | H | 54.06904100 | 67.43390700 | 60.70304800 |
| 51 | C | 51.61322600 | 69.56643900 | 58.68156400 | 113 | H | 54.74713200 | 67.69735100 | 67.24667900 |
| 52 | H | 50.70637400 | 67.99599100 | 59.38046000 | 114 | O | 51.57079900 | 68.25302300 | 58.97113200 |
| 53 | C | 50.48274300 | 70.36501700 | 58.62324400 | 115 | H | 50.45151400 | 64.74502200 | 61.15287100 |
| 54 | H | 49.51551700 | 69.93912200 | 58.87430300 | 116 | H | 49.47226127 | 73.52821733 | 58.71351679 |
| 55 | C | 54.11389600 | 62.85251700 | 57.38235800 | 117 | H | 49.13638219 | 65.49054027 | 66.13791132 |
| 56 | H | 54.34951800 | 63.91446000 | 57.27843000 | 118 | H | 57.43369182 | 62.52569064 | 64.83284751 |
| 57 | H | 54.70978900 | 62.49443300 | 58.23119800 | 119 | H | 56.97443244 | 68.54035976 | 63.73332094 |
| 58 | C | 52.59768100 | 62.69327300 | 57.66124800 | 120 | H | 51.29187058 | 69.33966274 | 63.98100819 |
| 59 | H | 52.07081700 | 62.64195100 | 56.70049400 | 121 | H | 58.56471603 | 66.80294796 | 54.79351455 |
| 60 | C | 52.24886300 | 61.43305400 | 58.45790800 | 122 | H | 54.46817056 | 62.37389644 | 56.49336312 |
| 61 | H | 51.17125800 | 61.36773000 | 58.62692600 | 123 | H | 47.87923061 | 64.93365578 | 61.40486342 |
| 62 | H | 52.55827800 | 60.50952300 | 57.96516000 | 124 | H | 50.08966771 | 66.46201132 | 55.79261052 |

**Cartesian coordinates for the DOPA radical transfer** **between DOPA126 and Trp52** **in the protonated state of Asp88 (Pathway B).**

| **RC1** | | | | | | | | | |
| --- | --- | --- | --- | --- | --- | --- | --- | --- | --- |
| 1 | C | 53.35026000 | 43.11992900 | 92.20830600 | 87 | H | 44.21840100 | 35.59743200 | 83.92732200 |
| 2 | C | 53.09420800 | 42.07901900 | 91.15702100 | 88 | H | 43.91073000 | 35.85674600 | 82.21837800 |
| 3 | C | 53.19702900 | 40.70868200 | 91.29298300 | 89 | H | 44.89994000 | 33.54657500 | 88.25324900 |
| 4 | C | 52.55085700 | 42.30239900 | 89.84156700 | 90 | H | 44.79730000 | 32.73589800 | 85.92188000 |
| 5 | N | 52.76766800 | 40.06837500 | 90.15306600 | 91 | H | 45.87597100 | 34.02931500 | 85.36455500 |
| 6 | C | 52.34828500 | 41.01902400 | 89.25134900 | 92 | H | 44.23650600 | 34.40326900 | 85.94909700 |
| 7 | C | 52.24288200 | 43.45666000 | 89.09626600 | 93 | H | 46.53782300 | 31.83704300 | 87.42088600 |
| 8 | C | 51.84358700 | 40.88205700 | 87.94979900 | 94 | H | 47.33810400 | 32.83780100 | 88.63717200 |
| 9 | C | 51.73297800 | 43.32029500 | 87.81088600 | 95 | H | 47.65755800 | 33.11740300 | 86.91826500 |
| 10 | C | 51.53429000 | 42.04134300 | 87.24506900 | 96 | H | 48.45520400 | 37.93770200 | 84.43470500 |
| 11 | C | 48.26521200 | 35.94494000 | 77.38188000 | 97 | H | 47.17687100 | 37.21346600 | 85.37469000 |
| 12 | C | 49.35162400 | 35.64569700 | 76.33306400 | 98 | H | 49.59991100 | 38.82127100 | 87.96183500 |
| 13 | C | 50.59949700 | 36.47680400 | 76.66007100 | 99 | H | 51.20387000 | 37.00259100 | 88.77483100 |
| 14 | C | 49.68594500 | 34.15104900 | 76.24820200 | 100 | H | 50.96013100 | 34.88068200 | 87.30919600 |
| 15 | C | 44.52589000 | 35.27891900 | 82.91593300 | 101 | H | 53.23033800 | 30.12048900 | 79.27637000 |
| 16 | C | 45.96963400 | 35.68023800 | 82.77303600 | 102 | H | 51.71151600 | 30.53989500 | 80.07785800 |
| 17 | O | 46.89004900 | 34.87983700 | 82.95140600 | 103 | H | 53.87858900 | 32.59095100 | 79.36981900 |
| 18 | O | 46.12121400 | 36.93665600 | 82.50011200 | 104 | H | 52.52182800 | 34.04044300 | 80.91714800 |
| 19 | C | 45.68222200 | 33.84142900 | 87.53272200 | 105 | H | 51.62742500 | 33.63914500 | 79.43653200 |
| 20 | C | 45.11788800 | 33.76541000 | 86.11813600 | 106 | H | 51.25266300 | 32.79018500 | 80.95298800 |
| 21 | C | 46.86613800 | 32.86756200 | 87.64497900 | 107 | H | 54.37310300 | 32.59483200 | 81.78870500 |
| 22 | C | 48.03763700 | 37.89051700 | 85.45596300 | 108 | H | 53.13881800 | 31.34591200 | 82.07240000 |
| 23 | C | 49.05527000 | 37.30766000 | 86.38717100 | 109 | H | 54.59555600 | 30.95548400 | 81.14164100 |
| 24 | N | 49.51530400 | 36.01971400 | 86.21036600 | 110 | H | 56.98518200 | 35.68541600 | 77.21776200 |
| 25 | C | 49.70557200 | 37.85535200 | 87.47605300 | 111 | H | 55.97381400 | 34.33501500 | 76.77032200 |
| 26 | C | 50.40134000 | 35.80517000 | 87.17136200 | 112 | H | 56.55165600 | 34.53386200 | 79.45473300 |
| 27 | N | 50.54869600 | 36.88715400 | 87.96537000 | 113 | H | 53.50931500 | 36.80006500 | 77.39590700 |
| 28 | C | 46.25653200 | 41.76621400 | 80.35617700 | 114 | H | 55.40879000 | 35.00253700 | 81.61213500 |
| 29 | C | 47.32339000 | 40.70214200 | 80.29132500 | 115 | H | 52.42119100 | 37.31260300 | 79.54477800 |
| 30 | C | 48.42686200 | 40.87315000 | 79.39139400 | 116 | H | 53.41131700 | 36.48201800 | 81.67480100 |
| 31 | C | 47.27571600 | 39.57485700 | 81.09170900 | 117 | H | 53.45830000 | 38.50203100 | 83.53624700 |
| 32 | C | 49.44597400 | 39.95620800 | 79.31075400 | 118 | H | 51.39544900 | 37.19117600 | 82.86094600 |
| 33 | C | 48.32823900 | 38.61590900 | 81.09112800 | 119 | H | 52.59751300 | 36.15759600 | 83.61477700 |
| 34 | C | 49.45970600 | 38.78889300 | 80.14861100 | 120 | H | 51.18665000 | 36.69718200 | 84.54468900 |
| 35 | O | 48.36438300 | 37.60597700 | 81.87961300 | 121 | H | 52.16502800 | 40.32411400 | 84.86780100 |
| 36 | O | 50.38047300 | 37.92732500 | 80.13154700 | 122 | H | 50.81735400 | 39.19754900 | 84.90477800 |
| 37 | C | 52.48251400 | 30.92528900 | 79.39039900 | 123 | H | 50.53640800 | 40.73293300 | 82.96497500 |
| 38 | C | 53.12440200 | 32.15116000 | 80.04620900 | 124 | H | 52.16621900 | 40.35850800 | 82.38223300 |
| 39 | C | 52.07211100 | 33.21577400 | 80.35055600 | 125 | H | 50.89951800 | 39.12965900 | 82.32324400 |
| 40 | C | 53.85021300 | 31.74258900 | 81.32946500 | 126 | H | 57.00471500 | 37.56972500 | 89.18296300 |
| 41 | C | 55.93750100 | 35.41022300 | 77.00149100 | 127 | H | 58.60393600 | 37.64050800 | 88.40207500 |
| 42 | C | 55.14525700 | 35.66527600 | 78.26405900 | 128 | H | 59.47431900 | 36.09532600 | 90.28092900 |
| 43 | C | 53.96277600 | 36.41929000 | 78.31380700 | 129 | H | 57.89242700 | 36.22286000 | 91.05276400 |
| 44 | C | 55.65110500 | 35.15562400 | 79.47395200 | 130 | H | 59.85318300 | 38.52023600 | 90.50156900 |
| 45 | C | 53.33571000 | 36.71379200 | 79.53034700 | 131 | H | 59.61803000 | 37.68675100 | 92.05239200 |
| 46 | C | 55.02403900 | 35.43355100 | 80.68742600 | 132 | H | 57.01767200 | 38.66043400 | 91.28850000 |
| 47 | C | 53.88352200 | 36.24071900 | 80.72362000 | 133 | H | 56.26658800 | 40.56701900 | 92.22702000 |
| 48 | C | 52.67409800 | 38.26772300 | 84.27447000 | 134 | H | 57.33661500 | 41.77153200 | 92.75416700 |
| 49 | C | 51.71495900 | 39.47669700 | 84.32588800 | 135 | H | 60.25142200 | 40.00935900 | 92.29544300 |
| 50 | C | 51.93095500 | 37.01456000 | 83.80250500 | 136 | H | 59.59579000 | 41.52867900 | 92.57268200 |
| 51 | C | 51.30470100 | 39.94760600 | 82.93020800 | 137 | H | 52.23805100 | 34.43410400 | 90.08763800 |
| 52 | C | 57.94063600 | 37.02110500 | 89.02890800 | 138 | H | 52.59219800 | 35.27619800 | 88.56925400 |
| 53 | C | 58.59705100 | 36.75303200 | 90.39192900 | 139 | H | 53.27614200 | 31.43954400 | 84.36091000 |
| 54 | C | 59.10468600 | 37.99863100 | 91.12211900 | 140 | H | 54.31106300 | 32.83038300 | 84.65394400 |
| 55 | N | 58.02994800 | 38.93233700 | 91.45717600 | 141 | H | 52.24831700 | 33.42986700 | 83.48784900 |
| 56 | C | 58.23946400 | 40.15381300 | 91.90986100 | 142 | H | 52.32730000 | 34.28934900 | 85.01052000 |
| 57 | N | 59.49591700 | 40.65068600 | 92.04585000 | 143 | H | 50.50321100 | 33.09945200 | 85.95482600 |
| 58 | N | 57.19469300 | 40.98421400 | 92.11486200 | 144 | H | 50.80911900 | 31.66769200 | 85.00039100 |
| 59 | C | 52.98175900 | 35.06158100 | 89.57015000 | 145 | H | 48.71065700 | 32.68605800 | 84.33706900 |
| 60 | C | 53.10933400 | 36.36500600 | 90.35659600 | 146 | H | 49.84935600 | 32.66430400 | 82.98078100 |
| 61 | O | 53.92896500 | 36.41994600 | 91.31249700 | 147 | H | 48.44567500 | 34.71710800 | 83.39855500 |
| 62 | O | 52.31347400 | 37.29417000 | 90.03923100 | 148 | H | 49.48697700 | 35.09946600 | 84.66559800 |
| 63 | C | 53.35995700 | 32.36449300 | 84.95865700 | 149 | H | 50.02195500 | 34.97105600 | 82.99877300 |
| 64 | C | 52.18994500 | 33.28636700 | 84.58029900 | 150 | H | 45.84389200 | 41.91715200 | 79.34571400 |
| 65 | C | 50.78568400 | 32.76519800 | 84.94276800 | 151 | H | 45.42485500 | 41.44666500 | 81.00235000 |
| 66 | C | 49.65576400 | 33.10947000 | 83.96748700 | 152 | H | 48.45181800 | 41.76396800 | 78.75592700 |
| 67 | N | 49.42436300 | 34.56578700 | 83.75374600 | 153 | H | 46.44209100 | 39.41219900 | 81.77466200 |
| 68 | O | 55.47225200 | 38.43914700 | 90.95169500 | 154 | H | 50.27678400 | 40.08759400 | 78.61444900 |
| 69 | O | 50.23318700 | 35.48521100 | 81.26937400 | 155 | H | 47.16150200 | 37.22014600 | 82.29879000 |
| 70 | H | 54.32537700 | 42.95437000 | 92.70396000 | 156 | H | 55.13307400 | 38.80777300 | 90.11635000 |
| 71 | H | 53.43148100 | 44.11530100 | 91.74256300 | 157 | H | 54.83022300 | 37.67973100 | 91.17288600 |
| 72 | H | 53.55674500 | 40.12604300 | 92.13993500 | 158 | H | 50.06027300 | 36.43802500 | 81.04362300 |
| 73 | H | 52.63599600 | 39.04394700 | 90.07655900 | 159 | H | 51.08825500 | 35.33276500 | 80.83788800 |
| 74 | H | 52.43138600 | 44.44657100 | 89.51558300 | 160 | H | 47.31640541 | 35.47316531 | 77.23323719 |
| 75 | H | 51.49155200 | 44.20761200 | 87.22291100 | 161 | H | 44.33585656 | 34.22962187 | 82.82782613 |
| 76 | H | 51.71041500 | 39.89986400 | 87.49508700 | 162 | H | 47.69024344 | 38.87315222 | 85.69813184 |
| 77 | H | 51.14563100 | 41.95148900 | 86.22859100 | 163 | H | 46.59150166 | 42.72653175 | 80.68857007 |
| 78 | H | 48.63893800 | 35.63683200 | 78.37352700 | 164 | H | 52.02126648 | 31.08161886 | 78.43765995 |
| 79 | H | 48.10622500 | 37.03154200 | 77.45122600 | 165 | H | 55.65202744 | 35.92931369 | 76.11045245 |
| 80 | H | 48.99591900 | 35.96638200 | 75.33857600 | 166 | H | 52.61397184 | 43.18960453 | 92.98156215 |
| 81 | H | 50.51419200 | 33.95860500 | 75.54572900 | 167 | H | 57.71845282 | 36.12466290 | 88.48860605 |
| 82 | H | 48.83152500 | 33.54875900 | 75.89665400 | 168 | H | 53.88482863 | 34.49507189 | 89.47832047 |
| 83 | H | 49.99352800 | 33.75990600 | 77.23159000 | 169 | H | 45.97301642 | 34.84223761 | 87.77504561 |
| 84 | H | 51.39185700 | 36.32859800 | 75.90977800 | 170 | H | 53.46797597 | 32.05213280 | 85.97633230 |
| 85 | H | 50.36407500 | 37.55131300 | 76.69528700 | 171 | H | 53.16778235 | 38.08696193 | 85.20640411 |
| 86 | H | 51.00887200 | 36.19789800 | 77.64274500 |  |  |  |  |  |

| **RC2** | | | | | | | | | |
| --- | --- | --- | --- | --- | --- | --- | --- | --- | --- |
| 1 | C | 53.32729500 | 43.13321800 | 92.16929300 | 87 | H | 43.99697700 | 35.44259200 | 84.00339200 |
| 2 | C | 53.08604600 | 42.08346000 | 91.12321600 | 88 | H | 43.55803200 | 35.74660600 | 82.33965700 |
| 3 | C | 53.23235200 | 40.72365300 | 91.25959600 | 89 | H | 44.90146800 | 33.50307700 | 88.23515200 |
| 4 | C | 52.52437000 | 42.29119000 | 89.81447700 | 90 | H | 44.72586000 | 32.65290400 | 85.93277700 |
| 5 | N | 52.82295200 | 40.06637000 | 90.11512500 | 91 | H | 45.75493700 | 33.95680400 | 85.32765200 |
| 6 | C | 52.35372700 | 41.00752400 | 89.22408200 | 92 | H | 44.13260800 | 34.29866300 | 85.97095000 |
| 7 | C | 52.17891400 | 43.43483700 | 89.07591100 | 93 | H | 46.50499900 | 31.78481900 | 87.38224900 |
| 8 | C | 51.83857800 | 40.85632700 | 87.93235600 | 94 | H | 47.34110200 | 32.80306400 | 88.54388000 |
| 9 | C | 51.65465600 | 43.28639000 | 87.80004500 | 95 | H | 47.60386500 | 33.04853300 | 86.82155700 |
| 10 | C | 51.48342200 | 42.00581600 | 87.23639000 | 96 | H | 48.51289500 | 37.91051400 | 84.36556000 |
| 11 | C | 48.14005300 | 35.96545600 | 77.55079500 | 97 | H | 47.21919800 | 37.19963700 | 85.29927700 |
| 12 | C | 49.24081000 | 35.66804800 | 76.51200900 | 98 | H | 49.66659900 | 38.81455100 | 87.85199700 |
| 13 | C | 50.49492500 | 36.48583900 | 76.85499400 | 99 | H | 51.21545200 | 36.98754200 | 88.73853700 |
| 14 | C | 49.56943300 | 34.17184500 | 76.42057600 | 100 | H | 50.91737300 | 34.83721100 | 87.32287100 |
| 15 | C | 44.23494000 | 35.17197800 | 82.96763700 | 101 | H | 53.13278200 | 30.08344000 | 79.28714700 |
| 16 | C | 45.62267800 | 35.66835900 | 82.72534800 | 102 | H | 51.64557400 | 30.58138400 | 80.08227700 |
| 17 | O | 46.65203500 | 34.78613400 | 82.76899300 | 103 | H | 53.92867000 | 32.47796200 | 79.34766700 |
| 18 | O | 45.86029500 | 36.84547800 | 82.54163800 | 104 | H | 52.63047600 | 34.07399700 | 80.78020100 |
| 19 | C | 45.65434300 | 33.78760600 | 87.49236000 | 105 | H | 51.78413500 | 33.69789600 | 79.27798100 |
| 20 | C | 45.02837300 | 33.68386000 | 86.10373300 | 106 | H | 51.26532200 | 32.92485500 | 80.78560100 |
| 21 | C | 46.84086700 | 32.81143400 | 87.57414600 | 107 | H | 54.33922600 | 32.55093100 | 81.77263000 |
| 22 | C | 48.08218400 | 37.86572100 | 85.37546800 | 108 | H | 52.99926300 | 31.42779100 | 82.06331800 |
| 23 | C | 49.08581200 | 37.28354700 | 86.32567000 | 109 | H | 54.44457900 | 30.87571900 | 81.22165300 |
| 24 | N | 49.51130900 | 35.97407400 | 86.18207300 | 110 | H | 56.97478700 | 35.69669800 | 77.27756000 |
| 25 | C | 49.74385800 | 37.83913200 | 87.39877800 | 111 | H | 55.96449800 | 34.35481300 | 76.83381800 |
| 26 | C | 50.39127100 | 35.76586700 | 87.15730600 | 112 | H | 56.54143500 | 34.50053300 | 79.49903700 |
| 27 | N | 50.56039900 | 36.86363000 | 87.91951100 | 113 | H | 53.53724400 | 36.84957300 | 77.51259800 |
| 28 | C | 46.35388000 | 41.82535400 | 80.24702200 | 114 | H | 55.46437900 | 34.99312800 | 81.67251300 |
| 29 | C | 47.40570300 | 40.75314800 | 80.18504400 | 115 | H | 52.52393800 | 37.39662400 | 79.66848500 |
| 30 | C | 48.58314000 | 40.95604600 | 79.39997800 | 116 | H | 53.53322500 | 36.54529100 | 81.78209600 |
| 31 | C | 47.23972200 | 39.54755600 | 80.87066400 | 117 | H | 53.49118700 | 38.50693000 | 83.53630900 |
| 32 | C | 49.56801400 | 40.00986400 | 79.32571200 | 118 | H | 51.40784100 | 37.22318400 | 82.90179700 |
| 33 | C | 48.24121000 | 38.57944000 | 80.84313400 | 119 | H | 52.58645700 | 36.18021300 | 83.66114400 |
| 34 | C | 49.46348100 | 38.78163300 | 80.06673300 | 120 | H | 51.20493200 | 36.76646000 | 84.59275200 |
| 35 | O | 48.20297100 | 37.43407900 | 81.52178600 | 121 | H | 52.21607700 | 40.36076400 | 84.85178600 |
| 36 | O | 50.37153800 | 37.90577500 | 80.09054600 | 122 | H | 50.86689100 | 39.25164300 | 84.89071000 |
| 37 | C | 52.42369400 | 30.91468600 | 79.38568200 | 123 | H | 50.62075300 | 40.77519400 | 82.93939700 |
| 38 | C | 53.12593800 | 32.12517700 | 80.00754000 | 124 | H | 52.23626700 | 40.37330400 | 82.37420600 |
| 39 | C | 52.13981500 | 33.27343500 | 80.22231500 | 125 | H | 50.95942800 | 39.16885700 | 82.31243500 |
| 40 | C | 53.76711900 | 31.72538300 | 81.33893300 | 126 | H | 57.04460800 | 37.58351500 | 89.20771800 |
| 41 | C | 55.93290400 | 35.42186000 | 77.06940900 | 127 | H | 58.63006000 | 37.65227500 | 88.42307800 |
| 42 | C | 55.15325100 | 35.67174300 | 78.34170200 | 128 | H | 59.51928300 | 36.10313000 | 90.26719200 |
| 43 | C | 53.99362000 | 36.45331400 | 78.41483700 | 129 | H | 57.95780300 | 36.21508200 | 91.06121000 |
| 44 | C | 55.66243100 | 35.13934700 | 79.53776200 | 130 | H | 59.89056400 | 38.52271700 | 90.50958300 |
| 45 | C | 53.40485700 | 36.76430900 | 79.64348700 | 131 | H | 59.68115900 | 37.67782100 | 92.05197800 |
| 46 | C | 55.07106200 | 35.43164300 | 80.76296500 | 132 | H | 57.08103100 | 38.64427300 | 91.33007600 |
| 47 | C | 53.96122500 | 36.27668700 | 80.82431100 | 133 | H | 56.32627900 | 40.55342600 | 92.27165700 |
| 48 | C | 52.71522700 | 38.29768700 | 84.28054600 | 134 | H | 57.39512700 | 41.76001300 | 92.77517500 |
| 49 | C | 51.76592800 | 39.51713500 | 84.31962200 | 135 | H | 60.29515000 | 40.00963200 | 92.31118600 |
| 50 | C | 51.94245100 | 37.04807600 | 83.83786600 | 136 | H | 59.64055600 | 41.52412800 | 92.56647900 |
| 51 | C | 51.37038900 | 39.98110000 | 82.91365000 | 137 | H | 52.24266900 | 34.44582500 | 90.02458900 |
| 52 | C | 57.97267000 | 37.03563700 | 89.04639800 | 138 | H | 52.62955200 | 35.29187400 | 88.52642400 |
| 53 | C | 58.64630900 | 36.75079900 | 90.40008400 | 139 | H | 53.38421200 | 31.50128900 | 84.35197800 |
| 54 | C | 59.16323700 | 37.99187500 | 91.13406200 | 140 | H | 54.39415300 | 32.89117500 | 84.68697400 |
| 55 | N | 58.08120900 | 38.91808600 | 91.48967300 | 141 | H | 52.35313700 | 33.50177900 | 83.49913800 |
| 56 | C | 58.29152600 | 40.14284200 | 91.93468600 | 142 | H | 52.37091300 | 34.30936900 | 85.04814900 |
| 57 | N | 59.54953700 | 40.64821900 | 92.04082800 | 143 | H | 50.57645200 | 33.00647100 | 85.91511900 |
| 58 | N | 57.24584200 | 40.97392700 | 92.14306500 | 144 | H | 50.94475000 | 31.65362700 | 84.87998200 |
| 59 | C | 52.98745500 | 35.08144600 | 89.53367500 | 145 | H | 48.83043200 | 32.62402800 | 84.25440200 |
| 60 | C | 53.06757700 | 36.38393200 | 90.32768700 | 146 | H | 49.98978700 | 32.73782700 | 82.91839600 |
| 61 | O | 53.85878200 | 36.45277600 | 91.30727100 | 147 | H | 48.51665800 | 34.68941300 | 83.45059100 |
| 62 | O | 52.24438500 | 37.29464300 | 89.99242400 | 148 | H | 49.51309700 | 35.08105300 | 84.74791800 |
| 63 | C | 53.45015200 | 32.41068700 | 84.96161100 | 149 | H | 50.05547400 | 35.02191500 | 83.05542800 |
| 64 | C | 52.27441300 | 33.32666300 | 84.57949300 | 150 | H | 45.97089900 | 41.99366900 | 79.23213900 |
| 65 | C | 50.88352500 | 32.74404000 | 84.89689400 | 151 | H | 45.50633400 | 41.49925600 | 80.85547100 |
| 66 | C | 49.75774800 | 33.09840600 | 83.92339800 | 152 | H | 48.69179200 | 41.88831100 | 78.85297900 |
| 67 | N | 49.47297800 | 34.57324900 | 83.79619000 | 153 | H | 46.34487300 | 39.37174000 | 81.45735500 |
| 68 | O | 55.48683600 | 38.46885100 | 90.98265700 | 154 | H | 50.45764500 | 40.15674900 | 78.72573500 |
| 69 | O | 50.27922600 | 35.46602300 | 81.31112900 | 155 | H | 47.33152600 | 37.29914200 | 81.96400000 |
| 70 | H | 54.29618400 | 42.97934700 | 92.66444700 | 156 | H | 55.16162800 | 38.88867600 | 90.16893800 |
| 71 | H | 53.39767200 | 44.12026000 | 91.70019100 | 157 | H | 54.82875400 | 37.73382100 | 91.17698100 |
| 72 | H | 53.61295000 | 40.15682600 | 92.09771500 | 158 | H | 50.14427900 | 36.39209900 | 80.99480900 |
| 73 | H | 52.65545400 | 39.05249700 | 90.06019200 | 159 | H | 51.17336400 | 35.25964300 | 80.99800600 |
| 74 | H | 52.34686100 | 44.42242000 | 89.49088300 | 160 | H | 46.33125900 | 33.86847200 | 82.80755300 |
| 75 | H | 51.38125200 | 44.16169900 | 87.22131700 | 161 | H | 46.67994622 | 42.77849940 | 80.60770839 |
| 76 | H | 51.73661800 | 39.87711200 | 87.47800900 | 162 | H | 57.74688906 | 36.14080158 | 88.50492543 |
| 77 | H | 51.08306400 | 41.90843000 | 86.23192300 | 163 | H | 52.58782611 | 43.19830161 | 92.93990956 |
| 78 | H | 48.50030900 | 35.65610400 | 78.53921500 | 164 | H | 53.89941155 | 34.52568138 | 89.46763760 |
| 79 | H | 47.97753100 | 37.04532000 | 77.61425700 | 165 | H | 45.95748777 | 34.78991754 | 87.71230278 |
| 80 | H | 48.90016300 | 35.99684500 | 75.52371300 | 166 | H | 44.05948599 | 34.12221862 | 82.85754101 |
| 81 | H | 50.40862700 | 33.99094100 | 75.73977800 | 167 | H | 53.53461292 | 32.08995327 | 85.97890950 |
| 82 | H | 48.72709200 | 33.58282200 | 76.04106800 | 168 | H | 47.19594483 | 35.49104837 | 77.38198395 |
| 83 | H | 49.84939500 | 33.77048200 | 77.40061300 | 169 | H | 47.73457954 | 38.85062884 | 85.60790657 |
| 84 | H | 51.27766100 | 36.33753900 | 76.10440000 | 170 | H | 51.96228793 | 31.07040208 | 78.43291921 |
| 85 | H | 50.27156200 | 37.55623300 | 76.89896300 | 171 | H | 55.63826274 | 35.94844740 | 76.18578451 |
| 86 | H | 50.90364300 | 36.19200800 | 77.82673800 | 172 | H | 53.21248668 | 38.11747675 | 85.21068420 |

| **TS** | | | | | | | | | |
| --- | --- | --- | --- | --- | --- | --- | --- | --- | --- |
| 1 | C | 53.31479400 | 43.15415100 | 92.17684900 | 87 | H | 43.95424700 | 35.44743800 | 84.01913300 |
| 2 | C | 53.07175400 | 42.11316500 | 91.12534000 | 88 | H | 43.44876600 | 35.75966500 | 82.37659100 |
| 3 | C | 53.20656500 | 40.74359700 | 91.26165300 | 89 | H | 44.90326500 | 33.49511000 | 88.18491600 |
| 4 | C | 52.51729100 | 42.32399400 | 89.81800000 | 90 | H | 44.86772200 | 32.67569600 | 85.83097400 |
| 5 | N | 52.78397100 | 40.09568700 | 90.12971700 | 91 | H | 45.79518500 | 34.08471500 | 85.31132500 |
| 6 | C | 52.33455100 | 41.04063000 | 89.22955700 | 92 | H | 44.13774700 | 34.26773900 | 85.93619000 |
| 7 | C | 52.18815200 | 43.47072400 | 89.07603500 | 93 | H | 46.58148600 | 31.83939500 | 87.32215000 |
| 8 | C | 51.82900400 | 40.89069100 | 87.93748800 | 94 | H | 47.34755200 | 32.84736700 | 88.53944800 |
| 9 | C | 51.67180100 | 43.32456800 | 87.79494000 | 95 | H | 47.65617400 | 33.15499800 | 86.83383200 |
| 10 | C | 51.49258200 | 42.04615800 | 87.23470800 | 96 | H | 48.46126200 | 37.91557800 | 84.40219800 |
| 11 | C | 48.12846600 | 35.97337900 | 77.53095700 | 97 | H | 47.17436100 | 37.21209000 | 85.34601900 |
| 12 | C | 49.23375200 | 35.67032000 | 76.49848000 | 98 | H | 49.65377600 | 38.85826500 | 87.85961400 |
| 13 | C | 50.49058900 | 36.48145100 | 76.84935000 | 99 | H | 51.20824800 | 37.02747300 | 88.72336700 |
| 14 | C | 49.55558800 | 34.17268900 | 76.40896700 | 100 | H | 50.86569600 | 34.86263300 | 87.34835800 |
| 15 | C | 44.15893700 | 35.19098300 | 82.97279700 | 101 | H | 53.09769700 | 30.09196200 | 79.27544700 |
| 16 | C | 45.52613700 | 35.71018900 | 82.67984100 | 102 | H | 51.60823200 | 30.60902200 | 80.05415200 |
| 17 | O | 46.57355800 | 34.86968300 | 82.68495700 | 103 | H | 53.91837400 | 32.47258800 | 79.32339500 |
| 18 | O | 45.72569400 | 36.89749500 | 82.47273700 | 104 | H | 52.61108700 | 34.10259500 | 80.71596600 |
| 19 | C | 45.66554100 | 33.81074400 | 87.46385200 | 105 | H | 51.80018500 | 33.72179400 | 79.19411000 |
| 20 | C | 45.07779400 | 33.71945700 | 86.05717500 | 106 | H | 51.23091500 | 32.96758700 | 80.69540300 |
| 21 | C | 46.87984600 | 32.86973800 | 87.55366000 | 107 | H | 54.28639900 | 32.57300300 | 81.75824600 |
| 22 | C | 48.03402900 | 37.88287300 | 85.41375200 | 108 | H | 52.93272800 | 31.46645300 | 82.03788000 |
| 23 | C | 49.04456000 | 37.30694200 | 86.35999400 | 109 | H | 54.38635800 | 30.89223400 | 81.22363600 |
| 24 | N | 49.45014800 | 35.99439700 | 86.22323200 | 110 | H | 56.98148100 | 35.66749600 | 77.28749000 |
| 25 | C | 49.72451800 | 37.87961000 | 87.41209400 | 111 | H | 55.96086200 | 34.33406000 | 76.84432900 |
| 26 | C | 50.34404600 | 35.79416600 | 87.18057700 | 112 | H | 56.53904200 | 34.43291100 | 79.49768100 |
| 27 | N | 50.54310700 | 36.90313500 | 87.93003600 | 113 | H | 53.56936000 | 36.86614600 | 77.56209500 |
| 28 | C | 46.40607600 | 41.76632700 | 80.25564400 | 114 | H | 55.48502800 | 34.91432300 | 81.68387500 |
| 29 | C | 47.43278200 | 40.66711200 | 80.24861900 | 115 | H | 52.58266600 | 37.39938200 | 79.73473300 |
| 30 | C | 48.63615800 | 40.80668300 | 79.48408800 | 116 | H | 53.58401000 | 36.49943300 | 81.83041700 |
| 31 | C | 47.21877000 | 39.48876200 | 80.95413500 | 117 | H | 53.47777000 | 38.52068100 | 83.56374300 |
| 32 | C | 49.58857000 | 39.81935600 | 79.44804900 | 118 | H | 51.41229400 | 37.24399600 | 82.91373900 |
| 33 | C | 48.19303800 | 38.48280600 | 80.97040300 | 119 | H | 52.55093600 | 36.18721900 | 83.71225100 |
| 34 | C | 49.41053900 | 38.63084200 | 80.20291300 | 120 | H | 51.15573700 | 36.79719700 | 84.59567100 |
| 35 | O | 48.11631600 | 37.37057000 | 81.67833300 | 121 | H | 52.24512500 | 40.38823200 | 84.86012800 |
| 36 | O | 50.32226900 | 37.70941800 | 80.21776500 | 122 | H | 50.88700900 | 39.29171800 | 84.97472300 |
| 37 | C | 52.39357500 | 30.92896000 | 79.35981900 | 123 | H | 50.63768700 | 40.84137500 | 83.01256800 |
| 38 | C | 53.10075300 | 32.13968100 | 79.97543100 | 124 | H | 52.16973900 | 40.28043000 | 82.35533300 |
| 39 | C | 52.12275900 | 33.30287700 | 80.15322900 | 125 | H | 50.79269000 | 39.18879500 | 82.43801900 |
| 40 | C | 53.71416100 | 31.74882900 | 81.32283200 | 126 | H | 57.05801100 | 37.58294700 | 89.20476300 |
| 41 | C | 55.93658500 | 35.40011000 | 77.08450000 | 127 | H | 58.64630500 | 37.64045800 | 88.42768700 |
| 42 | C | 55.16865800 | 35.64780300 | 78.36446900 | 128 | H | 59.51644000 | 36.08800800 | 90.27854600 |
| 43 | C | 54.02470400 | 36.45022600 | 78.45607600 | 129 | H | 57.95170000 | 36.21147800 | 91.06460600 |
| 44 | C | 55.67439100 | 35.08998600 | 79.55042900 | 130 | H | 59.90035200 | 38.50709300 | 90.51729800 |
| 45 | C | 53.45234100 | 36.75379900 | 79.69416600 | 131 | H | 59.67956600 | 37.66648100 | 92.06062600 |
| 46 | C | 55.09688200 | 35.37580200 | 80.78378800 | 132 | H | 57.08605000 | 38.64751500 | 91.32219800 |
| 47 | C | 54.00431300 | 36.24101000 | 80.86591100 | 133 | H | 56.34071800 | 40.55130400 | 92.26675000 |
| 48 | C | 52.70296400 | 38.31031500 | 84.30990800 | 134 | H | 57.41149900 | 41.75127700 | 92.78348800 |
| 49 | C | 51.76536700 | 39.53831300 | 84.36460300 | 135 | H | 60.30630200 | 39.99284100 | 92.32166300 |
| 50 | C | 51.92011600 | 37.06815900 | 83.86484700 | 136 | H | 59.65605000 | 41.50968000 | 92.58039600 |
| 51 | C | 51.31453200 | 39.98543300 | 82.97005900 | 137 | H | 52.25415600 | 34.48880000 | 90.07425100 |
| 52 | C | 57.98256900 | 37.02814300 | 89.04846400 | 138 | H | 52.61879700 | 35.31050300 | 88.55810600 |
| 53 | C | 58.64709800 | 36.74138300 | 90.40604400 | 139 | H | 53.43746300 | 31.53663100 | 84.34773500 |
| 54 | C | 59.16761100 | 37.98109400 | 91.13965800 | 140 | H | 54.43973200 | 32.92333600 | 84.72070400 |
| 55 | N | 58.08867800 | 38.91365700 | 91.48870900 | 141 | H | 52.44100900 | 33.58622700 | 83.50805100 |
| 56 | C | 58.30394400 | 40.13554700 | 91.93814600 | 142 | H | 52.37444700 | 34.31682500 | 85.09068700 |
| 57 | N | 59.56304500 | 40.63487400 | 92.05333400 | 143 | H | 50.53112400 | 33.01066800 | 85.77912700 |
| 58 | N | 57.26058100 | 40.97200700 | 92.14324900 | 144 | H | 51.02247200 | 31.64792400 | 84.80549500 |
| 59 | C | 52.99615400 | 35.10918400 | 89.56009700 | 145 | H | 48.95690000 | 32.49679800 | 83.97689600 |
| 60 | C | 53.11493300 | 36.42017000 | 90.33441300 | 146 | H | 50.21744000 | 32.67465300 | 82.75838900 |
| 61 | O | 53.91479200 | 36.47892200 | 91.31155600 | 147 | H | 48.57877400 | 34.57665800 | 83.32024800 |
| 62 | O | 52.32269200 | 37.35252900 | 89.99029300 | 148 | H | 49.61092100 | 34.96699100 | 84.49129900 |
| 63 | C | 53.48840800 | 32.44076000 | 84.96729200 | 149 | H | 50.07239000 | 35.22359100 | 82.22876800 |
| 64 | C | 52.31703700 | 33.35585900 | 84.57331200 | 150 | H | 46.05868400 | 41.91952400 | 79.22534500 |
| 65 | C | 50.92221700 | 32.73606700 | 84.79183400 | 151 | H | 45.53423700 | 41.46527700 | 80.84208600 |
| 66 | C | 49.86885900 | 33.05543000 | 83.72602200 | 152 | H | 48.79340000 | 41.71964000 | 78.91758700 |
| 67 | N | 49.56335200 | 34.50780800 | 83.56917100 | 153 | H | 46.31249500 | 39.34610400 | 81.52959000 |
| 68 | O | 55.48914300 | 38.50690900 | 90.97508300 | 154 | H | 50.49055000 | 39.91797000 | 78.85791100 |
| 69 | O | 50.22635500 | 35.51474400 | 81.23375200 | 155 | H | 47.20713800 | 37.24361700 | 82.07635000 |
| 70 | H | 54.28356500 | 42.99527900 | 92.67014700 | 156 | H | 55.21755300 | 38.83727700 | 90.10211800 |
| 71 | H | 53.38270600 | 44.14471500 | 91.71546500 | 157 | H | 54.84257700 | 37.75463400 | 91.17743700 |
| 72 | H | 53.59005600 | 40.17566700 | 92.09752400 | 158 | H | 50.16349300 | 36.76110800 | 80.77901700 |
| 73 | H | 52.66381600 | 39.07021500 | 90.05728200 | 159 | H | 51.10777600 | 35.18277100 | 80.99652200 |
| 74 | H | 52.36383400 | 44.45713100 | 89.48998300 | 160 | H | 46.29867300 | 33.94483200 | 82.80707200 |
| 75 | H | 51.41140400 | 44.20130200 | 87.21301300 | 161 | H | 46.73051501 | 42.72329397 | 80.60757234 |
| 76 | H | 51.71166000 | 39.91226000 | 87.48609900 | 162 | H | 47.18508108 | 35.49695749 | 77.36378214 |
| 77 | H | 51.09909400 | 41.94809100 | 86.22819600 | 163 | H | 45.94007597 | 34.81689166 | 87.70301187 |
| 78 | H | 48.48607400 | 35.66851400 | 78.52123200 | 164 | H | 53.90074247 | 34.54269944 | 89.48449973 |
| 79 | H | 47.96544300 | 37.05376500 | 77.58724200 | 165 | H | 53.54957312 | 32.11121949 | 85.98344252 |
| 80 | H | 48.90067600 | 36.00218900 | 75.50884800 | 166 | H | 52.57514891 | 43.21106594 | 92.94794292 |
| 81 | H | 50.39929700 | 33.98788300 | 75.73500400 | 167 | H | 47.68649568 | 38.86913558 | 85.64048250 |
| 82 | H | 48.71354800 | 33.58863300 | 76.02161100 | 168 | H | 53.20446421 | 38.12715879 | 85.23718969 |
| 83 | H | 49.82426500 | 33.76883300 | 77.39089000 | 169 | H | 51.93950464 | 31.07773218 | 78.40243353 |
| 84 | H | 51.27208300 | 36.34197600 | 76.09619600 | 170 | H | 55.64096157 | 35.93153189 | 76.20410374 |
| 85 | H | 50.26893400 | 37.55197400 | 76.90575700 | 171 | H | 57.75169519 | 36.13544369 | 88.50561483 |
| 86 | H | 50.90287900 | 36.17288900 | 77.81543400 | 172 | H | 43.99957845 | 34.13907702 | 82.85878535 |

| **PC** | | | | | | | | | |
| --- | --- | --- | --- | --- | --- | --- | --- | --- | --- |
| 1 | C | 53.31735400 | 43.15397500 | 92.16455000 | 87 | H | 43.97903400 | 35.45117400 | 84.00920200 |
| 2 | C | 53.06890100 | 42.11144200 | 91.11675000 | 88 | H | 43.45360500 | 35.75392800 | 82.37148500 |
| 3 | C | 53.20920700 | 40.73977600 | 91.25595500 | 89 | H | 44.89827500 | 33.50489500 | 88.18393900 |
| 4 | C | 52.50690600 | 42.31641300 | 89.81277300 | 90 | H | 44.85207500 | 32.68880600 | 85.83321900 |
| 5 | N | 52.78108000 | 40.08918000 | 90.13160300 | 91 | H | 45.79989100 | 34.08390900 | 85.31066400 |
| 6 | C | 52.32545000 | 41.03078700 | 89.22915800 | 92 | H | 44.14361700 | 34.29031800 | 85.93107700 |
| 7 | C | 52.17044100 | 43.45996400 | 89.06895600 | 93 | H | 46.57264100 | 31.84533100 | 87.32146800 |
| 8 | C | 51.81712100 | 40.87458200 | 87.94018500 | 94 | H | 47.34020500 | 32.84947900 | 88.54073100 |
| 9 | C | 51.64842300 | 43.30781800 | 87.79016400 | 95 | H | 47.65182300 | 33.15844200 | 86.83610700 |
| 10 | C | 51.47260700 | 42.02775300 | 87.23466500 | 96 | H | 48.46470500 | 37.90762300 | 84.40697200 |
| 11 | C | 48.11652900 | 35.97993300 | 77.53976400 | 97 | H | 47.17261700 | 37.20915800 | 85.34667900 |
| 12 | C | 49.22516000 | 35.67655400 | 76.51092500 | 98 | H | 49.65674500 | 38.86419100 | 87.85534300 |
| 13 | C | 50.47721500 | 36.49710900 | 76.85673400 | 99 | H | 51.20268800 | 37.03024000 | 88.73178400 |
| 14 | C | 49.55521200 | 34.17996700 | 76.43230200 | 100 | H | 50.84091100 | 34.85530300 | 87.38192800 |
| 15 | C | 44.17403000 | 35.19168600 | 82.96195500 | 101 | H | 53.10770800 | 30.09773800 | 79.29989600 |
| 16 | C | 45.53367000 | 35.71973000 | 82.64889600 | 102 | H | 51.61788900 | 30.61142000 | 80.08065900 |
| 17 | O | 46.58434600 | 34.88530500 | 82.61540700 | 103 | H | 53.91904500 | 32.48510800 | 79.34692800 |
| 18 | O | 45.72367500 | 36.91057200 | 82.45331700 | 104 | H | 52.61343400 | 34.10241100 | 80.75410200 |
| 19 | C | 45.66213200 | 33.81914900 | 87.46395300 | 105 | H | 51.78999800 | 33.72066800 | 79.23916300 |
| 20 | C | 45.07630000 | 33.73045000 | 86.05628100 | 106 | H | 51.24047200 | 32.96265700 | 80.74548100 |
| 21 | C | 46.87370500 | 32.87456800 | 87.55437900 | 107 | H | 54.30189600 | 32.57751100 | 81.77962200 |
| 22 | C | 48.03312200 | 37.87843000 | 85.41699400 | 108 | H | 52.95839400 | 31.46006100 | 82.06272900 |
| 23 | C | 49.03776300 | 37.30307700 | 86.37019300 | 109 | H | 54.41081100 | 30.90025800 | 81.23559300 |
| 24 | N | 49.43208300 | 35.98603900 | 86.24791300 | 110 | H | 56.98285700 | 35.69666700 | 77.28999200 |
| 25 | C | 49.72250800 | 37.88154100 | 87.41612600 | 111 | H | 55.97402700 | 34.35331500 | 76.84885200 |
| 26 | C | 50.32531800 | 35.78866600 | 87.20580000 | 112 | H | 56.54866200 | 34.45063000 | 79.49936400 |
| 27 | N | 50.53401400 | 36.90394100 | 87.94376800 | 113 | H | 53.56381300 | 36.86965100 | 77.56943800 |
| 28 | C | 46.37214500 | 41.79739900 | 80.24149900 | 114 | H | 55.49350600 | 34.92322600 | 81.68691200 |
| 29 | C | 47.40148200 | 40.70126900 | 80.21949100 | 115 | H | 52.58025100 | 37.40032200 | 79.74344300 |
| 30 | C | 48.61015700 | 40.86384200 | 79.47054400 | 116 | H | 53.58709400 | 36.50035800 | 81.83805500 |
| 31 | C | 47.18644800 | 39.50709100 | 80.89720900 | 117 | H | 53.47617800 | 38.49249700 | 83.56518000 |
| 32 | C | 49.57294500 | 39.88453400 | 79.42778700 | 118 | H | 51.39751800 | 37.20861900 | 82.93922900 |
| 33 | C | 48.16620700 | 38.50542200 | 80.89857200 | 119 | H | 52.55400900 | 36.16196700 | 83.72338600 |
| 34 | C | 49.39120100 | 38.68586400 | 80.15524200 | 120 | H | 51.16786500 | 36.76542800 | 84.62485800 |
| 35 | O | 48.08642600 | 37.37015200 | 81.56881700 | 121 | H | 52.23076500 | 40.36336500 | 84.86538100 |
| 36 | O | 50.32000000 | 37.77000000 | 80.17000300 | 122 | H | 50.87445100 | 39.26125900 | 84.95259200 |
| 37 | C | 52.40220300 | 30.93349400 | 79.38616800 | 123 | H | 50.63207200 | 40.79681500 | 82.98870400 |
| 38 | C | 53.10745600 | 32.14490200 | 80.00283000 | 124 | H | 52.19937200 | 40.29166000 | 82.37120800 |
| 39 | C | 52.12484500 | 33.30155000 | 80.19404500 | 125 | H | 50.85663900 | 39.15662900 | 82.40035400 |
| 40 | C | 53.73287500 | 31.75123900 | 81.34386800 | 126 | H | 57.07082800 | 37.58225300 | 89.20567500 |
| 41 | C | 55.94034900 | 35.41929100 | 77.08808600 | 127 | H | 58.66090800 | 37.63446800 | 88.43244400 |
| 42 | C | 55.17120400 | 35.66006700 | 78.36890900 | 128 | H | 59.52129100 | 36.08089800 | 90.28802200 |
| 43 | C | 54.02286300 | 36.45589300 | 78.46261400 | 129 | H | 57.95590800 | 36.21380800 | 91.07109600 |
| 44 | C | 55.68085800 | 35.10338600 | 79.55374800 | 130 | H | 59.91482000 | 38.49960300 | 90.51957500 |
| 45 | C | 53.45111700 | 36.75643200 | 79.70185500 | 131 | H | 59.69326400 | 37.66174400 | 92.06441500 |
| 46 | C | 55.10305100 | 35.38474900 | 80.78793300 | 132 | H | 57.10336000 | 38.65429400 | 91.32699500 |
| 47 | C | 54.00696500 | 36.24527600 | 80.87242300 | 133 | H | 56.37205000 | 40.56219400 | 92.28551400 |
| 48 | C | 52.70321100 | 38.28700800 | 84.31476400 | 134 | H | 57.45248000 | 41.75885400 | 92.78863500 |
| 49 | C | 51.76245400 | 39.51318300 | 84.35887900 | 135 | H | 60.33394900 | 39.98404500 | 92.31877400 |
| 50 | C | 51.92059400 | 37.03963700 | 83.88325500 | 136 | H | 59.69016500 | 41.50389400 | 92.57976700 |
| 51 | C | 51.33654500 | 39.96287900 | 82.95709100 | 137 | H | 52.26479900 | 34.49427500 | 90.08503300 |
| 52 | C | 57.99440800 | 37.02487200 | 89.05289000 | 138 | H | 52.62766500 | 35.31056000 | 88.56549800 |
| 53 | C | 58.65496200 | 36.73874900 | 90.41248500 | 139 | H | 53.44081400 | 31.52076900 | 84.36719700 |
| 54 | C | 59.18119200 | 37.97720000 | 91.14386400 | 140 | H | 54.44253700 | 32.91039900 | 84.73140100 |
| 55 | N | 58.10699600 | 38.91526600 | 91.49423500 | 141 | H | 52.43274900 | 33.56384400 | 83.52610900 |
| 56 | C | 58.33050900 | 40.13596100 | 91.94267900 | 142 | H | 52.37979200 | 34.30280600 | 85.10478100 |
| 57 | N | 59.59221000 | 40.62897800 | 92.05370700 | 143 | H | 50.53889000 | 33.00895200 | 85.81242500 |
| 58 | N | 57.29239700 | 40.97848800 | 92.15214200 | 144 | H | 51.02487400 | 31.63332900 | 84.85531500 |
| 59 | C | 53.00674400 | 35.11211100 | 89.56754400 | 145 | H | 48.95530900 | 32.47311100 | 84.02538300 |
| 60 | C | 53.12919400 | 36.42539800 | 90.33714500 | 146 | H | 50.20927700 | 32.63769900 | 82.79784800 |
| 61 | O | 53.93084400 | 36.48697300 | 91.31276200 | 147 | H | 48.58757400 | 34.55141500 | 83.32722200 |
| 62 | O | 52.33814300 | 37.35863400 | 89.99127900 | 148 | H | 49.59653800 | 34.93707100 | 84.51814000 |
| 63 | C | 53.49295400 | 32.42696000 | 84.98369200 | 149 | H | 50.09352700 | 35.25000400 | 82.20067600 |
| 64 | C | 52.31809700 | 33.33894600 | 84.59337400 | 150 | H | 46.01773800 | 41.95945500 | 79.21499000 |
| 65 | C | 50.92469100 | 32.72114600 | 84.82666100 | 151 | H | 45.50419400 | 41.49118200 | 80.83096700 |
| 66 | C | 49.86593100 | 33.02990700 | 83.76294100 | 152 | H | 48.76714100 | 41.78771500 | 78.92204900 |
| 67 | N | 49.56654900 | 34.48037300 | 83.59541300 | 153 | H | 46.27468200 | 39.34584100 | 81.45936800 |
| 68 | O | 55.50378800 | 38.51494400 | 90.97151200 | 154 | H | 50.48291200 | 40.00365400 | 78.85389400 |
| 69 | O | 50.23566400 | 35.54197300 | 81.22578200 | 155 | H | 47.18699800 | 37.24473500 | 81.98931600 |
| 70 | H | 54.28828900 | 42.99454100 | 92.65319100 | 156 | H | 55.24520100 | 38.82627100 | 90.08760700 |
| 71 | H | 53.38291000 | 44.14364200 | 91.70114300 | 157 | H | 54.85907800 | 37.76110400 | 91.17565200 |
| 72 | H | 53.60249900 | 40.17639100 | 92.09033800 | 158 | H | 50.14925100 | 36.87115000 | 80.71658800 |
| 73 | H | 52.67103700 | 39.06022100 | 90.05954000 | 159 | H | 51.12715300 | 35.23285600 | 80.99714600 |
| 74 | H | 52.34585400 | 44.44814900 | 89.47843900 | 160 | H | 46.31549200 | 33.95883300 | 82.73914800 |
| 75 | H | 51.38240800 | 44.18177500 | 87.20677300 | 161 | H | 52.58157841 | 43.21225838 | 92.93923531 |
| 76 | H | 51.70327100 | 39.89484300 | 87.49113000 | 162 | H | 53.91066548 | 34.54441323 | 89.49307786 |
| 77 | H | 51.07674000 | 41.92471900 | 86.22951800 | 163 | H | 57.76286406 | 36.13168114 | 88.51113591 |
| 78 | H | 48.47058600 | 35.67744700 | 78.53187800 | 164 | H | 53.55873568 | 32.10041144 | 86.00051982 |
| 79 | H | 47.95186200 | 37.06008700 | 77.59365200 | 165 | H | 55.63889630 | 35.94801807 | 76.20804398 |
| 80 | H | 48.89147500 | 35.99942800 | 75.51845100 | 166 | H | 51.94744203 | 31.08286011 | 78.42920289 |
| 81 | H | 50.40255000 | 33.99529000 | 75.76290700 | 167 | H | 47.17455139 | 35.50196923 | 77.36909231 |
| 82 | H | 48.71744700 | 33.58943900 | 76.04556800 | 168 | H | 47.68660195 | 38.86587502 | 85.64009978 |
| 83 | H | 49.82201300 | 33.78410500 | 77.41798900 | 169 | H | 53.20748823 | 38.10991305 | 85.24171700 |
| 84 | H | 51.26009300 | 36.35595400 | 76.10529800 | 170 | H | 46.70090540 | 42.75055061 | 80.59971487 |
| 85 | H | 50.25016500 | 37.56711000 | 76.90436500 | 171 | H | 44.01905635 | 34.13859915 | 82.85290797 |
| 86 | H | 50.89006500 | 36.19800400 | 77.82540200 | 172 | H | 45.93815223 | 34.82472980 | 87.70378645 |

**Cartesian coordinates for the DOPA radical regeneration using O_2_^•-^ as the oxidant in the protonated state of Asp88 (Pathway C).**

| **RC** | | | | | | | | | |
| --- | --- | --- | --- | --- | --- | --- | --- | --- | --- |
| 1 | C | 50.97046100 | 67.25047400 | 56.17976900 | 63 | C | 52.06105100 | 63.84033300 | 58.21241200 |
| 2 | H | 51.41811900 | 66.96438300 | 57.13984000 | 64 | H | 52.62846500 | 64.02148300 | 59.12764800 |
| 3 | H | 50.72511200 | 68.31403100 | 56.27134500 | 65 | H | 52.12208600 | 64.74464600 | 57.60178300 |
| 4 | C | 52.01333100 | 67.05730700 | 55.04940200 | 66 | H | 51.01817000 | 63.68578500 | 58.49910000 |
| 5 | H | 51.74963900 | 67.72988600 | 54.22704600 | 67 | C | 58.92215600 | 66.38711400 | 55.77363300 |
| 6 | C | 53.39464800 | 67.48628400 | 55.54928300 | 68 | H | 59.72541500 | 67.02262400 | 56.17264600 |
| 7 | H | 53.37599000 | 68.48674300 | 55.99202400 | 69 | H | 59.38485900 | 65.41730400 | 55.57206300 |
| 8 | H | 53.76650400 | 66.79637700 | 56.31099000 | 70 | C | 57.84146000 | 66.23402600 | 56.81745600 |
| 9 | H | 54.12608000 | 67.49520400 | 54.73208700 | 71 | C | 56.84099600 | 67.19766600 | 57.00625000 |
| 10 | C | 52.08352000 | 65.63293800 | 54.46818900 | 72 | H | 56.80598600 | 68.08187200 | 56.37459200 |
| 11 | H | 52.30688200 | 64.89140400 | 55.24267600 | 73 | C | 55.86006000 | 67.03652600 | 57.98250300 |
| 12 | H | 51.14955200 | 65.35028400 | 53.97558500 | 74 | H | 55.08004800 | 67.78033800 | 58.09585700 |
| 13 | H | 52.87666300 | 65.57294400 | 53.71572900 | 75 | C | 55.85294500 | 65.89485200 | 58.78410700 |
| 14 | C | 48.87205500 | 65.50882100 | 61.90478700 | 76 | H | 55.04542800 | 65.74888500 | 59.49084200 |
| 15 | H | 48.51724900 | 66.05336900 | 62.78098900 | 77 | C | 56.85392300 | 64.93881100 | 58.62583000 |
| 16 | H | 48.85951800 | 66.16882700 | 61.03283000 | 78 | H | 56.86087900 | 64.04172600 | 59.23903000 |
| 17 | C | 50.29785800 | 65.02345200 | 62.15837800 | 79 | C | 57.83849500 | 65.11211000 | 57.65435400 |
| 18 | O | 50.97536600 | 64.62782600 | 61.15215300 | 80 | H | 58.60934600 | 64.35678600 | 57.53787200 |
| 19 | O | 50.76889700 | 64.94763700 | 63.33351900 | 81 | C | 56.59040500 | 68.72340100 | 62.75570700 |
| 20 | C | 48.91124300 | 64.42841000 | 65.97904300 | 82 | H | 57.37495700 | 69.17927900 | 62.14545400 |
| 21 | H | 48.55959300 | 64.46876700 | 64.94418100 | 83 | C | 56.11201300 | 67.47415600 | 62.01051800 |
| 22 | C | 49.98649700 | 63.33511300 | 66.06071100 | 84 | H | 56.93927600 | 66.81688600 | 61.71934600 |
| 23 | H | 50.66956100 | 63.32892500 | 65.22016000 | 85 | H | 55.39791000 | 66.90515500 | 62.60776100 |
| 24 | H | 50.56078300 | 63.38450500 | 66.99489600 | 86 | H | 55.61189400 | 67.75747400 | 61.08262900 |
| 25 | H | 49.49385900 | 62.35603100 | 66.07085400 | 87 | C | 55.46572100 | 69.77489000 | 62.92953500 |
| 26 | C | 47.79700100 | 63.98221300 | 66.90258000 | 88 | H | 55.81683900 | 70.59490500 | 63.56745100 |
| 27 | H | 47.39891600 | 63.02677400 | 66.55792300 | 89 | H | 54.62530500 | 69.31997500 | 63.46066300 |
| 28 | H | 48.19429000 | 63.78507600 | 67.89357500 | 90 | C | 54.96958800 | 70.38904400 | 61.62092000 |
| 29 | H | 46.99403700 | 64.70373600 | 67.00752200 | 91 | H | 54.22760500 | 71.17135900 | 61.81389300 |
| 30 | C | 51.76635800 | 68.53213300 | 63.95502300 | 92 | H | 55.79971600 | 70.84650600 | 61.07529500 |
| 31 | H | 52.17287900 | 68.45773500 | 62.94429600 | 93 | H | 54.50839800 | 69.66033600 | 60.95411800 |
| 32 | H | 50.95287000 | 67.80109300 | 64.00499900 | 94 | C | 57.52810200 | 62.84713200 | 63.79367100 |
| 33 | C | 52.84538800 | 68.14422200 | 64.92513800 | 95 | H | 57.50850900 | 61.93640400 | 63.18883700 |
| 34 | N | 53.72043600 | 67.13152900 | 64.57300900 | 96 | H | 58.52900900 | 63.25901900 | 63.66084400 |
| 35 | C | 54.53274500 | 66.95983100 | 65.59869300 | 97 | C | 56.45309000 | 63.81489300 | 63.26285600 |
| 36 | H | 55.34103200 | 66.24489800 | 65.63703100 | 98 | H | 56.89282200 | 64.45336100 | 62.49053800 |
| 37 | N | 54.23110600 | 67.80690300 | 66.61124900 | 99 | H | 56.11903600 | 64.50568700 | 64.04757200 |
| 38 | C | 53.15971900 | 68.57471900 | 66.19397100 | 100 | C | 55.24552300 | 63.09045500 | 62.64895600 |
| 39 | H | 52.74334900 | 69.33264000 | 66.84029800 | 101 | H | 54.77549600 | 62.43547300 | 63.39505800 |
| 40 | C | 49.46455700 | 72.92304100 | 58.19925500 | 102 | H | 55.59896900 | 62.42915100 | 61.85520900 |
| 41 | H | 49.27931800 | 73.18910000 | 57.15095700 | 103 | C | 54.17858500 | 63.99720800 | 62.04054100 |
| 42 | H | 48.50115500 | 72.62924000 | 58.62419800 | 104 | H | 53.46389100 | 63.43199200 | 61.44068000 |
| 43 | C | 50.45561500 | 71.79327300 | 58.32155000 | 105 | H | 54.61647200 | 64.76505900 | 61.40406300 |
| 44 | C | 51.75696800 | 71.86719400 | 57.81394200 | 106 | N | 53.39055200 | 64.69167500 | 63.11873300 |
| 45 | H | 52.06839900 | 72.72557600 | 57.22339100 | 107 | H | 53.63079700 | 65.70004000 | 63.20833300 |
| 46 | C | 52.67740400 | 70.85301000 | 58.07635900 | 108 | H | 52.29622800 | 64.66794100 | 63.08692300 |
| 47 | H | 53.69257400 | 70.90963000 | 57.69722100 | 109 | H | 53.68952600 | 64.29746400 | 64.02379200 |
| 48 | C | 52.32513900 | 69.74935700 | 58.85238700 | 110 | O | 52.78156000 | 66.90104900 | 61.38432300 |
| 49 | O | 53.25899700 | 68.79661300 | 59.12363700 | 111 | H | 54.71991600 | 67.86386600 | 67.50498900 |
| 50 | H | 52.97218400 | 68.23754500 | 59.86525000 | 112 | O | 52.57147600 | 66.21584200 | 60.25974600 |
| 51 | C | 51.00947100 | 69.64782100 | 59.33241400 | 113 | H | 51.84897300 | 65.48715600 | 60.56260800 |
| 52 | C | 50.10173400 | 70.66302000 | 59.06060100 | 114 | O | 50.69468700 | 68.53416200 | 60.07067100 |
| 53 | H | 49.07904800 | 70.58538600 | 59.41360500 | 115 | H | 49.81156700 | 68.64549500 | 60.44421400 |
| 54 | C | 54.14221300 | 62.87907900 | 57.18259400 | 116 | H | 49.77522541 | 73.81555332 | 58.70105864 |
| 55 | H | 54.29773800 | 63.95151200 | 57.02560800 | 117 | H | 58.63261575 | 66.80815272 | 54.83353043 |
| 56 | H | 54.76740900 | 62.60506300 | 58.04087800 | 118 | H | 50.05449146 | 66.70344425 | 56.09817080 |
| 57 | C | 52.64600400 | 62.62761300 | 57.48127700 | 119 | H | 57.45444993 | 62.54867876 | 64.81856174 |
| 58 | H | 52.11701400 | 62.52308100 | 56.52527200 | 120 | H | 49.23629143 | 65.42030516 | 66.21438849 |
| 59 | C | 52.38459300 | 61.36595100 | 58.31189300 | 121 | H | 48.20696740 | 64.69107904 | 61.72078545 |
| 60 | H | 51.31054900 | 61.21626100 | 58.46534400 | 122 | H | 51.36840343 | 69.51902926 | 64.06712982 |
| 61 | H | 52.77085800 | 60.45194000 | 57.85758100 | 123 | H | 54.52089495 | 62.38599712 | 56.31174944 |
| 62 | H | 52.84300100 | 61.45399000 | 59.30444100 | 124 | H | 57.02495111 | 68.51930435 | 63.71195709 |

| **TS** | | | | | | | | | |
| --- | --- | --- | --- | --- | --- | --- | --- | --- | --- |
| 1 | C | 50.97409100 | 67.19222400 | 56.06850100 | 63 | C | 51.94389900 | 63.69993100 | 58.06088600 |
| 2 | H | 51.41837000 | 66.89377800 | 57.02414000 | 64 | H | 52.60156600 | 64.13788200 | 58.81419200 |
| 3 | H | 50.74921000 | 68.26063100 | 56.16572900 | 65 | H | 51.75867200 | 64.48377500 | 57.32269900 |
| 4 | C | 52.01784000 | 66.99150600 | 54.93854300 | 66 | H | 50.99536500 | 63.44732400 | 58.53942300 |
| 5 | H | 51.81721800 | 67.72718800 | 54.15331000 | 67 | C | 58.89992600 | 66.40176400 | 55.76668200 |
| 6 | C | 53.41466900 | 67.30431600 | 55.48130100 | 68 | H | 59.70428500 | 67.03889900 | 56.16042700 |
| 7 | H | 53.45638800 | 68.29944100 | 55.93666000 | 69 | H | 59.35653400 | 65.42517500 | 55.58475600 |
| 8 | H | 53.71419900 | 66.58178200 | 56.24534400 | 70 | C | 57.80872200 | 66.27713900 | 56.80258200 |
| 9 | H | 54.16799700 | 67.27369500 | 54.68530400 | 71 | C | 56.82744400 | 67.26403100 | 56.97437600 |
| 10 | C | 52.00231000 | 65.60208800 | 54.27321100 | 72 | H | 56.81491000 | 68.14360500 | 56.33533800 |
| 11 | H | 52.12539600 | 64.79787200 | 55.00673000 | 73 | C | 55.83768700 | 67.13254100 | 57.94507600 |
| 12 | H | 51.07226500 | 65.43560800 | 53.72326900 | 74 | H | 55.07596500 | 67.89674200 | 58.05439300 |
| 13 | H | 52.82260000 | 65.52198300 | 53.55282300 | 75 | C | 55.80318700 | 65.99925700 | 58.75747900 |
| 14 | C | 48.85495200 | 65.48865500 | 61.83963400 | 76 | H | 54.99556400 | 65.88947300 | 59.47020000 |
| 15 | H | 48.52393800 | 66.02774600 | 62.72885900 | 77 | C | 56.78198200 | 65.01851100 | 58.61413000 |
| 16 | H | 48.82708600 | 66.15915500 | 60.97504300 | 78 | H | 56.76248300 | 64.12643900 | 59.23450700 |
| 17 | C | 50.27970400 | 64.98006200 | 62.06586600 | 79 | C | 57.77556900 | 65.16127900 | 57.64660900 |
| 18 | O | 50.94880400 | 64.57913500 | 61.05590000 | 80 | H | 58.52565300 | 64.38483100 | 57.53437600 |
| 19 | O | 50.77188500 | 64.91584500 | 63.23022100 | 81 | C | 56.56310300 | 68.73094700 | 62.77170100 |
| 20 | C | 48.93595000 | 64.37935500 | 65.95666400 | 82 | H | 57.33608200 | 69.19951000 | 62.15641500 |
| 21 | H | 48.51537600 | 64.46727700 | 64.95024400 | 83 | C | 56.09813200 | 67.47881300 | 62.02545000 |
| 22 | C | 50.01532700 | 63.28811500 | 65.90054400 | 84 | H | 56.92721600 | 66.81776500 | 61.75128200 |
| 23 | H | 50.52405500 | 63.27611700 | 64.94406800 | 85 | H | 55.38099800 | 66.91386000 | 62.62131900 |
| 24 | H | 50.74616300 | 63.37191800 | 66.71401500 | 86 | H | 55.61622600 | 67.75696500 | 61.08513400 |
| 25 | H | 49.53925400 | 62.30948700 | 66.02650700 | 87 | C | 55.42558500 | 69.76738400 | 62.95288500 |
| 26 | C | 47.87373300 | 63.88419800 | 66.91784100 | 88 | H | 55.78766700 | 70.61096200 | 63.55204400 |
| 27 | H | 47.42960600 | 62.97178100 | 66.51781700 | 89 | H | 54.61393900 | 69.31798400 | 63.53209000 |
| 28 | H | 48.33164300 | 63.58093200 | 67.85556800 | 90 | C | 54.86438100 | 70.33285900 | 61.64643800 |
| 29 | H | 47.09421700 | 64.60881600 | 67.12951500 | 91 | H | 54.14064600 | 71.12996800 | 61.84979600 |
| 30 | C | 51.80927000 | 68.45679500 | 63.95426000 | 92 | H | 55.66476600 | 70.76166800 | 61.03524100 |
| 31 | H | 52.21627000 | 68.39984700 | 62.93889100 | 93 | H | 54.35750700 | 69.58384900 | 61.03493500 |
| 32 | H | 50.99526200 | 67.72584700 | 63.98890700 | 94 | C | 57.41515900 | 62.88466900 | 63.84778700 |
| 33 | C | 52.88309800 | 68.04897900 | 64.92381200 | 95 | H | 57.33276100 | 61.97667300 | 63.24274400 |
| 34 | N | 53.74601700 | 67.02029600 | 64.58165200 | 96 | H | 58.41831400 | 63.26978900 | 63.65599700 |
| 35 | C | 54.54232000 | 66.83863300 | 65.61643500 | 97 | C | 56.35154400 | 63.89368200 | 63.35918500 |
| 36 | H | 55.33431300 | 66.10763600 | 65.66728800 | 98 | H | 56.77961400 | 64.43562200 | 62.50911600 |
| 37 | N | 54.24825800 | 67.69838700 | 66.62008300 | 99 | H | 56.13834500 | 64.65605800 | 64.11468400 |
| 38 | C | 53.19656400 | 68.48377500 | 66.19035700 | 100 | C | 55.03827100 | 63.24207800 | 62.88836600 |
| 39 | H | 52.78755900 | 69.25203300 | 66.82850300 | 101 | H | 54.42311600 | 62.92610700 | 63.73858300 |
| 40 | C | 49.36234000 | 72.92635800 | 58.20926200 | 102 | H | 55.31350800 | 62.32731000 | 62.35845200 |
| 41 | H | 49.18893800 | 73.18223900 | 57.15654500 | 103 | C | 54.21245100 | 64.04739700 | 61.87527100 |
| 42 | H | 48.39522500 | 72.64609200 | 58.63423600 | 104 | H | 53.56218400 | 63.38989600 | 61.29495500 |
| 43 | C | 50.34281400 | 71.79575600 | 58.34313000 | 105 | H | 54.88122600 | 64.54773700 | 61.17190700 |
| 44 | C | 51.64303300 | 71.85962200 | 57.79521500 | 106 | N | 53.29577700 | 65.10341800 | 62.42098500 |
| 45 | H | 51.92419000 | 72.71562400 | 57.18823200 | 107 | H | 52.99234000 | 65.67792400 | 61.60910000 |
| 46 | C | 52.56640100 | 70.86427200 | 58.04683100 | 108 | H | 52.38293300 | 64.76816800 | 62.85440900 |
| 47 | H | 53.56955100 | 70.90551300 | 57.63664800 | 109 | H | 53.74308900 | 65.75881900 | 63.08862200 |
| 48 | C | 52.24859700 | 69.74079500 | 58.85568700 | 110 | O | 52.73973200 | 66.68041600 | 60.05760600 |
| 49 | O | 53.13574400 | 68.81558900 | 59.08025000 | 111 | H | 54.73733900 | 67.76215000 | 67.51252300 |
| 50 | H | 52.86890700 | 67.76587400 | 59.62878600 | 112 | O | 51.56053000 | 66.33052100 | 59.41800400 |
| 51 | C | 50.93076000 | 69.69734000 | 59.41786500 | 113 | H | 51.22018800 | 65.51788800 | 59.98442000 |
| 52 | C | 50.01170300 | 70.69983500 | 59.13204500 | 114 | O | 50.66329500 | 68.67701800 | 60.26730300 |
| 53 | H | 49.01985000 | 70.65639600 | 59.56496200 | 115 | H | 49.74773500 | 68.74060900 | 60.57212200 |
| 54 | C | 54.09540900 | 62.84675400 | 57.10788300 | 116 | H | 51.41127604 | 69.44207059 | 64.07969050 |
| 55 | H | 54.16669900 | 63.92360000 | 56.92102100 | 117 | H | 49.67888846 | 73.82201753 | 58.70169582 |
| 56 | H | 54.73880800 | 62.64699900 | 57.97312400 | 118 | H | 48.18139722 | 64.67638404 | 61.66232114 |
| 57 | C | 52.62394200 | 62.48532100 | 57.42347800 | 119 | H | 50.04592378 | 66.66408158 | 56.00163391 |
| 58 | H | 52.11156800 | 62.25942900 | 56.47988000 | 120 | H | 57.40125556 | 62.57790682 | 64.87277659 |
| 59 | C | 52.46829100 | 61.27326800 | 58.34755700 | 121 | H | 49.29015685 | 65.35866595 | 66.20240284 |
| 60 | H | 51.40973400 | 61.02718800 | 58.48350400 | 122 | H | 54.51494911 | 62.36253789 | 56.25089942 |
| 61 | H | 52.96314900 | 60.37410800 | 57.97584800 | 123 | H | 58.61669298 | 66.80822758 | 54.81827997 |
| 62 | H | 52.87956000 | 61.48709900 | 59.34175000 | 124 | H | 57.00505195 | 68.52966245 | 63.72515034 |

| **PC** | | | | | | | | | |
| --- | --- | --- | --- | --- | --- | --- | --- | --- | --- |
| 1 | C | 50.97185500 | 67.22607700 | 56.15106200 | 63 | C | 51.82930500 | 63.56015300 | 57.99962200 |
| 2 | H | 51.40926700 | 66.93621100 | 57.11458000 | 64 | H | 52.31775000 | 63.85030200 | 58.92785400 |
| 3 | H | 50.72727000 | 68.29233500 | 56.23679100 | 65 | H | 51.79971300 | 64.44207300 | 57.35649400 |
| 4 | C | 52.03599900 | 67.04374600 | 55.03903700 | 66 | H | 50.80709100 | 63.27635600 | 58.25425800 |
| 5 | H | 51.80159000 | 67.73595300 | 54.22346300 | 67 | C | 58.94705500 | 66.39551400 | 55.76089400 |
| 6 | C | 53.41018600 | 67.44573800 | 55.58342700 | 68 | H | 59.75337800 | 67.03677800 | 56.14491800 |
| 7 | H | 53.40335800 | 68.45924100 | 55.99886000 | 69 | H | 59.41089400 | 65.42669000 | 55.55794300 |
| 8 | H | 53.73188100 | 66.76911700 | 56.37908700 | 70 | C | 57.88439500 | 66.24567200 | 56.82062800 |
| 9 | H | 54.17210400 | 67.41857500 | 54.79548800 | 71 | C | 56.89430900 | 67.21638200 | 57.02848300 |
| 10 | C | 52.10231900 | 65.63151600 | 54.42993500 | 72 | H | 56.84730600 | 68.09654200 | 56.39163800 |
| 11 | H | 52.34346400 | 64.87803100 | 55.18629000 | 73 | C | 55.94875400 | 67.07143600 | 58.04029400 |
| 12 | H | 51.16201400 | 65.35494500 | 53.94687300 | 74 | H | 55.18969300 | 67.83169700 | 58.18489200 |
| 13 | H | 52.88314800 | 65.59321100 | 53.66402800 | 75 | C | 55.96197600 | 65.93819700 | 58.85315400 |
| 14 | C | 48.86622900 | 65.48487300 | 61.82862700 | 76 | H | 55.19688800 | 65.82846900 | 59.60853900 |
| 15 | H | 48.56935500 | 65.97372300 | 62.75892600 | 77 | C | 56.94160800 | 64.96809100 | 58.66153400 |
| 16 | H | 48.75725500 | 66.19054800 | 60.99844100 | 78 | H | 56.95658000 | 64.07556900 | 59.28075700 |
| 17 | C | 50.33055300 | 65.03355000 | 61.94712100 | 79 | C | 57.89715000 | 65.12658100 | 57.65951800 |
| 18 | O | 50.96455300 | 64.75268200 | 60.89723100 | 80 | H | 58.66010200 | 64.36641600 | 57.52554800 |
| 19 | O | 50.85821700 | 64.91081700 | 63.10786300 | 81 | C | 56.55423600 | 68.74699300 | 62.79720500 |
| 20 | C | 48.90511200 | 64.39346000 | 65.94286500 | 82 | H | 57.32768100 | 69.20384100 | 62.17307200 |
| 21 | H | 48.45124400 | 64.49902300 | 64.95245800 | 83 | C | 56.06127400 | 67.49911700 | 62.06430200 |
| 22 | C | 49.98679900 | 63.31372600 | 65.82506700 | 84 | H | 56.88051500 | 66.83149800 | 61.77587200 |
| 23 | H | 50.49485400 | 63.37068700 | 64.87086300 | 85 | H | 55.34950100 | 66.94113200 | 62.67300800 |
| 24 | H | 50.71301200 | 63.35428000 | 66.64642600 | 86 | H | 55.56065900 | 67.77180600 | 61.13384400 |
| 25 | H | 49.51536700 | 62.32714700 | 65.89142800 | 87 | C | 55.43442300 | 69.79879800 | 62.99602000 |
| 26 | C | 47.87485900 | 63.87212200 | 66.92375700 | 88 | H | 55.81273100 | 70.63733000 | 63.59206300 |
| 27 | H | 47.42851300 | 62.96263400 | 66.51947600 | 89 | H | 54.62222100 | 69.35863200 | 63.58160200 |
| 28 | H | 48.35897000 | 63.55540400 | 67.84422000 | 90 | C | 54.86938000 | 70.37423700 | 61.69737400 |
| 29 | H | 47.09519100 | 64.58743500 | 67.16370200 | 91 | H | 54.15431500 | 71.17829500 | 61.90578900 |
| 30 | C | 51.80694700 | 68.46686400 | 63.95168300 | 92 | H | 55.66865400 | 70.79081400 | 61.07638100 |
| 31 | H | 52.23474400 | 68.40628000 | 62.94495500 | 93 | H | 54.35380200 | 69.62702100 | 61.09307900 |
| 32 | H | 50.99935700 | 67.72808000 | 63.97351300 | 94 | C | 57.44392200 | 62.88095300 | 63.83283800 |
| 33 | C | 52.86971700 | 68.07019000 | 64.93836500 | 95 | H | 57.40880800 | 61.97013300 | 63.22740400 |
| 34 | N | 53.72950900 | 67.03673800 | 64.61194900 | 96 | H | 58.43718100 | 63.30004200 | 63.66450700 |
| 35 | C | 54.51812100 | 66.86279100 | 65.65238300 | 97 | C | 56.35373600 | 63.84624600 | 63.31739400 |
| 36 | H | 55.30602900 | 66.12810800 | 65.71209500 | 98 | H | 56.76642100 | 64.38241000 | 62.45548200 |
| 37 | N | 54.22298400 | 67.73363900 | 66.64726900 | 99 | H | 56.11723700 | 64.61797600 | 64.05782000 |
| 38 | C | 53.17628100 | 68.51841600 | 66.20280300 | 100 | C | 55.06777100 | 63.13261000 | 62.86081700 |
| 39 | H | 52.76513900 | 69.29333900 | 66.83156700 | 101 | H | 54.47869200 | 62.79112000 | 63.72027600 |
| 40 | C | 49.28951200 | 72.93785700 | 58.22710700 | 102 | H | 55.38540500 | 62.23455100 | 62.32876700 |
| 41 | H | 49.06288800 | 73.19725100 | 57.18493800 | 103 | C | 54.18910800 | 63.89730200 | 61.86543100 |
| 42 | H | 48.35745800 | 72.61382400 | 58.69379300 | 104 | H | 53.47550700 | 63.23217700 | 61.37600300 |
| 43 | C | 50.33127000 | 71.86063600 | 58.29160500 | 105 | H | 54.81378300 | 64.31953200 | 61.07497700 |
| 44 | C | 51.62797900 | 72.07810900 | 57.74094000 | 106 | N | 53.37494700 | 65.01401000 | 62.44776800 |
| 45 | H | 51.81733600 | 72.98978600 | 57.18156700 | 107 | H | 53.24100200 | 65.72221000 | 61.70325600 |
| 46 | C | 52.63674300 | 71.17783100 | 57.94460300 | 108 | H | 52.36539200 | 64.77644600 | 62.79094700 |
| 47 | H | 53.63974600 | 71.33749800 | 57.56591100 | 109 | H | 53.87057000 | 65.52275900 | 63.19296600 |
| 48 | C | 52.42713800 | 69.97761300 | 58.71647500 | 110 | O | 53.21743900 | 66.86399700 | 60.33521800 |
| 49 | O | 53.36534000 | 69.17838100 | 58.96701100 | 111 | H | 54.70484300 | 67.79801700 | 67.54347400 |
| 50 | H | 53.17787800 | 67.77163500 | 59.95440500 | 112 | O | 52.53845300 | 66.12113700 | 59.28767200 |
| 51 | C | 51.07242400 | 69.74728000 | 59.21739600 | 113 | H | 51.80201800 | 65.70402000 | 59.81166700 |
| 52 | C | 50.07301500 | 70.68221000 | 58.99165300 | 114 | O | 50.90055400 | 68.62270900 | 59.92363800 |
| 53 | H | 49.08333700 | 70.51797400 | 59.40046900 | 115 | H | 49.97572900 | 68.51562800 | 60.18822800 |
| 54 | C | 54.06032400 | 62.81017500 | 57.10158600 | 116 | H | 49.60119229 | 73.83815517 | 58.71415793 |
| 55 | H | 54.11491900 | 63.89129600 | 56.93646200 | 117 | H | 50.05395741 | 66.68322094 | 56.06347605 |
| 56 | H | 54.68351600 | 62.60614300 | 57.98022800 | 118 | H | 51.39954536 | 69.44912601 | 64.07036426 |
| 57 | C | 52.58787100 | 62.40211100 | 57.35511600 | 119 | H | 57.41536962 | 62.57490839 | 64.85773871 |
| 58 | H | 52.11917200 | 62.17719500 | 56.38734300 | 120 | H | 49.26470474 | 65.36895128 | 66.19586661 |
| 59 | C | 52.43645500 | 61.17236300 | 58.25696400 | 121 | H | 48.20223527 | 64.66411334 | 61.65436883 |
| 60 | H | 51.38009400 | 60.90584900 | 58.36869400 | 122 | H | 54.50552723 | 62.34254522 | 56.24834719 |
| 61 | H | 52.95930100 | 60.28643000 | 57.89545900 | 123 | H | 58.64100475 | 66.81033128 | 54.82325875 |
| 62 | H | 52.81936600 | 61.38879000 | 59.26176400 | 124 | H | 57.00199025 | 68.53996160 | 63.74670705 |

**Cartesian coordinates for the DOPA radical regeneration using HO_2_^•^ as the oxidant in the protonated state of Asp88 (Pathway D).**

| **RC** | | | | | | | | | |
| --- | --- | --- | --- | --- | --- | --- | --- | --- | --- |
| 1 | C | 51.04335600 | 67.09511100 | 56.03028900 | 64 | H | 52.70559400 | 64.15131500 | 59.44198400 |
| 2 | H | 51.46661400 | 66.86223500 | 57.01670700 | 65 | H | 52.26719500 | 64.91374000 | 57.92344300 |
| 3 | H | 50.87173700 | 68.17662300 | 56.01037900 | 66 | H | 51.08840000 | 63.90104300 | 58.76715700 |
| 4 | C | 52.06312100 | 66.70747400 | 54.93178000 | 67 | C | 58.94230200 | 66.33816100 | 55.76834000 |
| 5 | H | 51.74047700 | 67.15809400 | 53.98929700 | 68 | H | 59.75438600 | 66.96732600 | 56.15856800 |
| 6 | C | 53.43856600 | 67.29510400 | 55.25872100 | 69 | H | 59.39699600 | 65.36948500 | 55.54929900 |
| 7 | H | 53.39910800 | 68.37254600 | 55.44657800 | 70 | C | 57.89120100 | 66.18222900 | 56.83906000 |
| 8 | H | 53.88335700 | 66.81094800 | 56.13698200 | 71 | C | 56.92034000 | 67.16362700 | 57.08106200 |
| 9 | H | 54.14101100 | 67.14275800 | 54.43299500 | 72 | H | 56.87995700 | 68.05858500 | 56.46645600 |
| 10 | C | 52.17917400 | 65.19366500 | 54.68863300 | 73 | C | 55.99615700 | 67.01991700 | 58.11340100 |
| 11 | H | 52.52934700 | 64.67669800 | 55.58808300 | 74 | H | 55.28615300 | 67.81441400 | 58.30272000 |
| 12 | H | 51.22331400 | 64.75529100 | 54.39499100 | 75 | C | 55.99868300 | 65.87088400 | 58.90284200 |
| 13 | H | 52.90474500 | 64.98746800 | 53.89542200 | 76 | H | 55.24954800 | 65.75141300 | 59.67672300 |
| 14 | C | 48.26023000 | 65.88492100 | 61.51168800 | 77 | C | 56.95957700 | 64.88877500 | 58.67912600 |
| 15 | H | 47.95368500 | 66.27906400 | 62.48853200 | 78 | H | 56.97456900 | 63.98805600 | 59.28592700 |
| 16 | H | 47.79829000 | 66.52466100 | 60.75906100 | 79 | C | 57.90277000 | 65.05333800 | 57.66710700 |
| 17 | C | 49.74043500 | 66.12120600 | 61.49110200 | 80 | H | 58.66232800 | 64.29263400 | 57.52528600 |
| 18 | O | 50.60352900 | 65.10996700 | 61.79512100 | 81 | C | 56.53924900 | 68.71240200 | 62.76296900 |
| 19 | O | 50.19228700 | 67.23401500 | 61.34656900 | 82 | H | 57.33120500 | 69.16184100 | 62.15865600 |
| 20 | C | 48.87750300 | 64.47300000 | 65.77814500 | 83 | C | 56.07811000 | 67.45233700 | 62.02852200 |
| 21 | H | 48.17638400 | 64.64316400 | 64.94992700 | 84 | H | 56.90389700 | 66.77590200 | 61.78273300 |
| 22 | C | 50.07931500 | 63.71815000 | 65.19523500 | 85 | H | 55.32823400 | 66.90182800 | 62.60366100 |
| 23 | H | 50.61358300 | 64.29091000 | 64.42702400 | 86 | H | 55.62079900 | 67.72292600 | 61.07713600 |
| 24 | H | 50.78546500 | 63.42022100 | 65.97591100 | 87 | C | 55.40351400 | 69.75710000 | 62.88739500 |
| 25 | H | 49.73934900 | 62.78721200 | 64.72816100 | 88 | H | 55.71503600 | 70.56989700 | 63.55267700 |
| 26 | C | 48.18978700 | 63.59074200 | 66.81423600 | 89 | H | 54.53283300 | 69.29286400 | 63.36410100 |
| 27 | H | 47.74825000 | 62.71800800 | 66.32659200 | 90 | C | 54.98671800 | 70.38843600 | 61.55665200 |
| 28 | H | 48.90540800 | 63.19151800 | 67.53782200 | 91 | H | 54.20841500 | 71.14371600 | 61.70941900 |
| 29 | H | 47.40459300 | 64.11473700 | 67.35169300 | 92 | H | 55.84053000 | 70.88347800 | 61.08804700 |
| 30 | C | 51.68837200 | 68.40979600 | 63.98283500 | 93 | H | 54.60212900 | 69.65844800 | 60.84258900 |
| 31 | H | 52.07240000 | 68.30558700 | 62.96690700 | 94 | C | 57.48812600 | 62.84496900 | 63.83341900 |
| 32 | H | 50.83281600 | 67.73102400 | 64.05809800 | 95 | H | 57.45807500 | 61.93247100 | 63.23059500 |
| 33 | C | 52.77283500 | 67.98613100 | 64.93594000 | 96 | H | 58.48231700 | 63.26299400 | 63.67840800 |
| 34 | N | 53.54907100 | 66.86874700 | 64.65503000 | 97 | C | 56.39427100 | 63.81005300 | 63.33592900 |
| 35 | C | 54.43781700 | 66.76120700 | 65.62543700 | 98 | H | 56.81516900 | 64.47246300 | 62.57314200 |
| 36 | H | 55.19379000 | 65.99392500 | 65.68887600 | 99 | H | 56.04376800 | 64.47106700 | 64.13784600 |
| 37 | N | 54.26099500 | 67.73284000 | 66.54800000 | 100 | C | 55.20158200 | 63.07435400 | 62.71810300 |
| 38 | C | 53.21046000 | 68.52139100 | 66.12424700 | 101 | H | 54.73286100 | 62.40000100 | 63.44591300 |
| 39 | H | 52.88924000 | 69.37391400 | 66.70346800 | 102 | H | 55.57040200 | 62.41839800 | 61.92644300 |
| 40 | C | 49.32706100 | 72.98367500 | 58.27481500 | 103 | C | 54.14807500 | 63.94906800 | 62.05150000 |
| 41 | H | 48.98863500 | 73.28229100 | 57.27425100 | 104 | H | 53.50215400 | 63.33624000 | 61.41872000 |
| 42 | H | 48.45523300 | 72.58828800 | 58.80093100 | 105 | H | 54.59123700 | 64.72423200 | 61.42893100 |
| 43 | C | 50.41500200 | 71.94787400 | 58.17433300 | 106 | N | 53.23021100 | 64.66042300 | 63.01359100 |
| 44 | C | 51.66798800 | 72.26780400 | 57.63275100 | 107 | H | 53.55946600 | 65.56089500 | 63.48115800 |
| 45 | H | 51.85281100 | 73.25094500 | 57.20922700 | 108 | H | 52.36126500 | 64.91882700 | 62.52382600 |
| 46 | C | 52.70020300 | 71.33779700 | 57.61108000 | 109 | H | 52.98364800 | 64.02071900 | 63.77956600 |
| 47 | H | 53.67555700 | 71.58832700 | 57.20950600 | 110 | O | 53.00131500 | 67.82799700 | 60.96025800 |
| 48 | C | 52.49326900 | 70.05723800 | 58.10480600 | 111 | H | 52.37349100 | 68.11317500 | 60.23030200 |
| 49 | O | 53.52505500 | 69.14998000 | 58.10900100 | 112 | H | 54.79644900 | 67.84368800 | 67.41052700 |
| 50 | H | 53.18458600 | 68.27269200 | 57.88141200 | 113 | O | 53.11065900 | 66.51785900 | 60.78386700 |
| 51 | C | 51.24921400 | 69.73224600 | 58.65075500 | 114 | H | 50.20643500 | 64.20515200 | 61.81358900 |
| 52 | C | 50.21719900 | 70.66096800 | 58.68186100 | 115 | O | 51.13893900 | 68.44732300 | 59.15844500 |
| 53 | H | 49.25672400 | 70.39124500 | 59.11238700 | 116 | H | 50.35629300 | 68.34341000 | 59.72352900 |
| 54 | C | 54.15741600 | 62.92268600 | 57.39005700 | 117 | H | 54.47905792 | 62.42190932 | 56.50086119 |
| 55 | H | 54.37336500 | 63.98542000 | 57.24115000 | 118 | H | 58.63548982 | 66.77021249 | 54.83877139 |
| 56 | H | 54.79763900 | 62.60146600 | 58.22047100 | 119 | H | 56.96318311 | 68.51656831 | 63.72568860 |
| 57 | C | 52.65885800 | 62.76314100 | 57.75293800 | 120 | H | 57.43166602 | 62.55073918 | 64.86061961 |
| 58 | H | 52.08006700 | 62.69899900 | 56.82287300 | 121 | H | 51.33071826 | 69.41295910 | 64.08602220 |
| 59 | C | 52.35263000 | 61.50974200 | 58.58067100 | 122 | H | 49.16653231 | 65.44362139 | 66.12347986 |
| 60 | H | 51.28709100 | 61.46108800 | 58.82497300 | 123 | H | 49.64914737 | 73.87065942 | 58.77921465 |
| 61 | H | 52.60845800 | 60.58006500 | 58.06760500 | 124 | H | 50.09134844 | 66.60809701 | 55.99288601 |
| 62 | H | 52.91353900 | 61.52465200 | 59.52512300 | 125 | H | 47.87364229 | 64.89042936 | 61.43146133 |
| 63 | C | 52.15094400 | 63.99966200 | 58.50932200 |  |  |  |  |  |

| **TS** | | | | | | | | | |
| --- | --- | --- | --- | --- | --- | --- | --- | --- | --- |
| 1 | C | 51.01628300 | 67.06549600 | 55.94266900 | 64 | H | 52.76786000 | 64.23685200 | 59.40767600 |
| 2 | H | 51.44463300 | 66.89620500 | 56.93766400 | 65 | H | 52.26523500 | 64.93898900 | 57.88690400 |
| 3 | H | 50.83893900 | 68.14418700 | 55.86587500 | 66 | H | 51.13314300 | 63.92912400 | 58.79433900 |
| 4 | C | 52.04036100 | 66.63532000 | 54.86643000 | 67 | C | 58.99006900 | 66.34842000 | 55.74309300 |
| 5 | H | 51.67077500 | 66.94347800 | 53.88380400 | 68 | H | 59.79664200 | 66.98906100 | 56.12675200 |
| 6 | C | 53.37136200 | 67.35414500 | 55.09785000 | 69 | H | 59.45966200 | 65.39102700 | 55.50721300 |
| 7 | H | 53.25020700 | 68.44052400 | 55.12681600 | 70 | C | 57.95858000 | 66.15911400 | 56.82289200 |
| 8 | H | 53.83301600 | 67.05260500 | 56.04312000 | 71 | C | 56.94374400 | 67.09597700 | 57.05738600 |
| 9 | H | 54.08503900 | 67.13081600 | 54.29879400 | 72 | H | 56.85447000 | 67.97879700 | 56.43013300 |
| 10 | C | 52.27032000 | 65.11768100 | 54.78953000 | 73 | C | 56.03101300 | 66.91844800 | 58.09249700 |
| 11 | H | 52.70192500 | 64.74411100 | 55.72262000 | 74 | H | 55.27034000 | 67.66790000 | 58.26395600 |
| 12 | H | 51.34801700 | 64.56556900 | 54.59685800 | 75 | C | 56.09477400 | 65.77890400 | 58.89377500 |
| 13 | H | 52.98060100 | 64.87687600 | 53.99200200 | 76 | H | 55.35166300 | 65.63574500 | 59.66677500 |
| 14 | C | 48.24194500 | 65.85729100 | 61.53191400 | 77 | C | 57.09635200 | 64.83878700 | 58.67277300 |
| 15 | H | 47.91363800 | 66.25730500 | 62.49815800 | 78 | H | 57.14875000 | 63.94202600 | 59.28379600 |
| 16 | H | 47.80133900 | 66.50041000 | 60.76767200 | 79 | C | 58.02827900 | 65.03832600 | 57.65728300 |
| 17 | C | 49.71923000 | 66.09066400 | 61.54819500 | 80 | H | 58.82234600 | 64.31356600 | 57.51430300 |
| 18 | O | 50.62299300 | 65.08290600 | 61.60205200 | 81 | C | 56.51393100 | 68.69307900 | 62.78422200 |
| 19 | O | 50.14234900 | 67.22962100 | 61.60512900 | 82 | H | 57.29468600 | 69.12813000 | 62.15500400 |
| 20 | C | 48.80659600 | 64.49032300 | 65.76219600 | 83 | C | 56.03596800 | 67.41235800 | 62.09706400 |
| 21 | H | 47.94073100 | 64.66877200 | 65.11378700 | 84 | H | 56.86292800 | 66.75130000 | 61.81309800 |
| 22 | C | 49.88323500 | 63.84890700 | 64.87387700 | 85 | H | 55.34538700 | 66.85883600 | 62.74050300 |
| 23 | H | 50.03276100 | 64.39415800 | 63.93400800 | 86 | H | 55.50218600 | 67.63831600 | 61.17419900 |
| 24 | H | 50.84414900 | 63.74688700 | 65.38432400 | 87 | C | 55.38251100 | 69.74057600 | 62.91391200 |
| 25 | H | 49.58777400 | 62.83190000 | 64.60028400 | 88 | H | 55.72880100 | 70.58991900 | 63.51320200 |
| 26 | C | 48.37614100 | 63.51361400 | 66.85162900 | 89 | H | 54.54407700 | 69.30360200 | 63.46759800 |
| 27 | H | 47.90456600 | 62.63548300 | 66.40139400 | 90 | C | 54.87919800 | 70.29015700 | 61.57588000 |
| 28 | H | 49.23083000 | 63.13982000 | 67.42485600 | 91 | H | 54.12848000 | 71.07193000 | 61.73490900 |
| 29 | H | 47.66004000 | 63.95901100 | 67.53766400 | 92 | H | 55.70427900 | 70.73515700 | 61.01253000 |
| 30 | C | 51.74971100 | 68.49375000 | 64.05002000 | 93 | H | 54.42759900 | 69.51946400 | 60.94824100 |
| 31 | H | 52.19813800 | 68.42753300 | 63.05701700 | 94 | C | 57.31983800 | 62.83314600 | 63.87153000 |
| 32 | H | 50.90678300 | 67.79555700 | 64.06035700 | 95 | H | 57.16467000 | 61.91645100 | 63.29556700 |
| 33 | C | 52.79044800 | 68.06363000 | 65.04271400 | 96 | H | 58.31343700 | 63.19015700 | 63.59764300 |
| 34 | N | 53.53807800 | 66.92261400 | 64.78974400 | 97 | C | 56.23307400 | 63.87027700 | 63.48578000 |
| 35 | C | 54.41382300 | 66.80586400 | 65.77029500 | 98 | H | 56.68834000 | 64.70573000 | 62.94794100 |
| 36 | H | 55.14648100 | 66.01859000 | 65.84910200 | 99 | H | 55.77249900 | 64.31376000 | 64.37893100 |
| 37 | N | 54.25046700 | 67.79454000 | 66.67432900 | 100 | C | 55.13360300 | 63.26953900 | 62.60033100 |
| 38 | C | 53.22630900 | 68.60569300 | 66.22747000 | 101 | H | 54.63844500 | 62.43705700 | 63.11981100 |
| 39 | H | 52.92137100 | 69.47686900 | 66.78666400 | 102 | H | 55.58989800 | 62.82453000 | 61.71303500 |
| 40 | C | 49.39579700 | 72.87508000 | 58.29686200 | 103 | C | 54.07517800 | 64.23117000 | 62.06843500 |
| 41 | H | 49.12897800 | 73.12501000 | 57.26138800 | 104 | H | 53.42607600 | 63.70575100 | 61.36669200 |
| 42 | H | 48.47819800 | 72.54072500 | 58.78553300 | 105 | H | 54.49723300 | 65.09341700 | 61.55350900 |
| 43 | C | 50.42517000 | 71.77910600 | 58.30460300 | 106 | N | 53.17596000 | 64.77599700 | 63.15416400 |
| 44 | C | 51.74175700 | 71.99072800 | 57.82875800 | 107 | H | 53.46529000 | 65.68967000 | 63.65302100 |
| 45 | H | 52.02067900 | 72.96348100 | 57.43592900 | 108 | H | 52.23570800 | 64.94082300 | 62.77517200 |
| 46 | C | 52.68289000 | 70.98175400 | 57.86252900 | 109 | H | 53.08364500 | 64.06288000 | 63.89122200 |
| 47 | H | 53.69862200 | 71.13802300 | 57.51820700 | 110 | O | 52.85913800 | 67.39746400 | 61.22483400 |
| 48 | C | 52.34377600 | 69.69260900 | 58.33348300 | 111 | H | 51.90210800 | 67.38105500 | 61.01574500 |
| 49 | O | 53.22124000 | 68.71505700 | 58.29317000 | 112 | H | 54.78522400 | 67.90267500 | 67.53667200 |
| 50 | H | 53.14507600 | 67.94107200 | 59.06630400 | 113 | O | 53.46388300 | 67.07619800 | 60.00926300 |
| 51 | C | 51.01418500 | 69.48208200 | 58.80153500 | 114 | H | 50.25670700 | 64.16572700 | 61.52355600 |
| 52 | C | 50.08789200 | 70.51492800 | 58.79244700 | 115 | O | 50.72718000 | 68.23160700 | 59.24715600 |
| 53 | H | 49.08120200 | 70.32768500 | 59.15273000 | 116 | H | 49.96661800 | 68.25826600 | 59.84501500 |
| 54 | C | 54.18893500 | 62.93929900 | 57.39493200 | 117 | H | 49.70612011 | 73.78578704 | 58.76506308 |
| 55 | H | 54.41046500 | 64.00031200 | 57.24419200 | 118 | H | 57.35644364 | 62.54876702 | 64.90239790 |
| 56 | H | 54.83412600 | 62.61624600 | 58.22069000 | 119 | H | 56.95160860 | 68.50925728 | 63.74315260 |
| 57 | C | 52.69282800 | 62.78679900 | 57.77129400 | 120 | H | 49.12903052 | 65.44986135 | 66.10892664 |
| 58 | H | 52.10756800 | 62.69571800 | 56.84720000 | 121 | H | 47.85295617 | 64.86486717 | 61.43873338 |
| 59 | C | 52.40154700 | 61.55047800 | 58.62843100 | 122 | H | 50.06506285 | 66.57605829 | 55.91962652 |
| 60 | H | 51.33997600 | 61.51091100 | 58.89178900 | 123 | H | 58.65834533 | 66.78307133 | 54.82334432 |
| 61 | H | 52.64851500 | 60.61101000 | 58.12929300 | 124 | H | 51.36381083 | 69.48661698 | 64.15099634 |
| 62 | H | 52.97484800 | 61.58430900 | 59.56523800 | 125 | H | 54.50011490 | 62.43460423 | 56.50422988 |
| 63 | C | 52.18621800 | 64.04026000 | 58.50015900 |  |  |  |  |  |

| **PC** | | | | | | | | | |
| --- | --- | --- | --- | --- | --- | --- | --- | --- | --- |
| 1 | C | 51.01908300 | 67.11949200 | 56.03236400 | 64 | H | 52.53827500 | 64.12451700 | 59.35377900 |
| 2 | H | 51.44405800 | 66.91445400 | 57.02255600 | 65 | H | 52.11141100 | 64.83459500 | 57.81283900 |
| 3 | H | 50.83918800 | 68.20012600 | 55.99470000 | 66 | H | 50.96236700 | 63.77048100 | 58.63459800 |
| 4 | C | 52.05069100 | 66.73338700 | 54.94470100 | 67 | C | 58.99809100 | 66.38347900 | 55.75050000 |
| 5 | H | 51.74178700 | 67.18655100 | 53.99861000 | 68 | H | 59.80688600 | 67.02389900 | 56.12930700 |
| 6 | C | 53.42096800 | 67.31799800 | 55.29616700 | 69 | H | 59.45930900 | 65.41671400 | 55.53612200 |
| 7 | H | 53.37758100 | 68.39777900 | 55.46957400 | 70 | C | 57.94687100 | 66.22752900 | 56.81521000 |
| 8 | H | 53.83315400 | 66.85772200 | 56.19958800 | 71 | C | 56.94693400 | 67.18853200 | 57.01439600 |
| 9 | H | 54.14168100 | 67.14875900 | 54.48939800 | 72 | H | 56.89791500 | 68.07219400 | 56.38416800 |
| 10 | C | 52.16764400 | 65.22060900 | 54.69206600 | 73 | C | 55.99039000 | 67.02976500 | 58.01108300 |
| 11 | H | 52.51318800 | 64.69688600 | 55.58892100 | 74 | H | 55.22971600 | 67.78896800 | 58.14271600 |
| 12 | H | 51.21346500 | 64.78382000 | 54.38893100 | 75 | C | 56.00310400 | 65.88942900 | 58.81335800 |
| 13 | H | 52.89847400 | 65.02216100 | 53.90191500 | 76 | H | 55.21446300 | 65.75686500 | 59.53926500 |
| 14 | C | 48.20955500 | 65.86259800 | 61.53695600 | 77 | C | 56.99869300 | 64.93276100 | 58.63871300 |
| 15 | H | 47.92505400 | 66.22639600 | 62.53216000 | 78 | H | 57.01252700 | 64.03788200 | 59.25499700 |
| 16 | H | 47.72168300 | 66.51941700 | 60.81562100 | 79 | C | 57.96812400 | 65.10894500 | 57.65468800 |
| 17 | C | 49.67991800 | 66.11298500 | 61.47856800 | 80 | H | 58.74648300 | 64.36365800 | 57.53228600 |
| 18 | O | 50.58842400 | 65.13241500 | 61.61378600 | 81 | C | 56.48441300 | 68.71637600 | 62.79100300 |
| 19 | O | 50.08716000 | 67.26205100 | 61.41594000 | 82 | H | 57.26047900 | 69.15197300 | 62.15592500 |
| 20 | C | 48.86967000 | 64.49184900 | 65.76124900 | 83 | C | 55.98734600 | 67.44792700 | 62.09562000 |
| 21 | H | 48.11976700 | 64.67800600 | 64.97996500 | 84 | H | 56.80663000 | 66.78111300 | 61.80537200 |
| 22 | C | 50.04436500 | 63.77406600 | 65.08247100 | 85 | H | 55.29639300 | 66.89907700 | 62.74047000 |
| 23 | H | 50.53572500 | 64.38341600 | 64.31244300 | 86 | H | 55.45580300 | 67.69265700 | 61.17479100 |
| 24 | H | 50.79927100 | 63.45019700 | 65.80387500 | 87 | C | 55.36407200 | 69.77229400 | 62.95388200 |
| 25 | H | 49.68776800 | 62.86084300 | 64.59368100 | 88 | H | 55.74598600 | 70.62747100 | 63.52168900 |
| 26 | C | 48.24516500 | 63.57203300 | 66.80546900 | 89 | H | 54.55080100 | 69.35218400 | 63.55694900 |
| 27 | H | 47.80187000 | 62.69973300 | 66.31780200 | 90 | C | 54.79521900 | 70.30506000 | 61.63515200 |
| 28 | H | 48.99788800 | 63.17661100 | 67.49322000 | 91 | H | 54.09579500 | 71.12745200 | 61.82231200 |
| 29 | H | 47.46837700 | 64.06633000 | 67.38324300 | 92 | H | 55.59558000 | 70.69153200 | 60.99666800 |
| 30 | C | 51.74891800 | 68.41640600 | 64.02646300 | 93 | H | 54.25879200 | 69.54020800 | 61.06947700 |
| 31 | H | 52.19781200 | 68.35979500 | 63.03175200 | 94 | C | 57.41155300 | 62.81472500 | 63.86878000 |
| 32 | H | 50.89980400 | 67.72557100 | 64.02659600 | 95 | H | 57.30648700 | 61.89504900 | 63.28607500 |
| 33 | C | 52.78917700 | 67.96773800 | 65.01629700 | 96 | H | 58.41566100 | 63.18377800 | 63.65428500 |
| 34 | N | 53.52230600 | 66.81209200 | 64.77491300 | 97 | C | 56.34817500 | 63.83305300 | 63.40440700 |
| 35 | C | 54.40146400 | 66.70855000 | 65.75228900 | 98 | H | 56.82816000 | 64.56179400 | 62.74422000 |
| 36 | H | 55.12160600 | 65.91085600 | 65.84928300 | 99 | H | 55.94991400 | 64.41593900 | 64.24361800 |
| 37 | N | 54.25294100 | 67.71310600 | 66.64538000 | 100 | C | 55.18326500 | 63.20463300 | 62.62674900 |
| 38 | C | 53.23393000 | 68.52479600 | 66.19100300 | 101 | H | 54.53346200 | 62.60472300 | 63.27344300 |
| 39 | H | 52.93594700 | 69.40451800 | 66.74093000 | 102 | H | 55.58885800 | 62.49627200 | 61.90092200 |
| 40 | C | 49.34880400 | 72.97135400 | 58.24295800 | 103 | C | 54.37819100 | 64.20386800 | 61.80396700 |
| 41 | H | 49.08476900 | 73.25551900 | 57.21523800 | 104 | H | 53.81525900 | 63.70319300 | 61.01428800 |
| 42 | H | 48.43671200 | 72.60722600 | 58.71938700 | 105 | H | 55.04574000 | 64.91805000 | 61.32431900 |
| 43 | C | 50.39942400 | 71.90442700 | 58.22100200 | 106 | N | 53.37092600 | 65.02323000 | 62.56260700 |
| 44 | C | 51.71277300 | 72.17516400 | 57.72960300 | 107 | H | 53.67379200 | 65.52477000 | 63.43328700 |
| 45 | H | 51.93301800 | 73.15772000 | 57.32367700 | 108 | H | 53.02466400 | 65.80591300 | 61.95195200 |
| 46 | C | 52.69921700 | 71.22889600 | 57.78123300 | 109 | H | 52.56395300 | 64.44808700 | 62.81110700 |
| 47 | H | 53.71017000 | 71.43246600 | 57.44835700 | 110 | O | 52.79195700 | 67.29671300 | 61.12591200 |
| 48 | C | 52.44171400 | 69.91303900 | 58.30815700 | 111 | H | 51.81487300 | 67.36127900 | 61.04188400 |
| 49 | O | 53.34287200 | 69.03780400 | 58.41454700 | 112 | H | 54.78647100 | 67.82915900 | 67.50739200 |
| 50 | H | 53.18398200 | 67.57077500 | 59.27227000 | 113 | O | 53.15540400 | 66.74542300 | 59.82875400 |
| 51 | C | 51.07494800 | 69.64649100 | 58.74692200 | 114 | H | 50.23246500 | 64.20923900 | 61.61845000 |
| 52 | C | 50.10545600 | 70.63387600 | 58.71766100 | 115 | O | 50.84815800 | 68.40188500 | 59.18829600 |
| 53 | H | 49.10753100 | 70.41686700 | 59.08725100 | 116 | H | 50.01191100 | 68.34592800 | 59.67480100 |
| 54 | C | 54.11920000 | 62.91790400 | 57.36456600 | 117 | H | 49.65234802 | 73.86650210 | 58.74442675 |
| 55 | H | 54.30187700 | 63.98685800 | 57.21585300 | 118 | H | 50.06982402 | 66.62719521 | 55.99426861 |
| 56 | H | 54.75252300 | 62.61985700 | 58.20894600 | 119 | H | 58.67364037 | 66.80406253 | 54.82166132 |
| 57 | C | 52.61868400 | 62.70088800 | 57.69298400 | 120 | H | 54.47427926 | 62.42356699 | 56.48453780 |
| 58 | H | 52.07024300 | 62.58827800 | 56.74906200 | 121 | H | 57.39579981 | 62.53572458 | 64.90164524 |
| 59 | C | 52.34889400 | 61.45444100 | 58.54256200 | 122 | H | 49.16912606 | 65.45461225 | 66.11945704 |
| 60 | H | 51.28035500 | 61.36544900 | 58.75817700 | 123 | H | 51.37197464 | 69.41205737 | 64.13366401 |
| 61 | H | 52.66287700 | 60.52510700 | 58.06400400 | 124 | H | 56.93092413 | 68.52007687 | 63.74336557 |
| 62 | H | 52.87662600 | 61.52225600 | 59.50372900 | 125 | H | 47.83344478 | 64.86621208 | 61.43372718 |
| 63 | C | 52.02570100 | 63.92357000 | 58.40646800 |  |  |  |  |  |

1. ^*^ Corresponding author. Email: fengwei[@mail.buct.edu.cn](mailto:2005500011@mail.buct.edu.cn). [↑](#footnote-ref-2)
